# Supplementary figures and images for: A noncanonical chaperone interacts with drug efflux pumps during their assembly into bacterial outer membranes
Source: PLoS Biol. 2022 Jan 21;20(1):e3001523. doi: 10.1371/journal.pbio.3001523 (PMC8809574; doi:10.1371/journal.pbio.3001523)

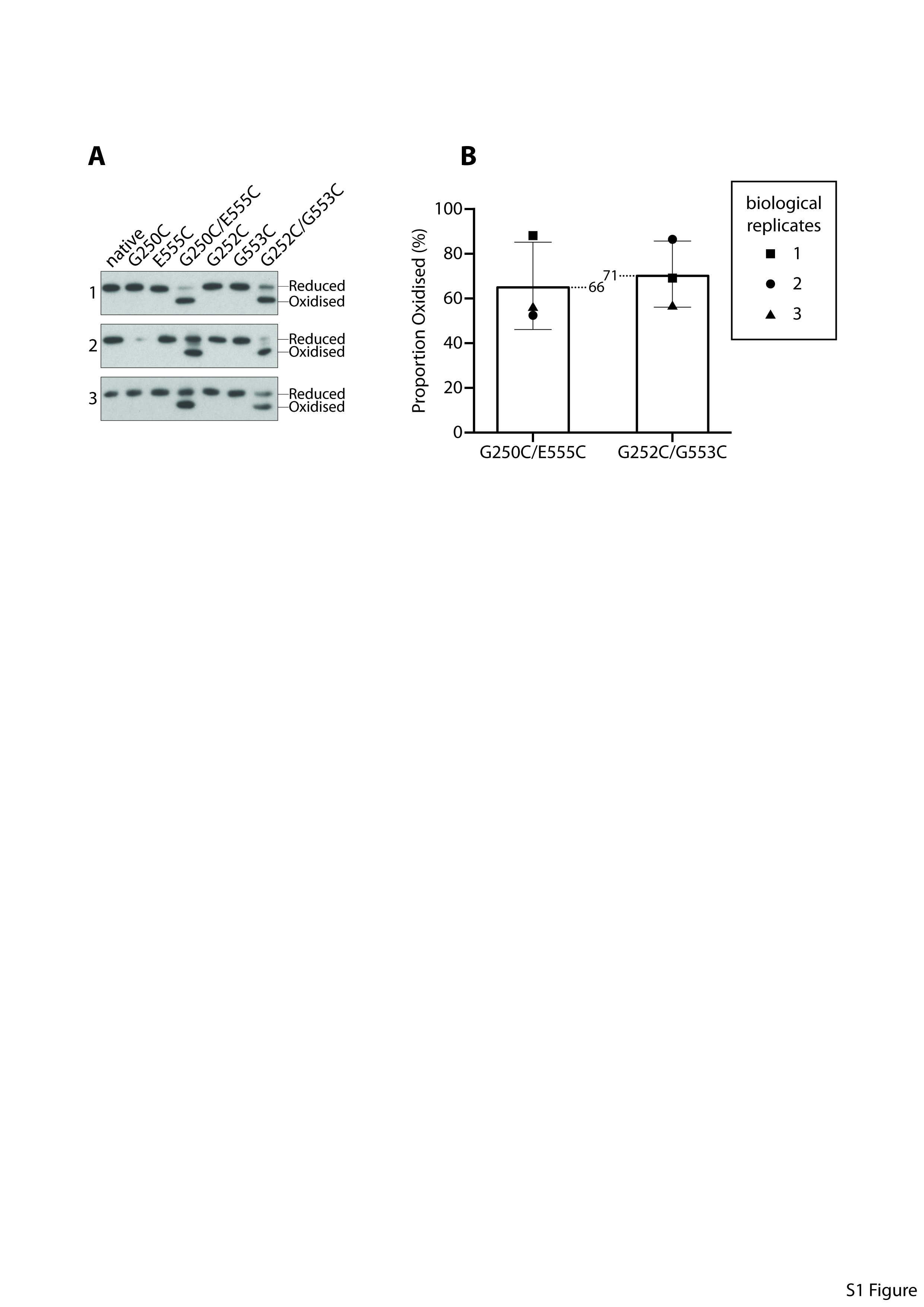

Supplement: S1 Fig — (A) Cells prepared for pulse chase analysis were subjected to nonreducing SDS-PAGE and immunoblotting using antibodies raised against TamA. TamA oxidation states are indicated, based on their migration rates. Biological replicate number is indicated to the left of immunoblots. Uncropped images are presented in S1 Raw Images; original immunoblots are presented in S3 Raw Images. (B) Densitometry of “lockable” TamA was determined using ImageJ 1.51r. Values displayed are equal to the density for oxidised species as a percentage of both oxidised and reduced species. Each biological replicate (n = 3) is shown, with mean indicated by dashed lines and error bars representing standard deviation. Data underlying this figure are presented in S2 Data. (TIF) [file pbio.3001523.s003.tif]

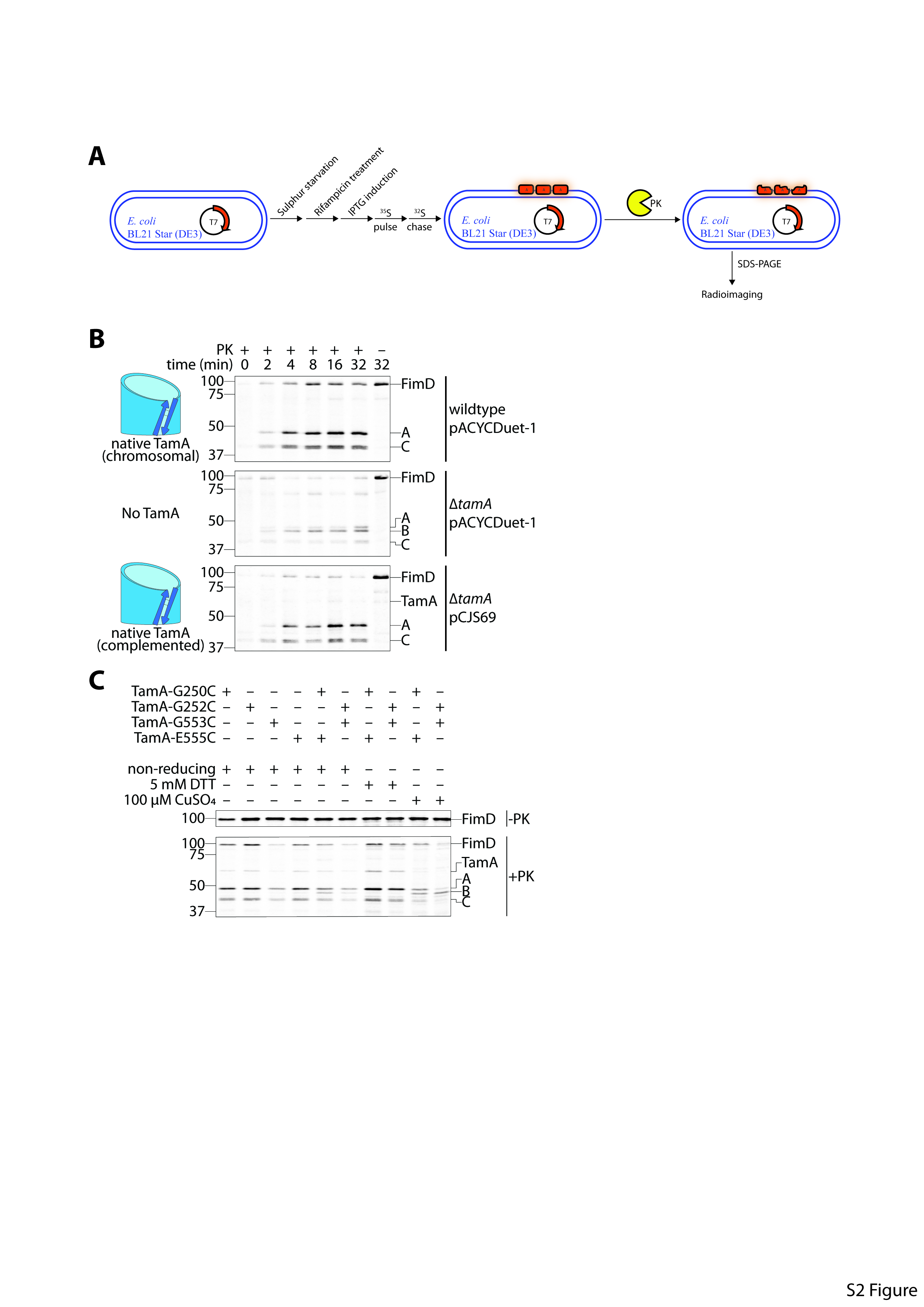

Supplement: S2 Fig — (A) Schematic of the pulse chase experiment based on [38]. E. coli BL21 Star (DE3) cells harbouring a plasmid with the gene of interest (orange arrow) are subjected to sulphur starvation to deplete sulphur-containing amino acids. Cells are then subjected to rifampicin treatment (1 h) to block native RNA transcription before IPTG induction (5 min) allows transcription from the T7 RNA polymerase (which is not sensitive to rifampicin) promoter upstream from the plasmid-encoded gene of interest. Cells are pulsed (45 s) with [35S]-methionine and [35S]-cysteine before chase media (containing [32S]-methionine and [32S]-cysteine) is added. On addition of extracellular protease (PK, yellow), protease-sensitive radiolabelled proteins can be detected once they are localised to the outer membrane through the accumulation of degradation products or a reduction in full-length protein. (B) FimD assembly was monitored over time by pulse chase analysis in the indicated strains of E. coli BL21 Star (DE3) harbouring pKS02 (fimD expression vector) and either pACYCDuet-1 (base vector) or pCJS69 (tamA complementation vector). (C) FimD assembly was monitored as per panel B, except all strains were E. coli BL21 Star (DE3) ΔtamA harbouring pKS02 and the plasmid encoding the indicated TamA cysteine mutants. Media were supplemented with the indicated reducing or oxidising agent, or they were not supplemented (i.e., nonreducing) as indicated. (B-C) Aliquots were taken at 10 s (0 min), 2, 4, 8, 16, and 32 min (panel B) or 8 min only (panel C) and treated with (+) or without (−) proteinase K. Total protein was analysed by SDS-PAGE and storage phosphor imaging. The position of FimD, TamA, and its fragments A, B, and C are indicated to the right of the autoradiograms, and protein standards are indicated on the left (sizes are in kDa). The presence of native TamA is indicated as a cartoon to the left (panel B only). (B-C) Uncropped images are presented in S1 Raw Images; original autoradiographs (in [file pbio.3001523.s004.tif]

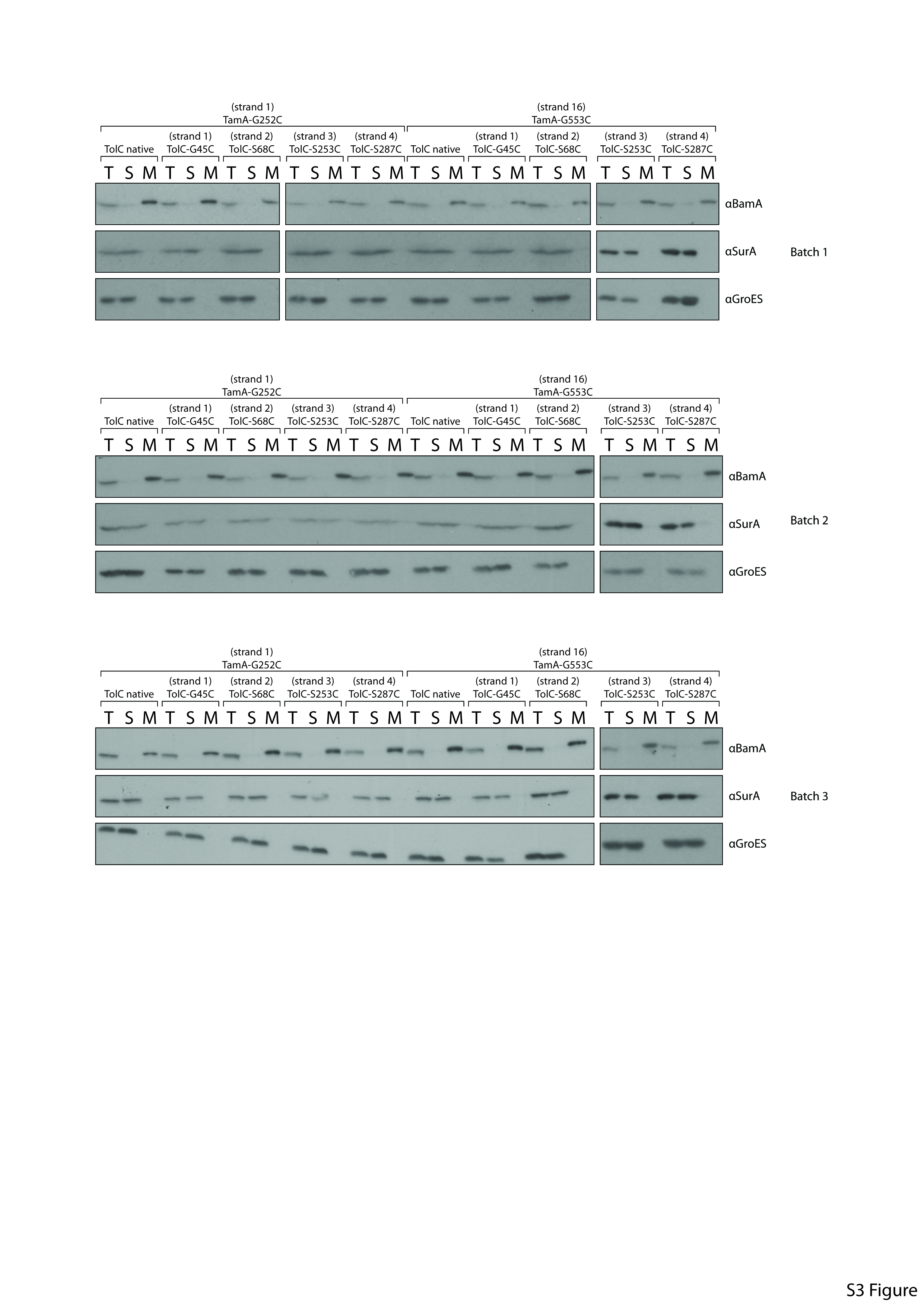

Supplement: S3 Fig — During membrane isolation [58], 1 mL aliquots were taken immediately before ultracentrifugation (Total = “T”) and 1 mL aliquots of the supernatant were taken after ultracentrifugation (Supernatant = “S”). These aliquots were subjected to TCA precipitation and washed with acetone before resuspension in 100 μL SDS loading buffer. Membranes (“M”) were diluted in SDS loading buffer so that the final protein concentration was 1 μg/μL. Samples were analysed by 10%, 12%, or 16% SDS-PAGE and immunoblotting to determine purity of membranes. The membrane protein control (αBamA) was found only in the total and membrane lanes, whereas the periplasmic control (αSurA) and cytoplasmic control (αGroES) were found only in the total and supernatant lanes. Uncropped images are presented in S1 Raw Images; original immunoblots are presented in S3 Raw Images. (TIF) [file pbio.3001523.s005.tif]

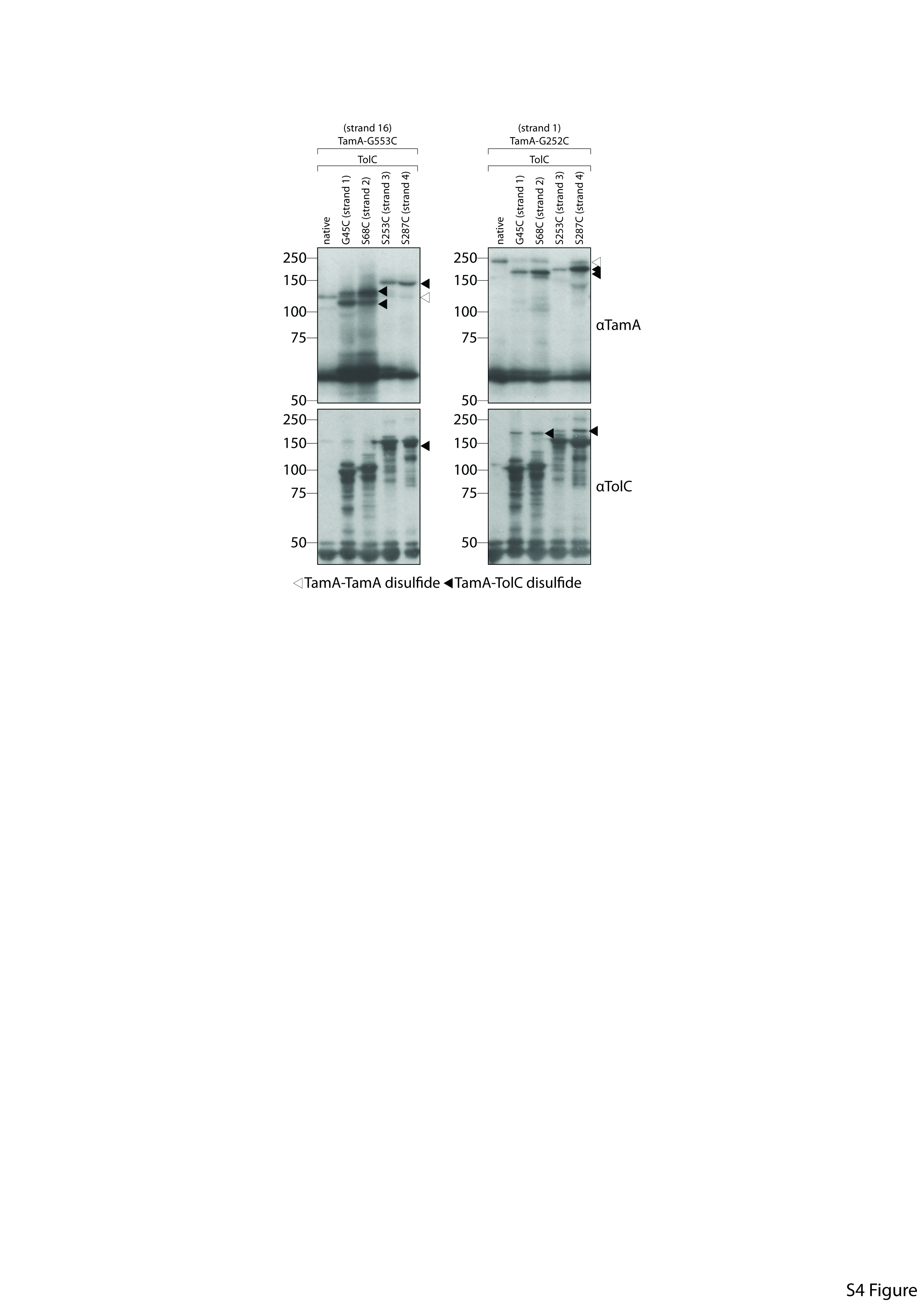

Supplement: S4 Fig — Total membranes were analysed by 8% nonreducing SDS-PAGE and immunoblotting using the indicated antibodies. Uncropped images are presented in S1 Raw Images; original immunoblots are presented in S3 Raw Images. (TIF) [file pbio.3001523.s006.tif]

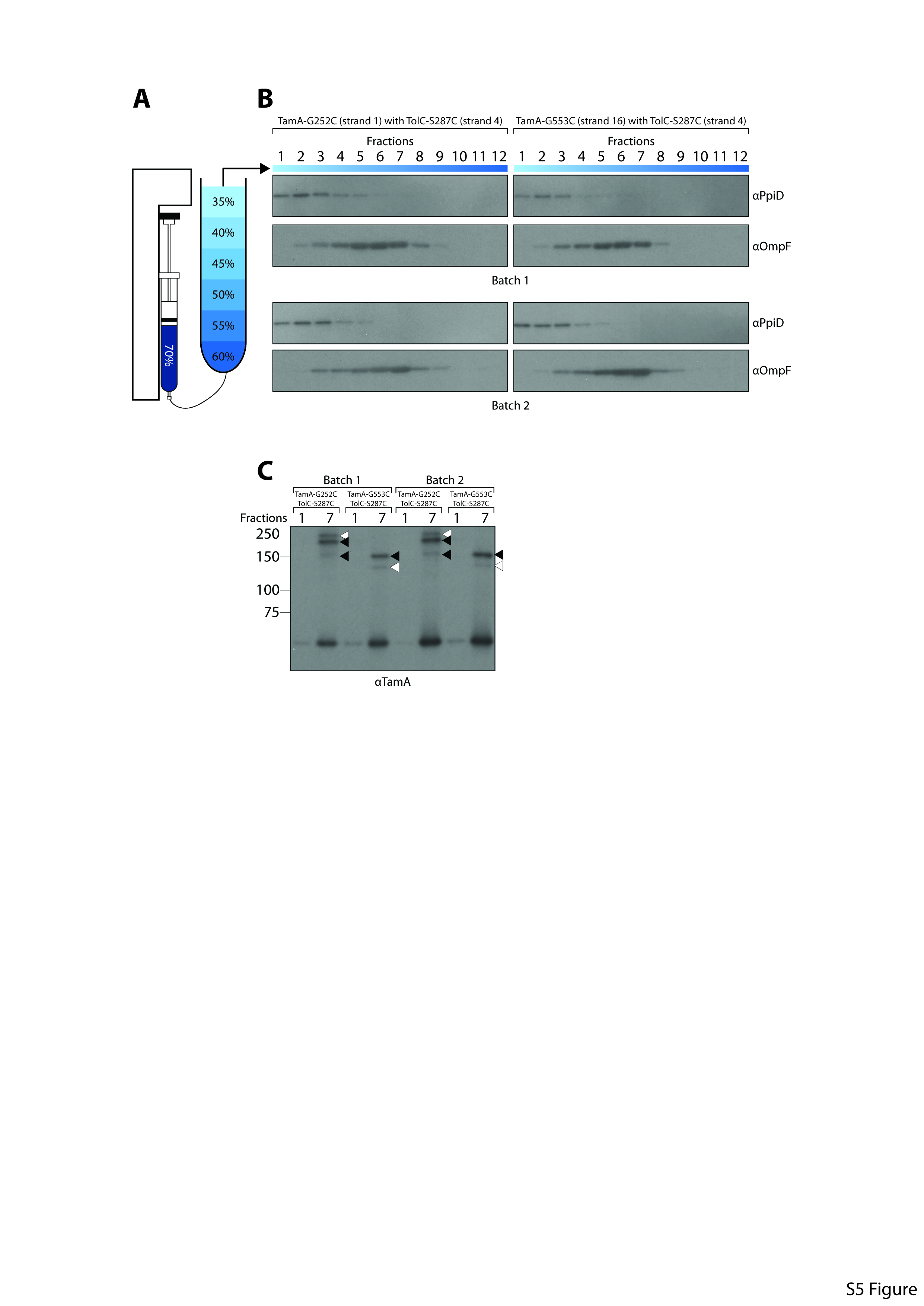

Supplement: S5 Fig — (A) Schematic of sucrose density fractionation. Membranes were isolated and subjected to a 6-step sucrose gradient as indicated (60%–35% w/w sucrose) by ultracentrifugation (200,000 ×g, 17 h, 4°C). Twelve 1 mL fractions were then obtained using 70% w/w sucrose as the displacing fluid as indicated. (B) Fractions were analysed by 10% SDS-PAGE and immunoblotting for the inner membrane (αPpiD) or the outer membrane (αOmpF). (C) Fractions 1 and 7 were subjected to nonreducing SDS-PAGE as per Fig 3C and 3D. Black triangles correspond to TamA–TolC interactions, whereas white triangles correspond to TamA–TamA interactions, as per Fig 3C and 3D. (B-C) Uncropped images are presented in S1 Raw Images; original immunoblots are presented in S3 Raw Images. (TIF) [file pbio.3001523.s007.tif]

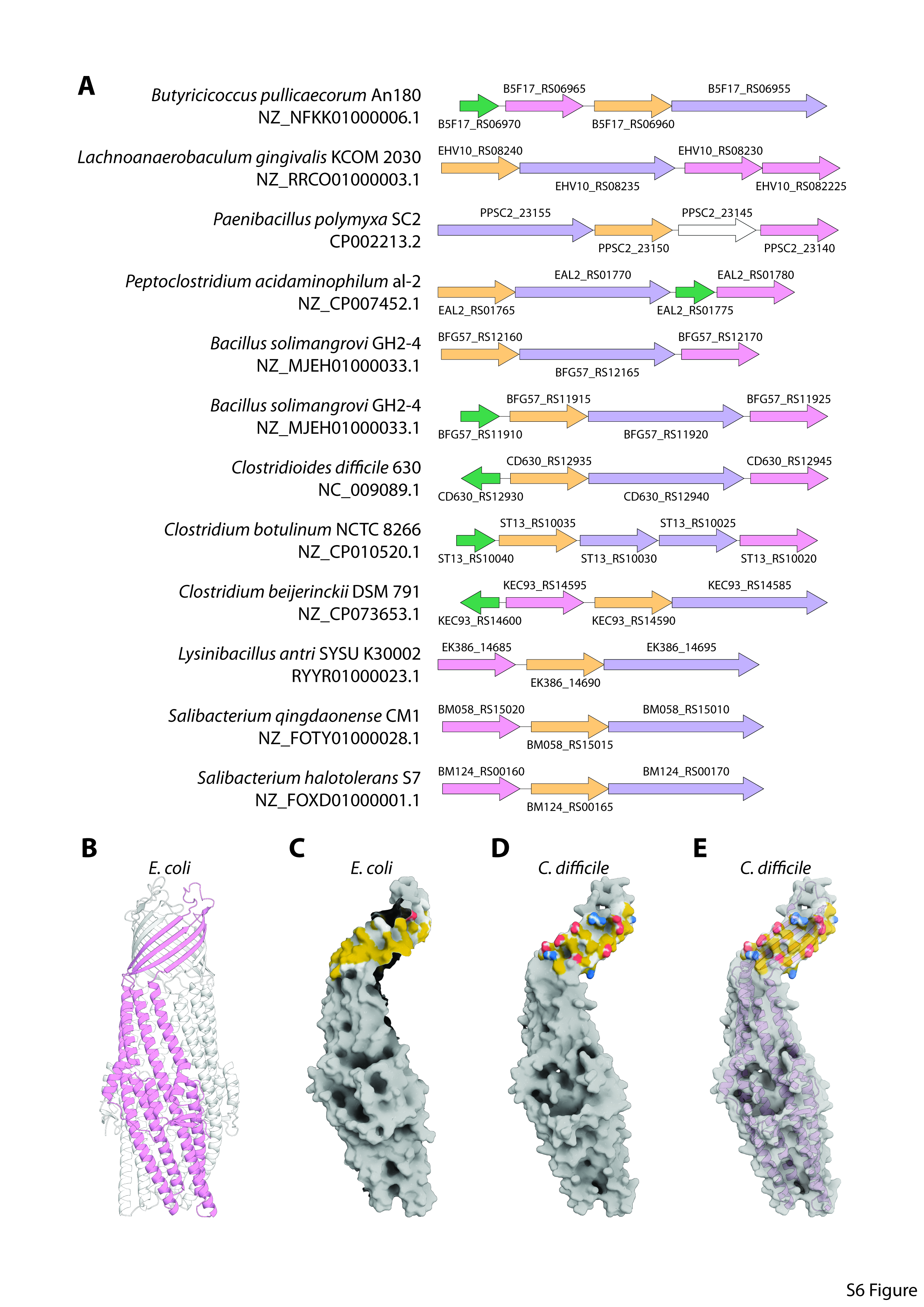

Supplement: S6 Fig — (A) The genetic organisation of the 12 selected efflux pumps depicted in Fig 4B are shown (not to scale). The NCBI accession number is shown below the indicated bacterial strains, and the locus tags for each gene are also shown. Green (regulatory protein), pink (TolC-like protein), yellow (periplasmic adaptor protein), purple (cytoplasmic membrane channel component), white (hypothetical protein). The L. gingivalis operon encodes 2 putative TolC-like proteins; the most downstream is depicted in Fig 4B. (B) Ribbon diagram of trimeric TolC (PDB: 1EK9) from E. coli. One monomer is coloured pink; the other 2 are coloured white. (C) Surface structure of the pink monomer shown in panel B. The transmembrane region of the monomer is coloured using the YRB [68] scale that colours side-chain nitrogens (from R or K residues) red, side-chain oxygens (from D or E residues) blue, and carbon atoms likely to form hydrophobic interactions yellow. The remaining surface structure is coloured grey. (D-E) Phyre2 [48] was used to solve the homology model structure of the TolC-like protein from C. difficile 630. Surface structure of the C. difficile protein modelled against E. coli TolC (PDB: 1EK9) is coloured as in C, with (E) or without (D) a superimposed ribbon diagram of itself. (TIF) [file pbio.3001523.s008.tif]

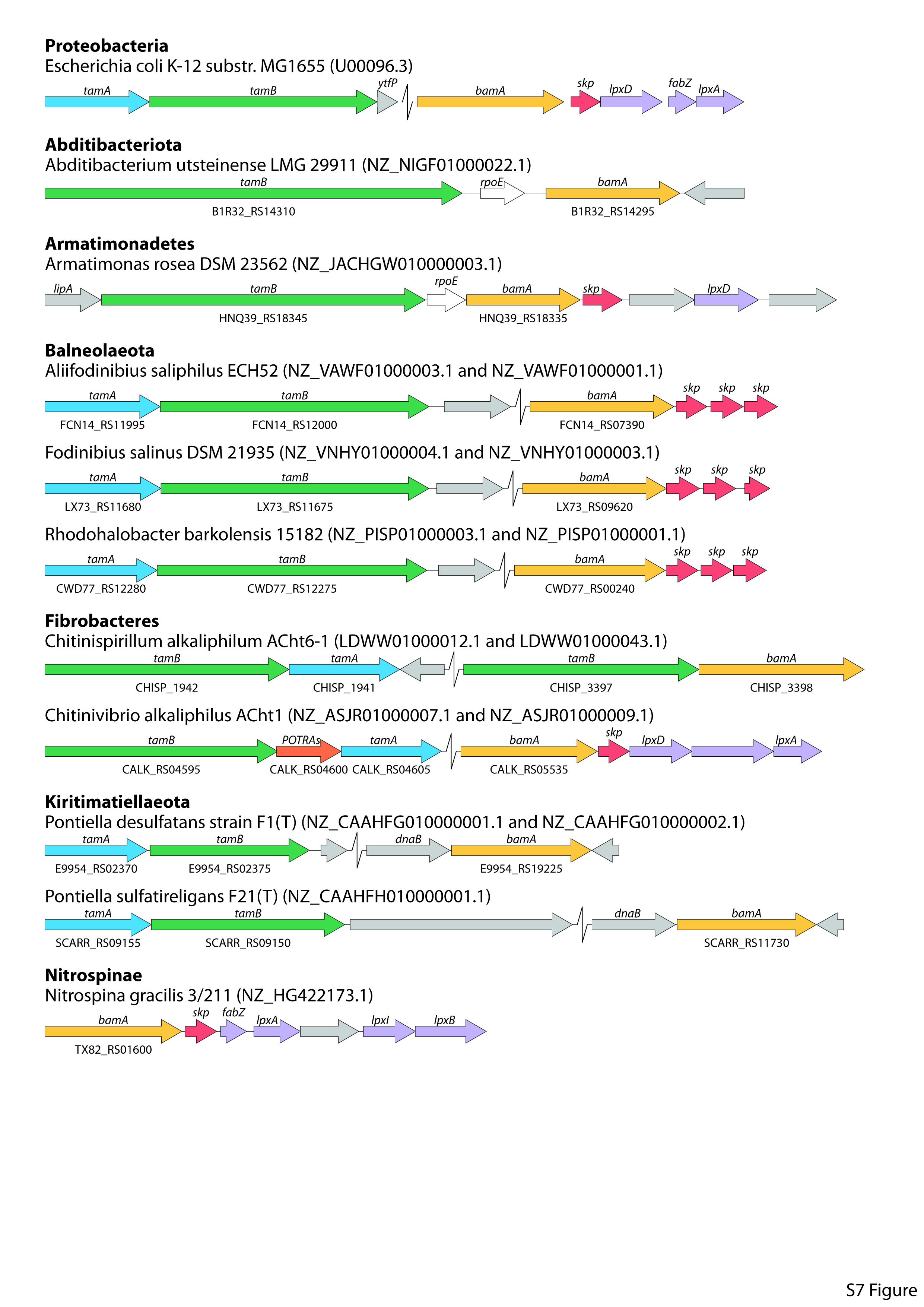

Supplement: S7 Fig — Data underlying Fig 5. The distribution of tamA (blue), tamB (green), and bamA (orange) homologues not previously reported by us for bamA and tamA homologues [20] or for tamB homologues [28]. For comparison, the archetypes from E. coli MG1655 are shown. NCBI accession IDs (and version numbers) are shown alongside strain names. Gene locus tags are displayed beneath the relevant gene of interest. Genes are coloured according to predicted function: red (chaperone); purple (LPS and/or phospholipid synthesis); white (sigma factor); grey (other). It should be noted that, while we previously reported the presence of TamA in the Fibrobacteres Phylum [20], the C. alkaliphilus sequence that has since been published shows a curious case of pseudogenisation of POTRA domains (dark orange) upstream from a putative tamA gene due to a premature stop codon. (TIF) [file pbio.3001523.s009.tif]
