# Supplementary material for: A noncanonical chaperone interacts with drug efflux pumps during their assembly into bacterial outer membranes
Source: PLoS Biol. 2022 Jan 21;20(1):e3001523. doi: 10.1371/journal.pbio.3001523 (PMC8809574; doi:10.1371/journal.pbio.3001523)
Supplement: S1 Raw Images — The uncropped image files of the cropped autoradiographs and immunoblots displayed in the figures and supporting information figures in order of appearance. Apparent sizes in kDa are indicated on the left where applicable. A red box is used to indicate the cropped portion of the image that was displayed in the indicated figure or supporting information figure, which was usually resized to fit within the broader context of each panel. (PDF) [file pbio.3001523.s015.pdf]

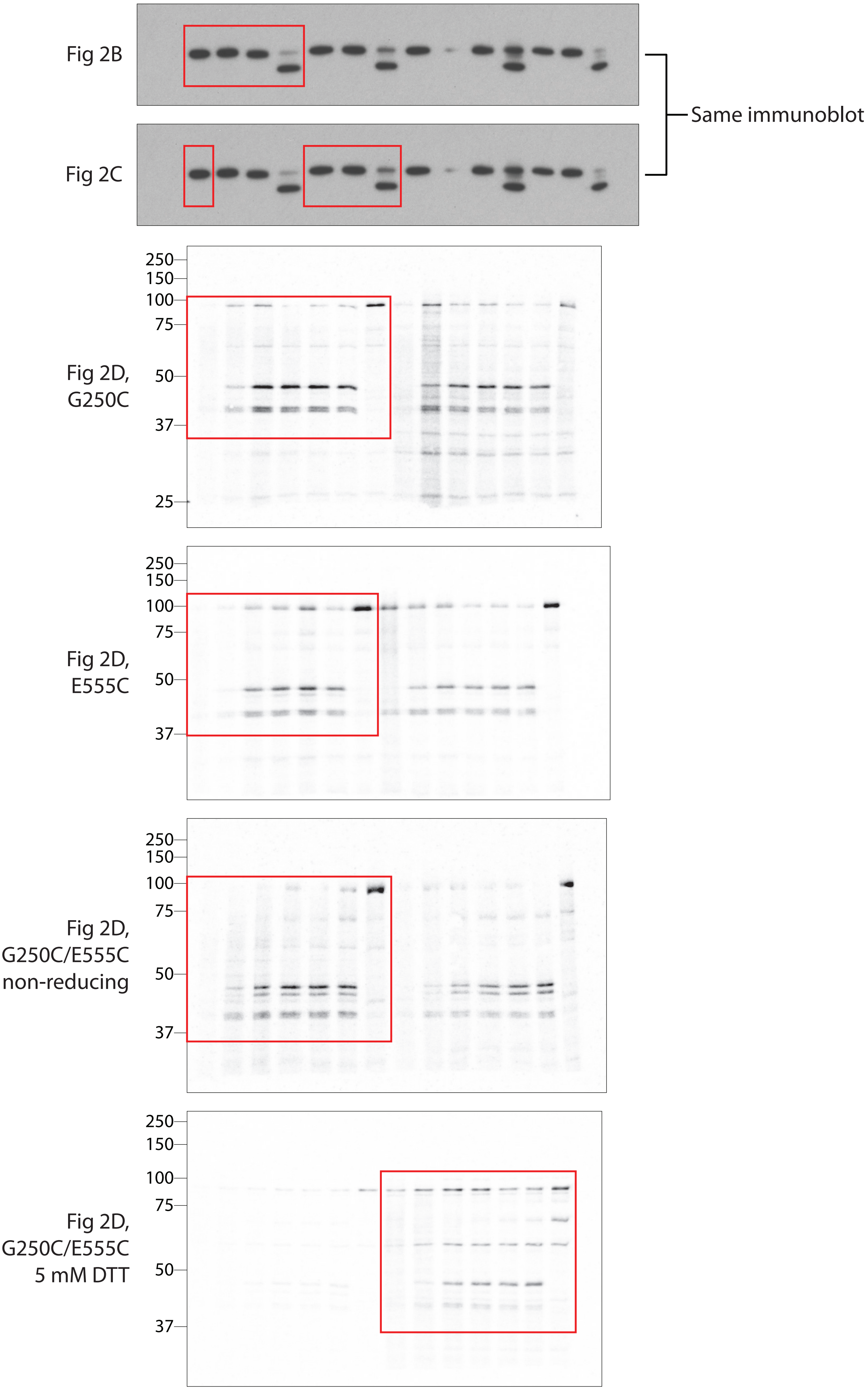
Fig 2B
Fig 2C
Same immunoblot
250
150
100
75
50
37
25
Fig 2D,
G250C
Fig 2D,
E555C
Fig 2D,
G250C/E555C
non-reducing
Fig 2D,
G250C/E555C
5 mM DTT

Fig 2E,
G252C

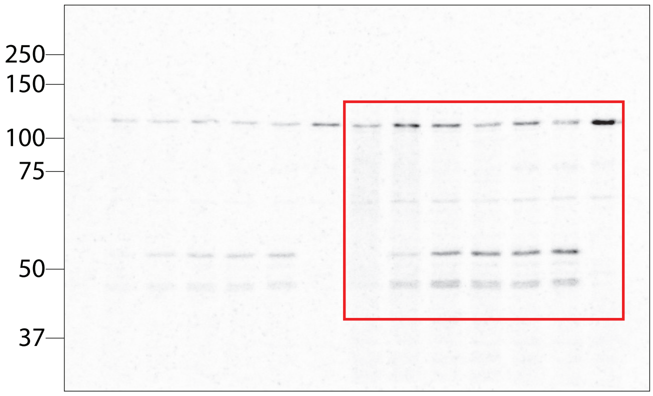

Fig 2E,
G553C

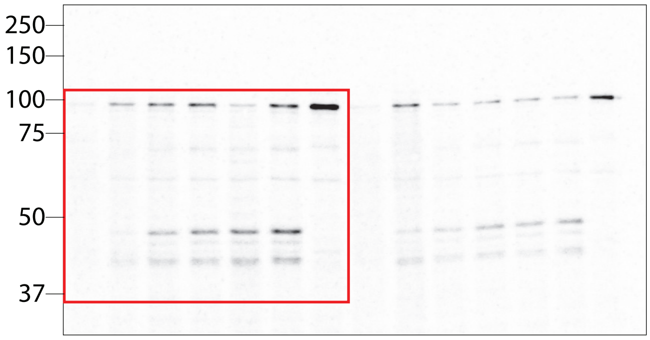

Fig 2E,
G252C/G553C
non-reducing

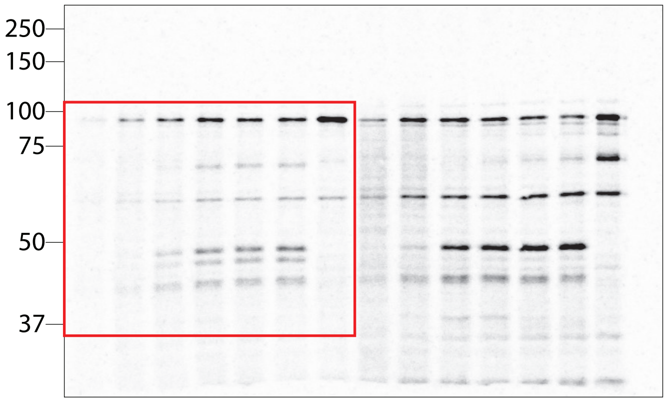

Fig 2E,
G252C/G553C
5 mM DTT

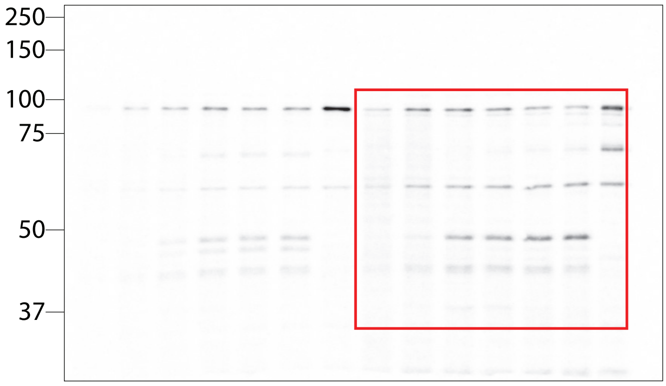

Same autoradiograph

Fig 3C,
top panel
αTamA

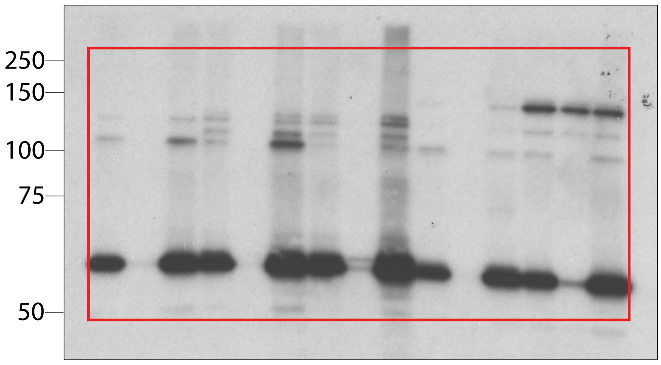

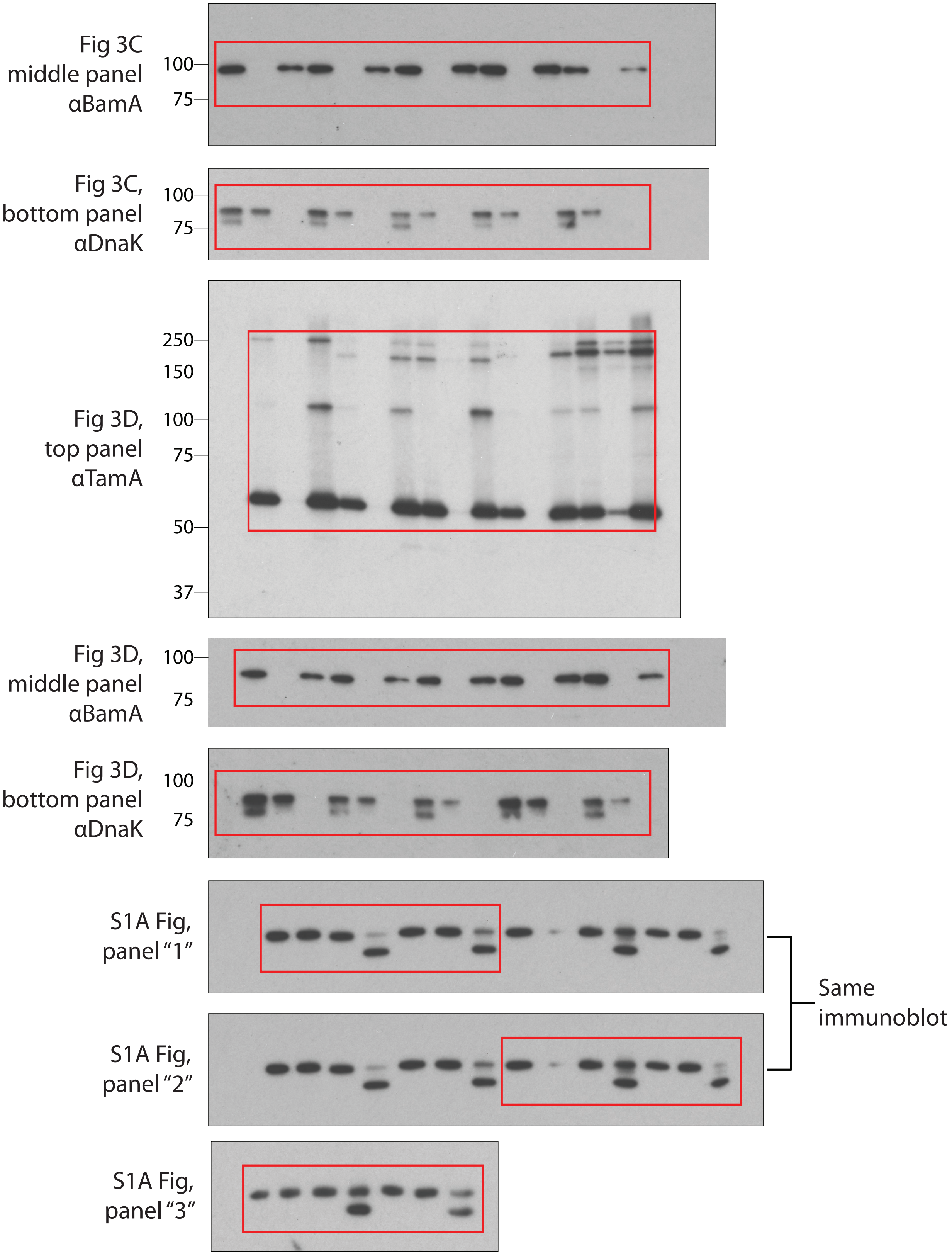
Fig 3C
middle panel
αBamA
100
75
Fig 3C,
bottom panel
αDnaK
100
75
250
150
Fig 3D,
top panel
αTamA
100
75
50
37
Fig 3D,
middle panel
αBamA
100
75
Fig 3D,
bottom panel
αDnaK
100
75
S1A Fig,
panel "1"
S1A Fig,
panel "2"
Same
immunoblot
S1A Fig,
panel "3"

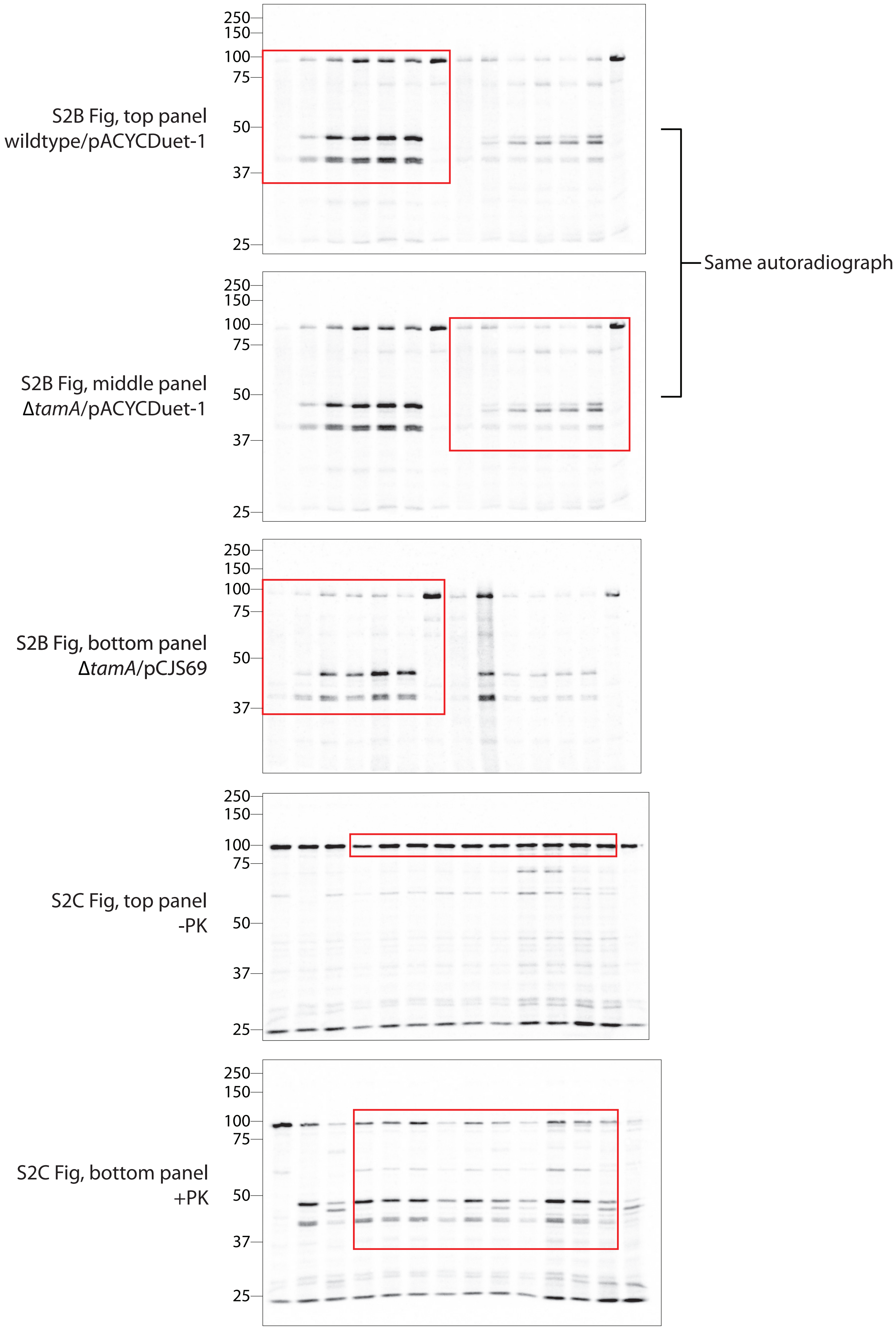
S2B Fig, top panel
wildtype/pACYCDuet-1
S2B Fig, middle panel
ΔtamA/pACYCDuet-1
Same autoradiograph
S2B Fig, bottom panel
ΔtamA/pCJS69
S2C Fig, top panel
-PK
S2C Fig, bottom panel
+PK
250
150
100
75
50
37
25

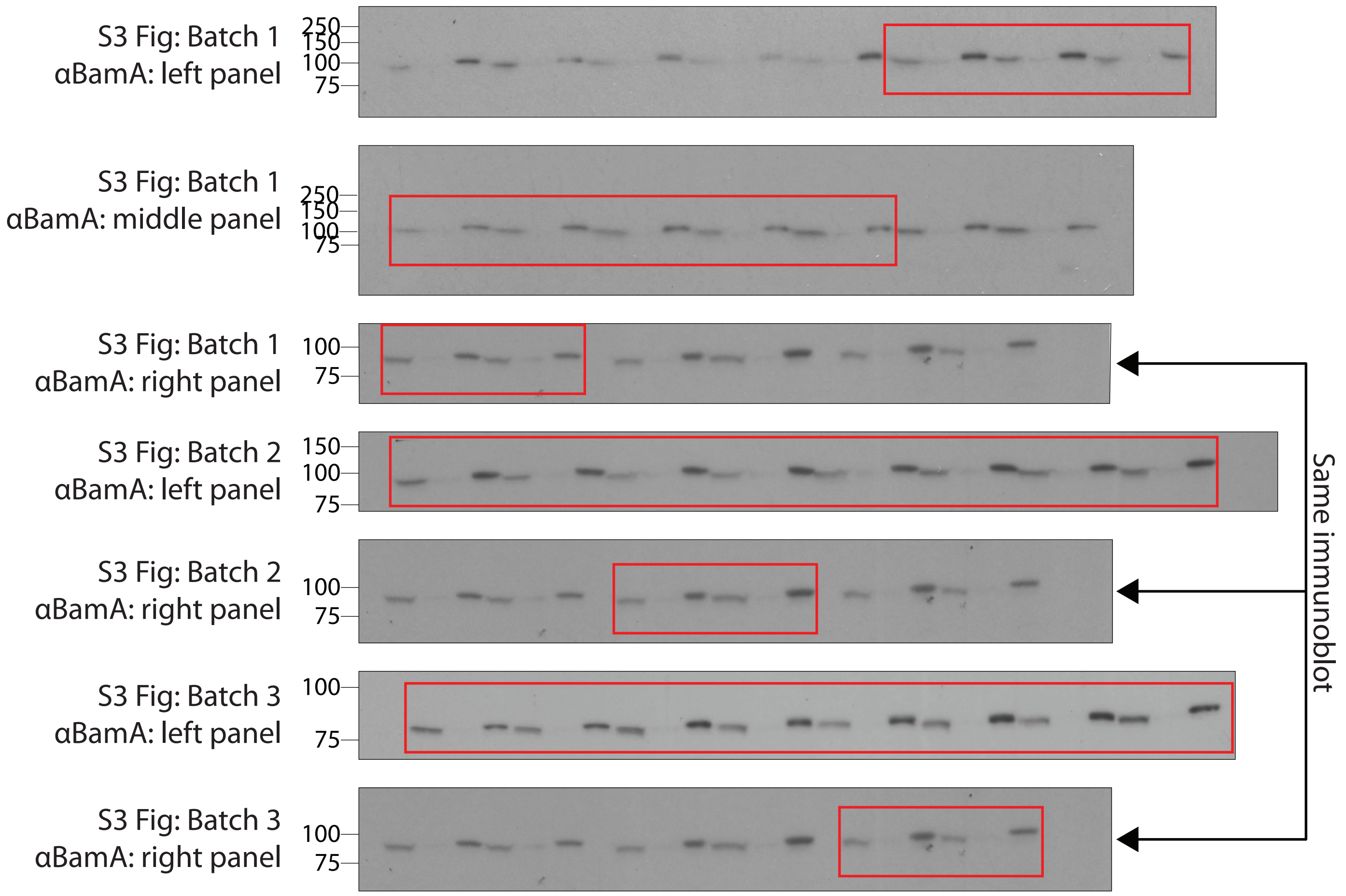
S3 Fig: Batch 1
αBamA: left panel
250
150
100
75
S3 Fig: Batch 1
αBamA: middle panel
250
150
100
75
S3 Fig: Batch 1
αBamA: right panel
100
75
S3 Fig: Batch 2
αBamA: left panel
150
100
75
S3 Fig: Batch 2
αBamA: right panel
100
75
S3 Fig: Batch 3
αBamA: left panel
100
75
S3 Fig: Batch 3
αBamA: right panel
100
75
Same immunoblot

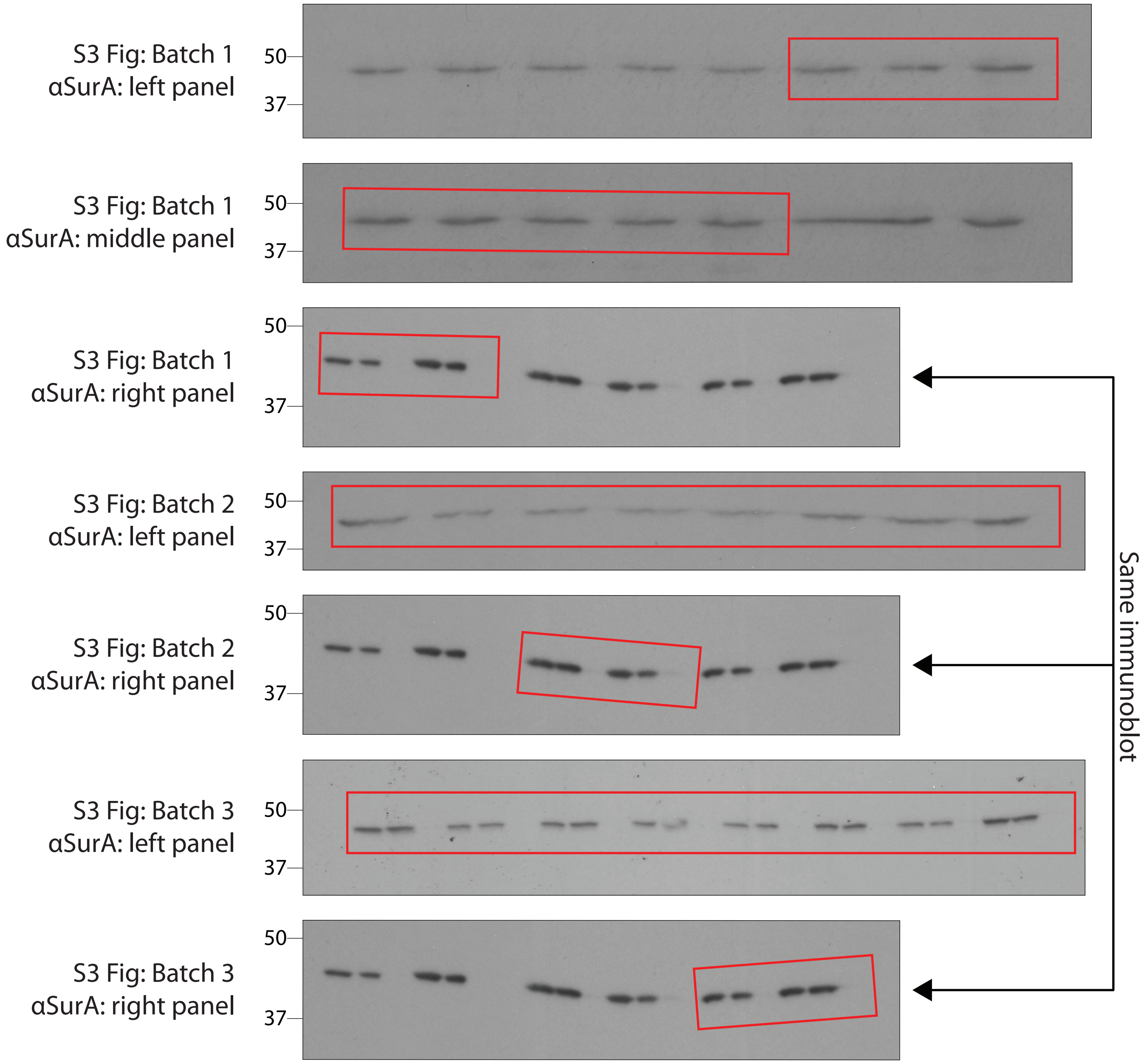
S3 Fig: Batch 1
αSurA: left panel
S3 Fig: Batch 1
αSurA: middle panel
S3 Fig: Batch 1
αSurA: right panel
S3 Fig: Batch 2
αSurA: left panel
S3 Fig: Batch 2
αSurA: right panel
S3 Fig: Batch 3
αSurA: left panel
S3 Fig: Batch 3
αSurA: right panel
50
37
Same immunoblot

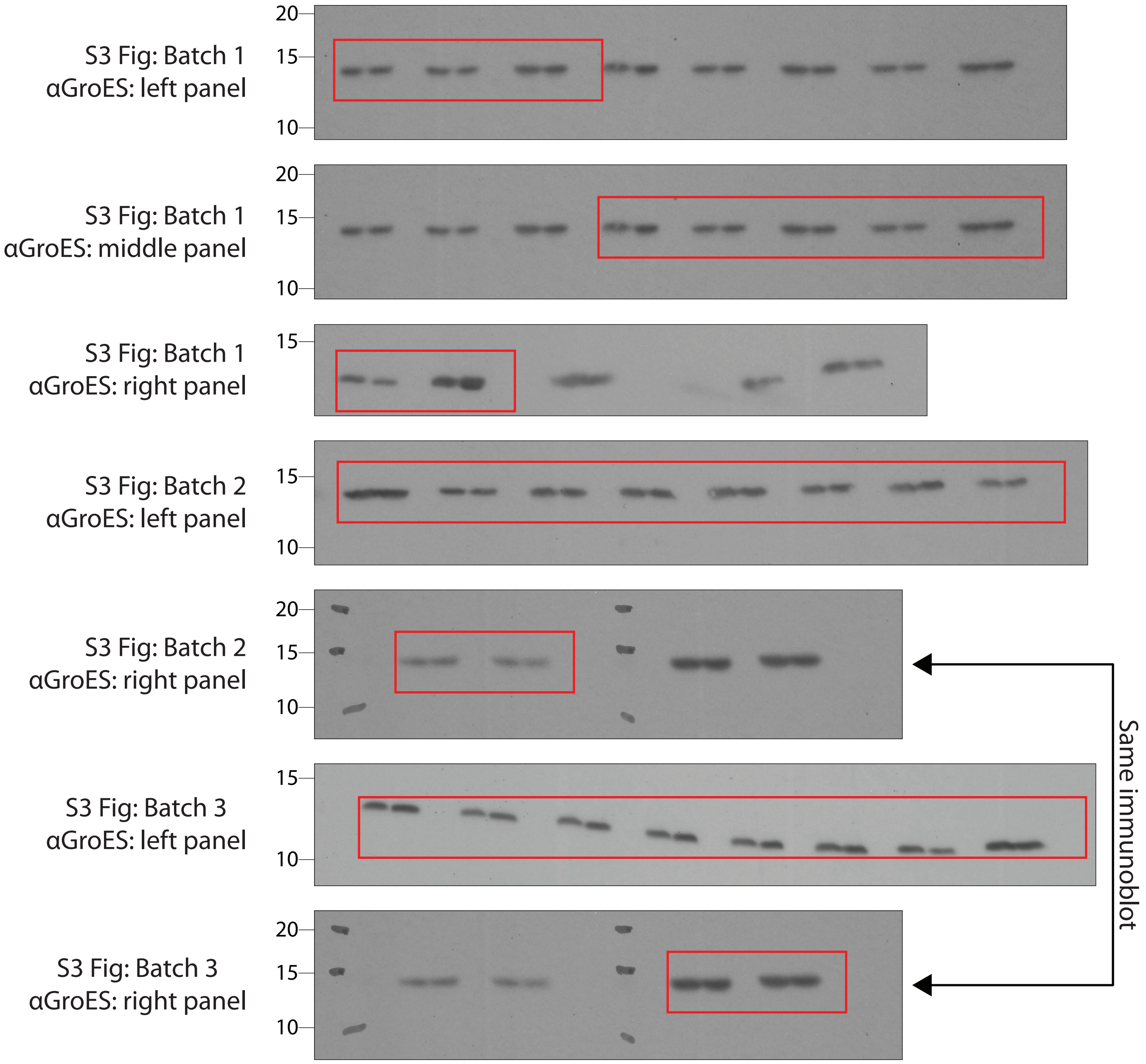
S3 Fig: Batch 1
αGroES: left panel
S3 Fig: Batch 1
αGroES: middle panel
S3 Fig: Batch 1
αGroES: right panel
S3 Fig: Batch 2
αGroES: left panel
S3 Fig: Batch 2
αGroES: right panel
S3 Fig: Batch 3
αGroES: left panel
S3 Fig: Batch 3
αGroES: right panel
Same immunoblot
20
15
10

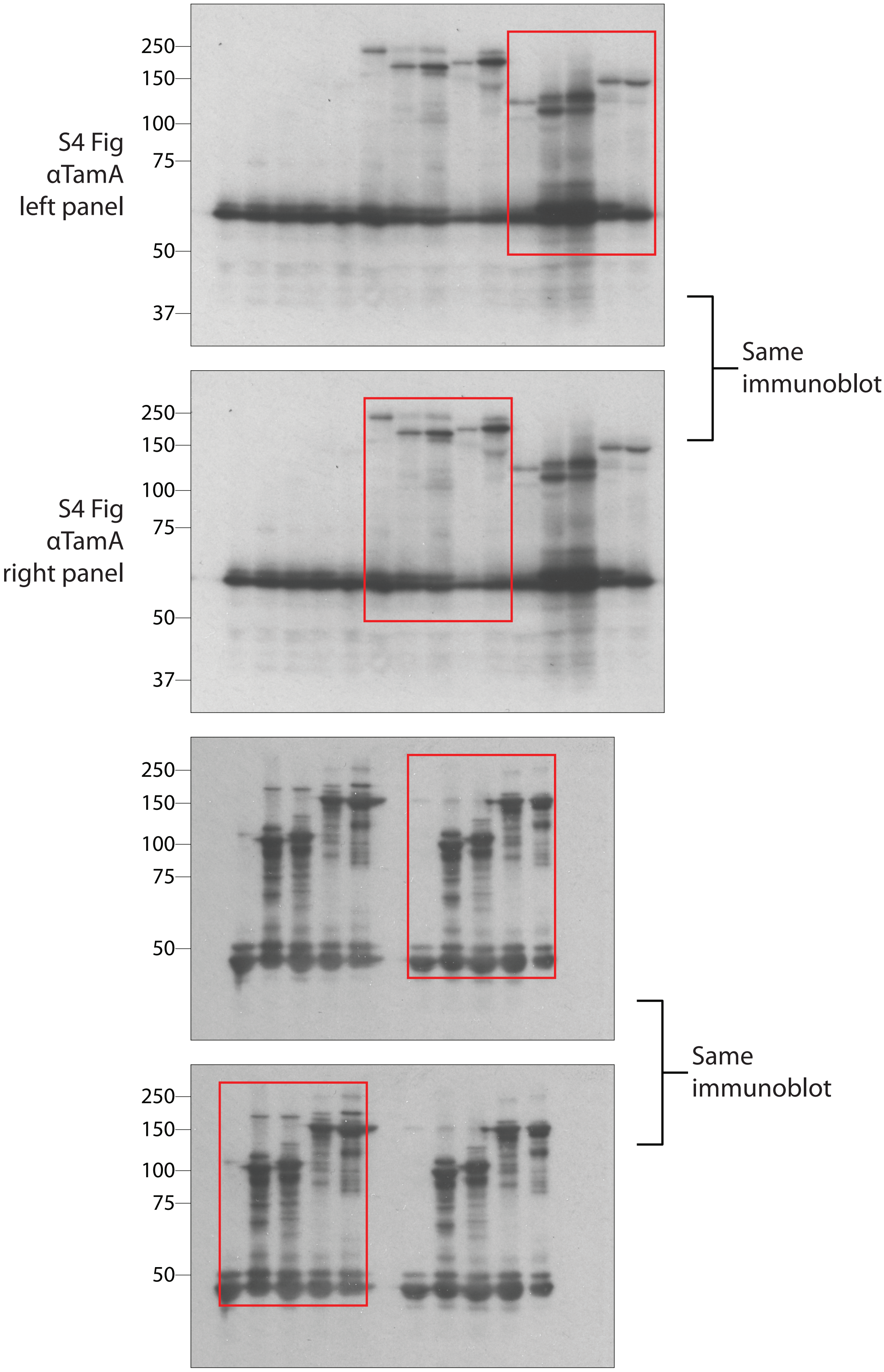
S4 Fig
αTamA
left panel
S4 Fig
αTamA
right panel
Same immunoblot
Same immunoblot
250
150
100
75
50
37

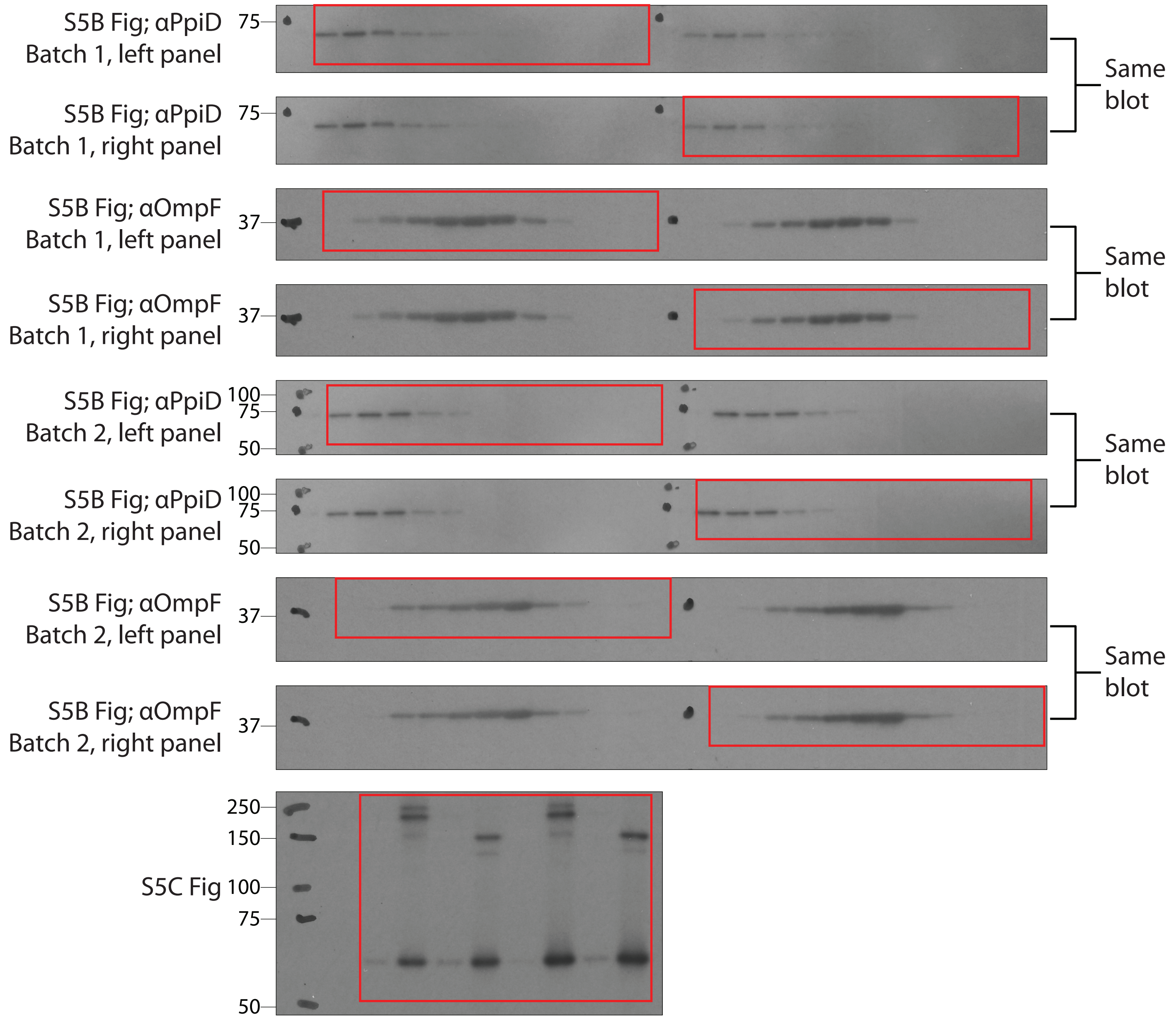
S5B Fig; αPpiD
Batch 1, left panel
75
S5B Fig; αPpiD
Batch 1, right panel
75
Same blot
S5B Fig; αOmpF
Batch 1, left panel
37
S5B Fig; αOmpF
Batch 1, right panel
37
Same blot
S5B Fig; αPpiD
Batch 2, left panel
100
75
50
S5B Fig; αPpiD
Batch 2, right panel
100
75
50
Same blot
S5B Fig; αOmpF
Batch 2, left panel
37
S5B Fig; αOmpF
Batch 2, right panel
37
Same blot
S5C Fig
250
150
100
75
50
