# Supplementary material for: A noncanonical chaperone interacts with drug efflux pumps during their assembly into bacterial outer membranes
Source: PLoS Biol. 2022 Jan 21;20(1):e3001523. doi: 10.1371/journal.pbio.3001523 (PMC8809574; doi:10.1371/journal.pbio.3001523)
Supplement: S2 Raw Images — FimD assembly was monitored over time by pulse chase analysis using E. coli BL21 Star (DE3) ΔtamA cells complemented with the indicated TamA cysteine mutants (on a pACYCDuet-1 vector backbone) and also harbouring pKS02 (the fimD expression vector). In total, 28 gels are depicted and annotated based on the complementing TamA cysteine mutant indicated at the top of the page. For gels #1, #5, #8, #11, #13, and #19, aliquots were taken at 10 s, 20 s, and every 20 s thereafter for a total of 180 s and treated with (+) or without (−) proteinase K (PK) as indicated. For gels #23–28, aliquots were taken at 8 min and treated with protease as indicated. Otherwise (for the remaining gels), aliquots were taken at 10 s (0 min), 2, 4, 8, 16, and 32 min and treated with (+) or without (−) proteinase K (PK) as indicated. Total protein was analysed by 8% SDS-PAGE (for gels #4, #10, #16–18, #20–22) or 12% SDS-PAGE (for the remaining gels) and storage phosphor imaging, where radiation was captured using Amersham Typhoon 5 Biomolecular Imager. Due to the nature of the imager, the “raw” gel file pixel intensity is relative to the most saturated peak scanned across the entire image, and in most cases, the raw gel appears very faint (and cannot be annotated by itself). Therefore, an “adjusted” gel file that could be annotated (i.e., level adjustment) appears alongside the “raw” gel file. Gels represent biological replicates, except gels #16 and #17, which include technical replicates for G250C/E555C nonreducing conditions. Gels that have been used in the main or supporting information figures are indicated with red text. (PDF) [file pbio.3001523.s016.pdf]

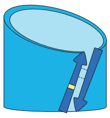

G250C non-reducing
4/4 biological replicates

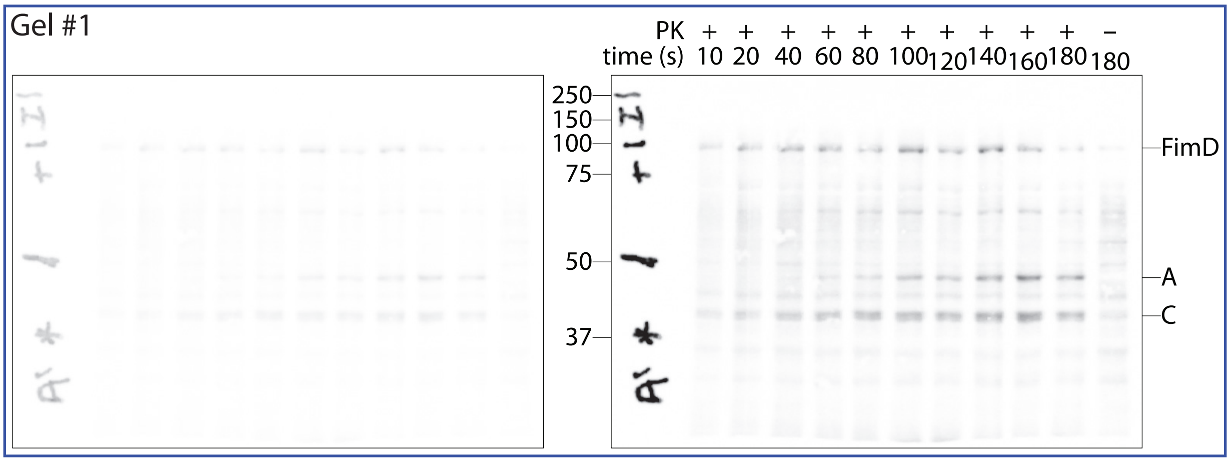

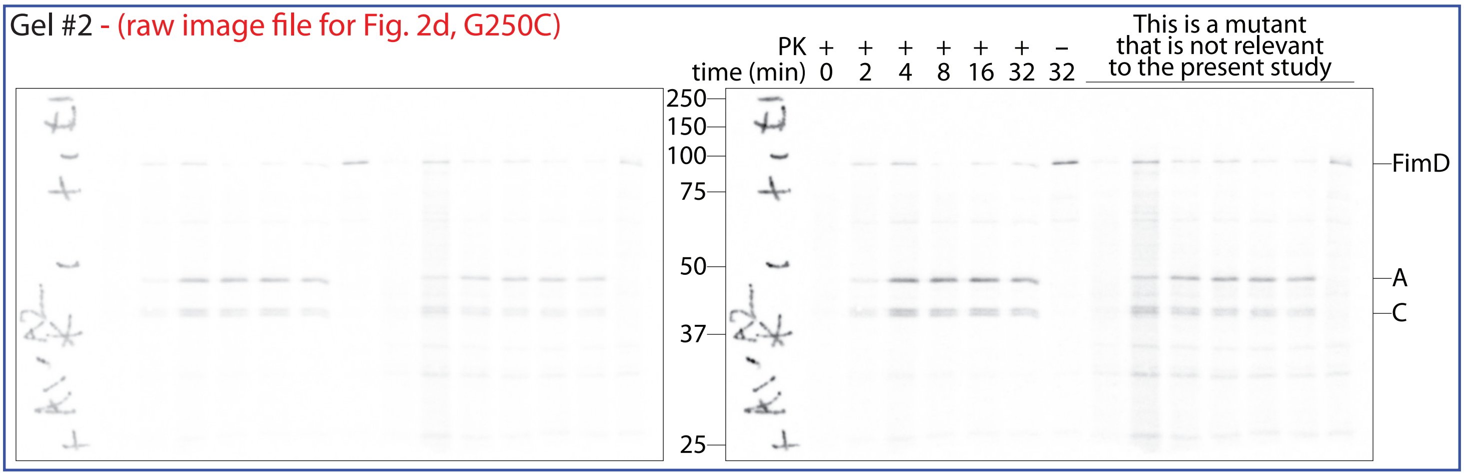

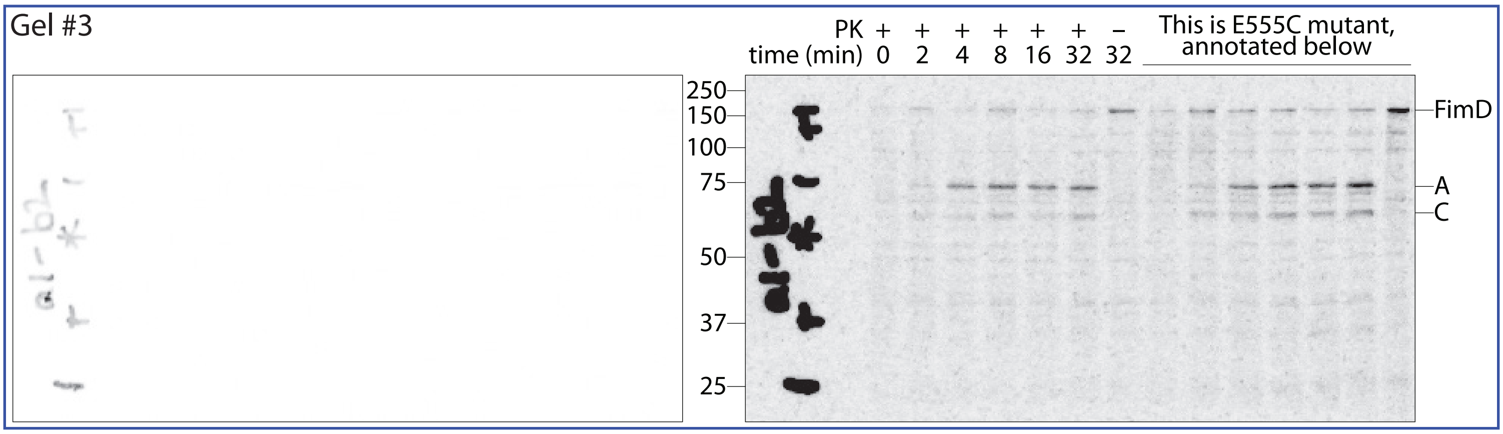

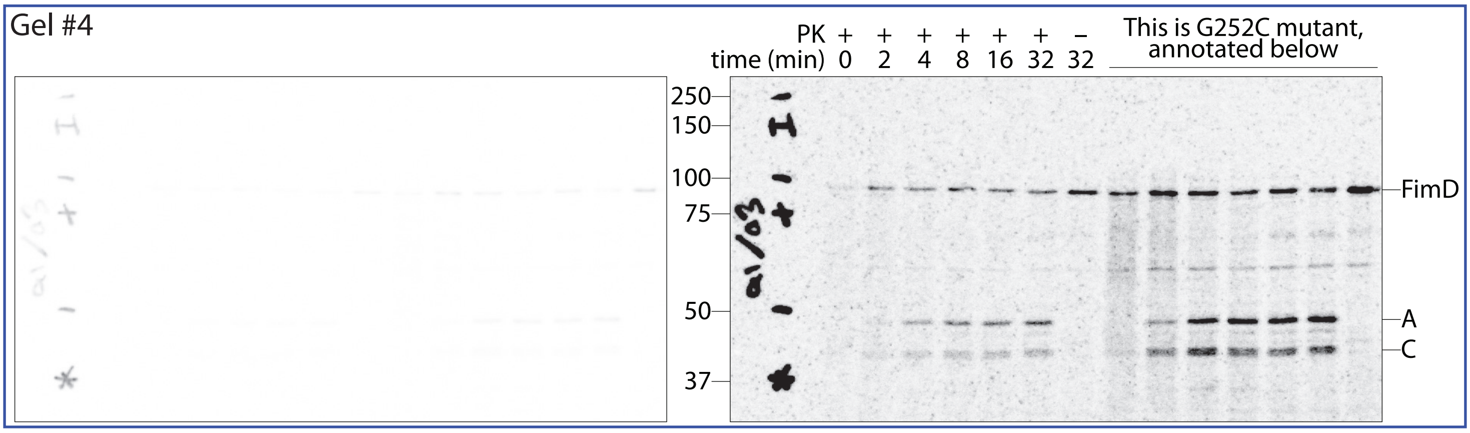

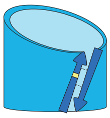

G252C non-reducing
4/4 biological replicates

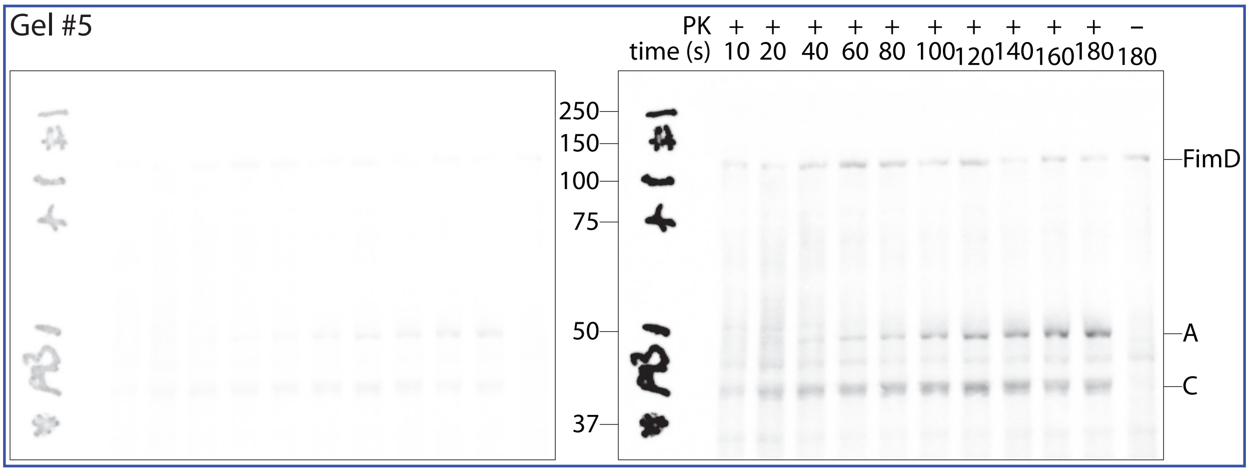

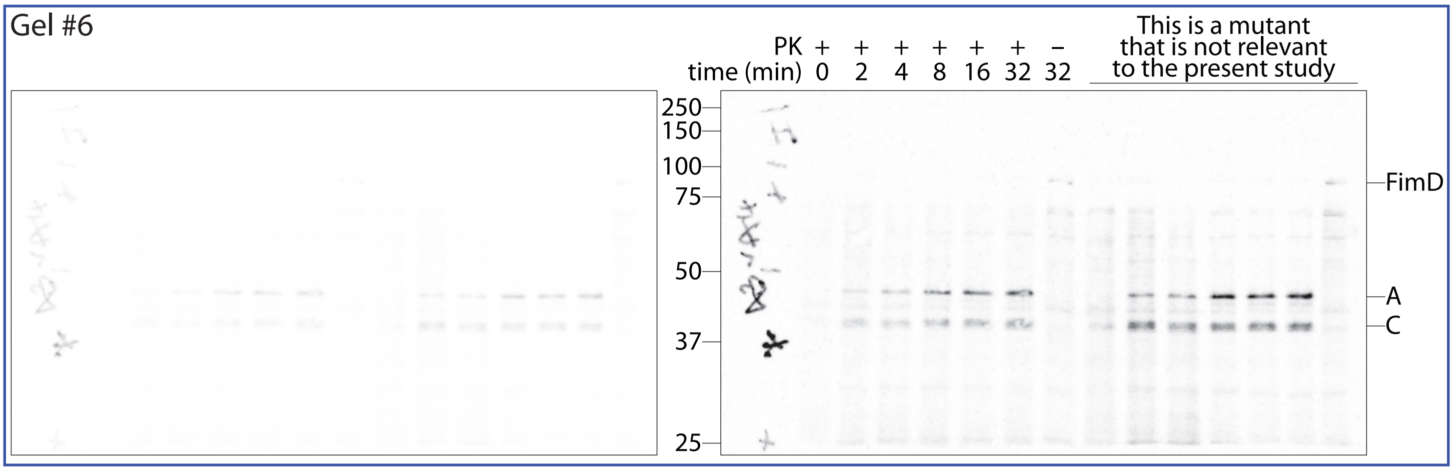

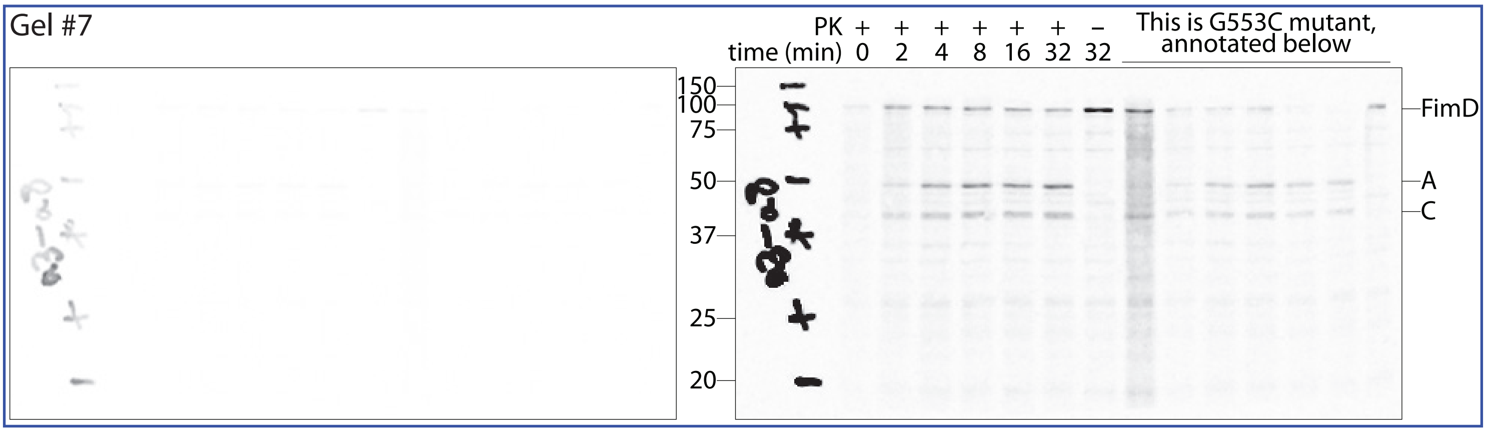

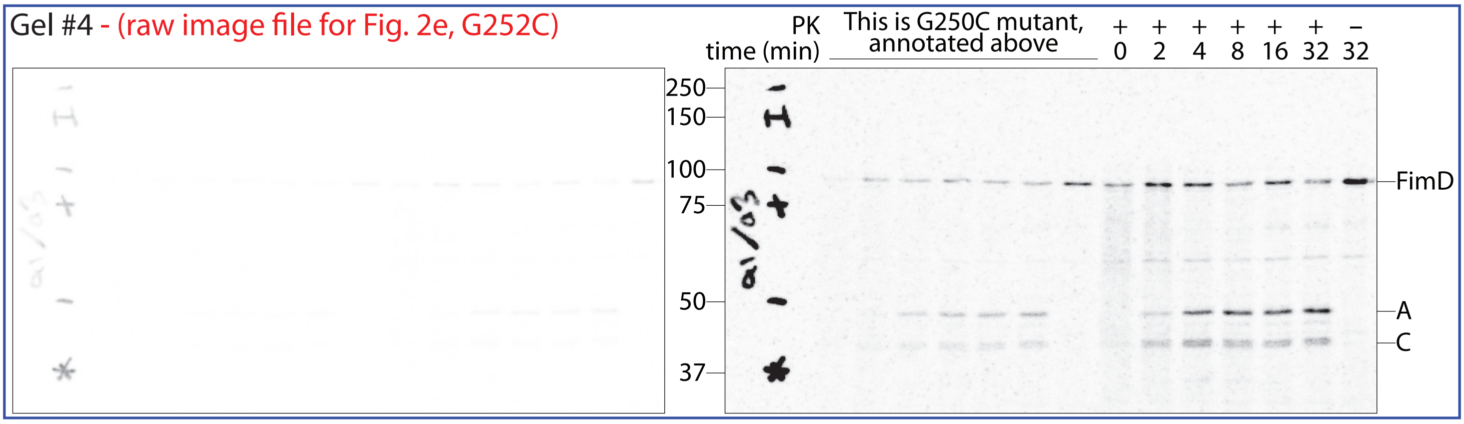

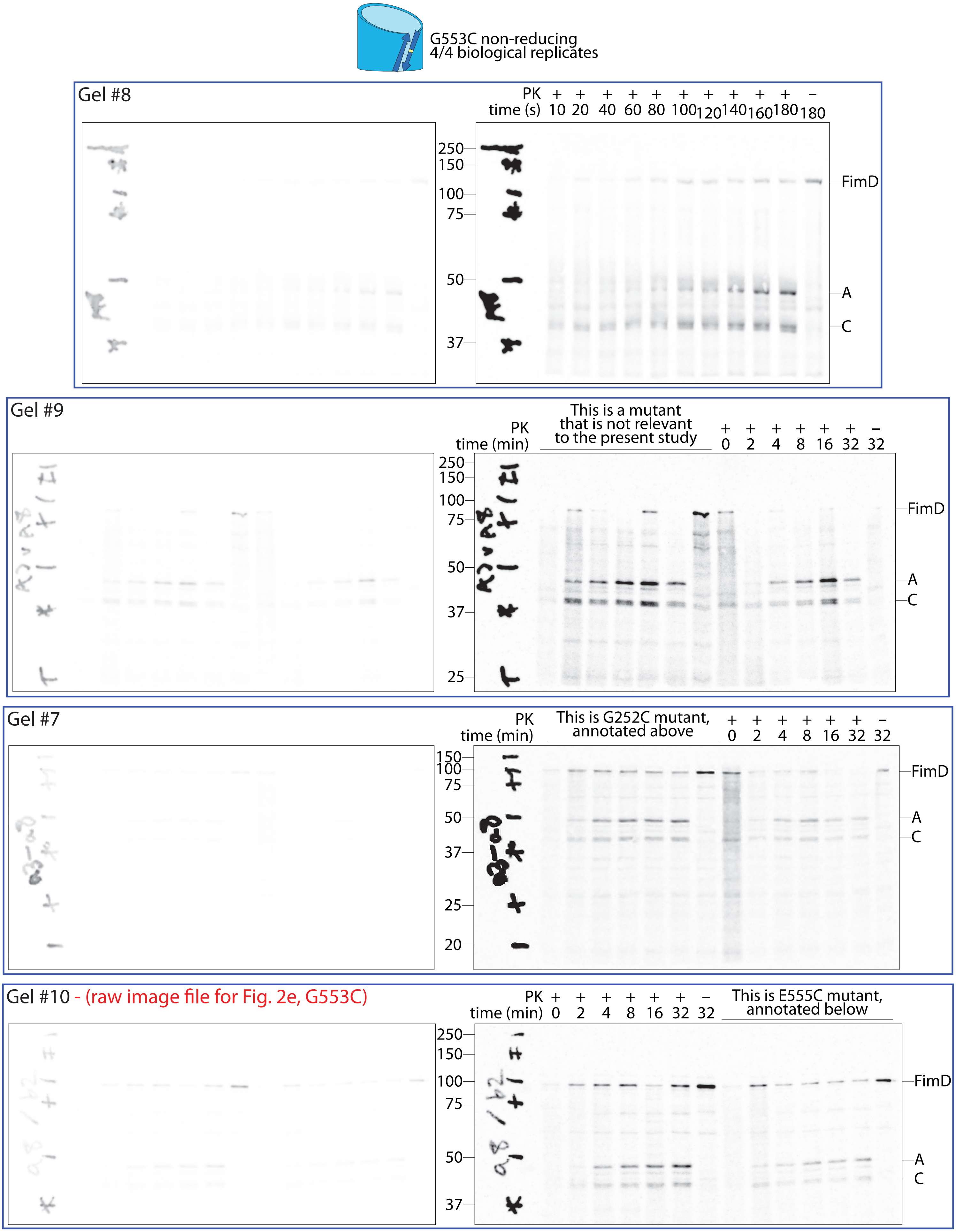
G553C non-reducing
4/4 biological replicates
Gel #8
PK + + + + + + + + + + –
time (s) 10 20 40 60 80 100 120 140 160 180 180
250
150
100
75
50
37
FimD
A
C
Gel #9
This is a mutant
that is not relevant
to the present study
PK + + + + + + –
time (min) 0 2 4 8 16 32 32
250
150
100
75
50
37
25
FimD
A
C
Gel #7
This is G252C mutant,
annotated above
PK + + + + + + –
time (min) 0 2 4 8 16 32 32
150
100
75
50
37
25
20
FimD
A
C
Gel #10 - (raw image file for Fig. 2e, G553C)
PK + + + + + + –
time (min) 0 2 4 8 16 32 32
This is E555C mutant,
annotated below
250
150
100
75
50
37
FimD
A
C

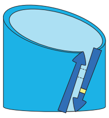

E555C non-reducing
4/4 biological replicates

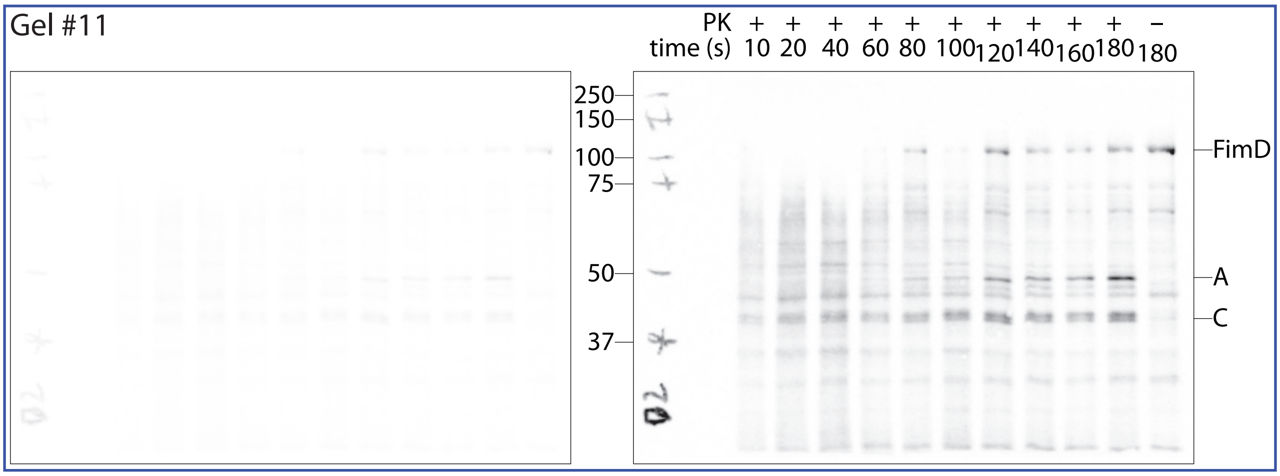

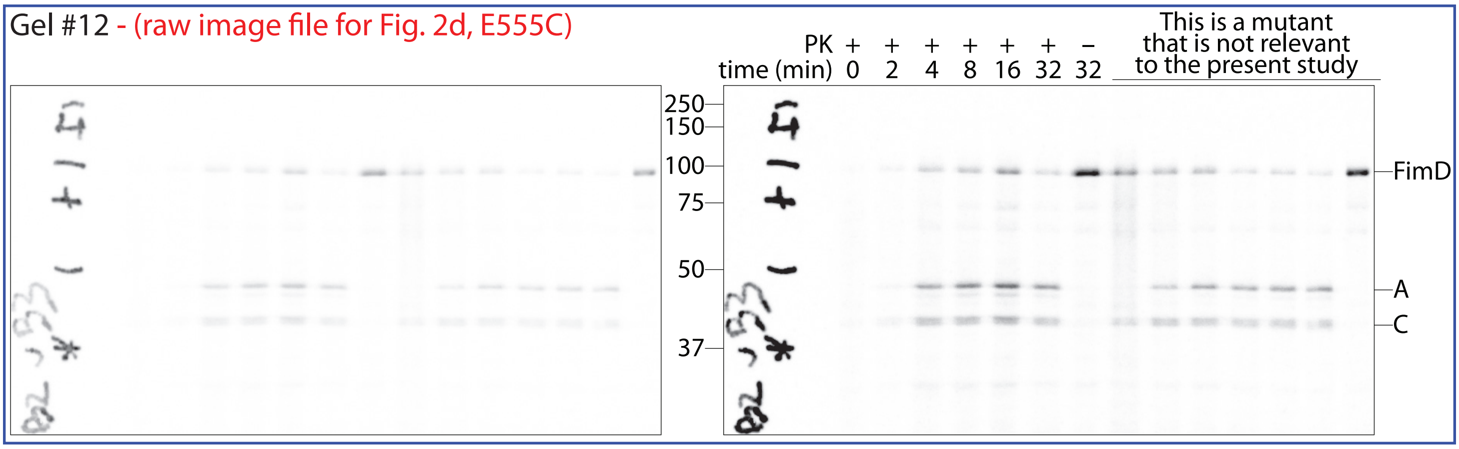

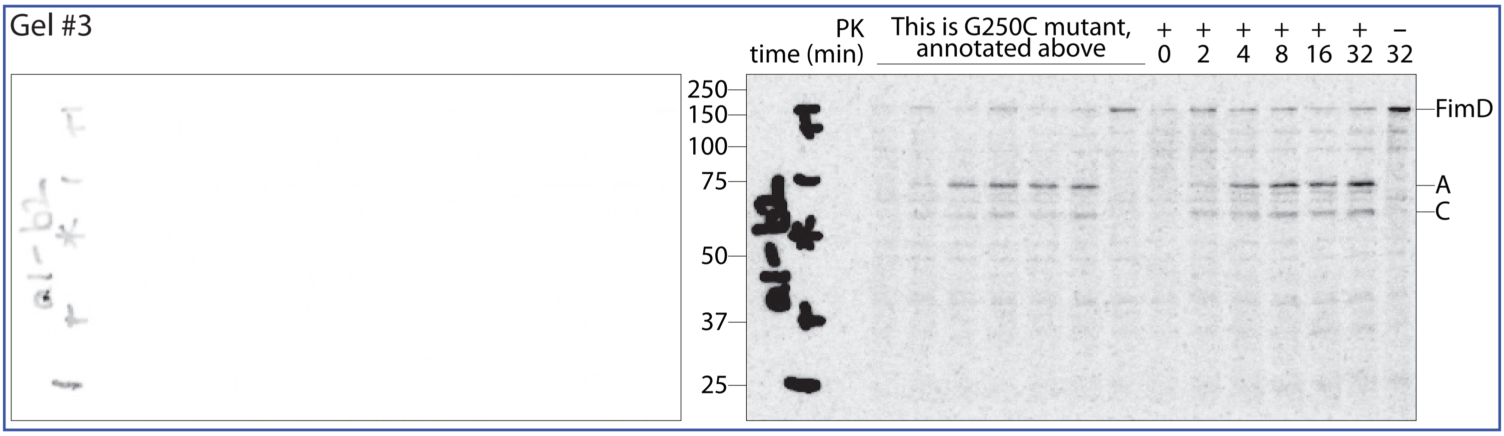

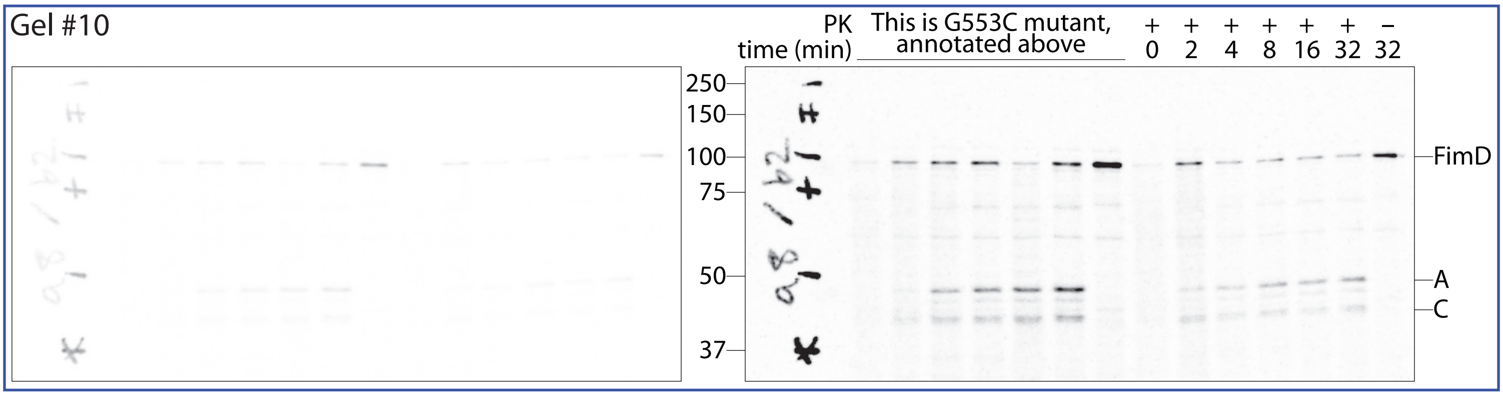

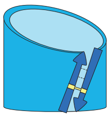

G250C/E555C non-reducing
3/5 biological replicates (total: 3/5 biological replicates)

Gel #13

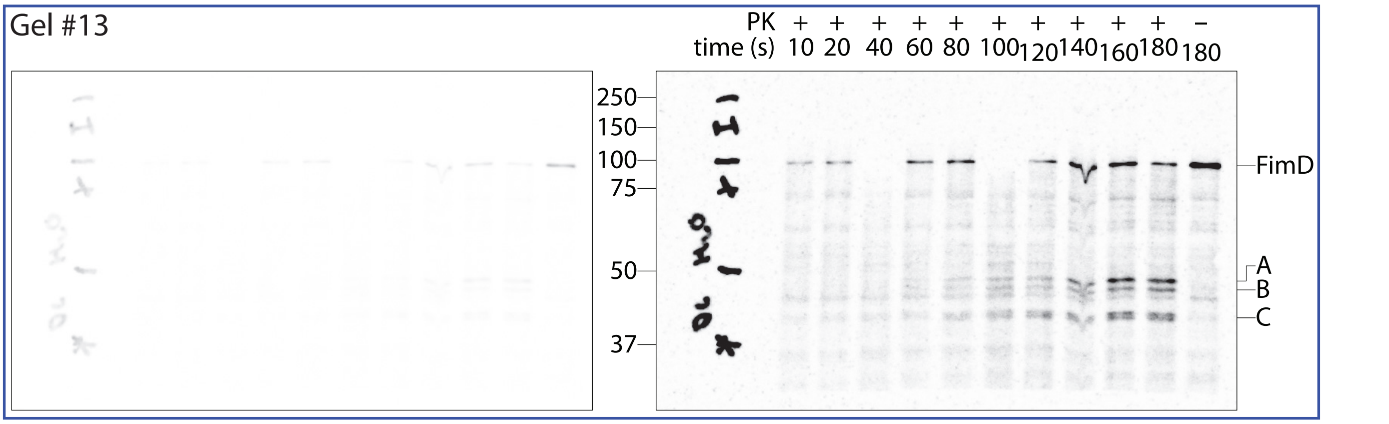

Gel #14 - (raw image file for Fig. 2d, G250C/E555C non-reducing)

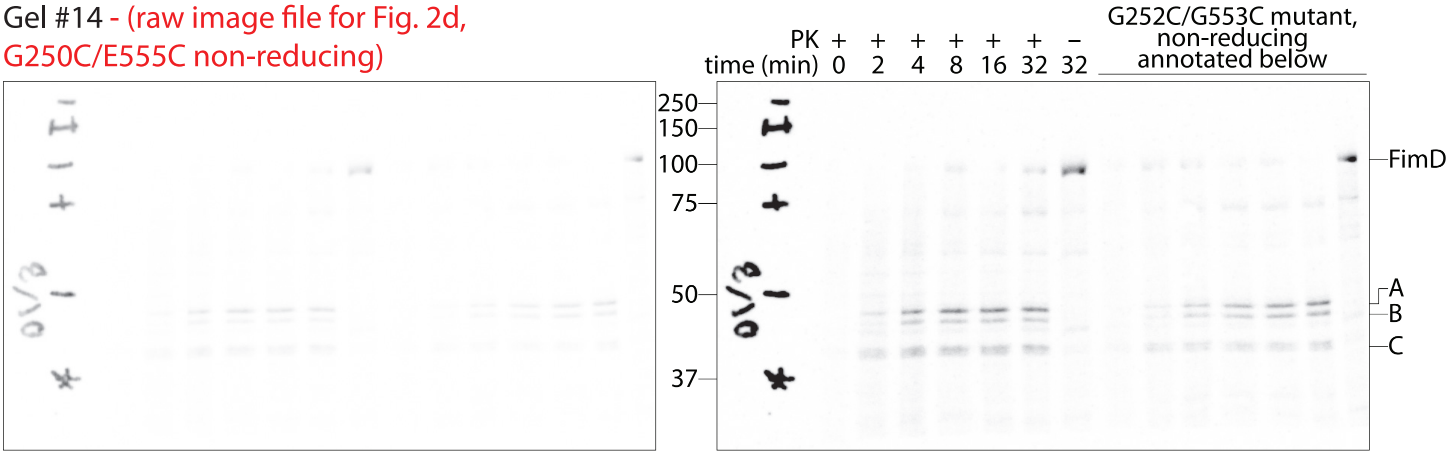

Gel #15

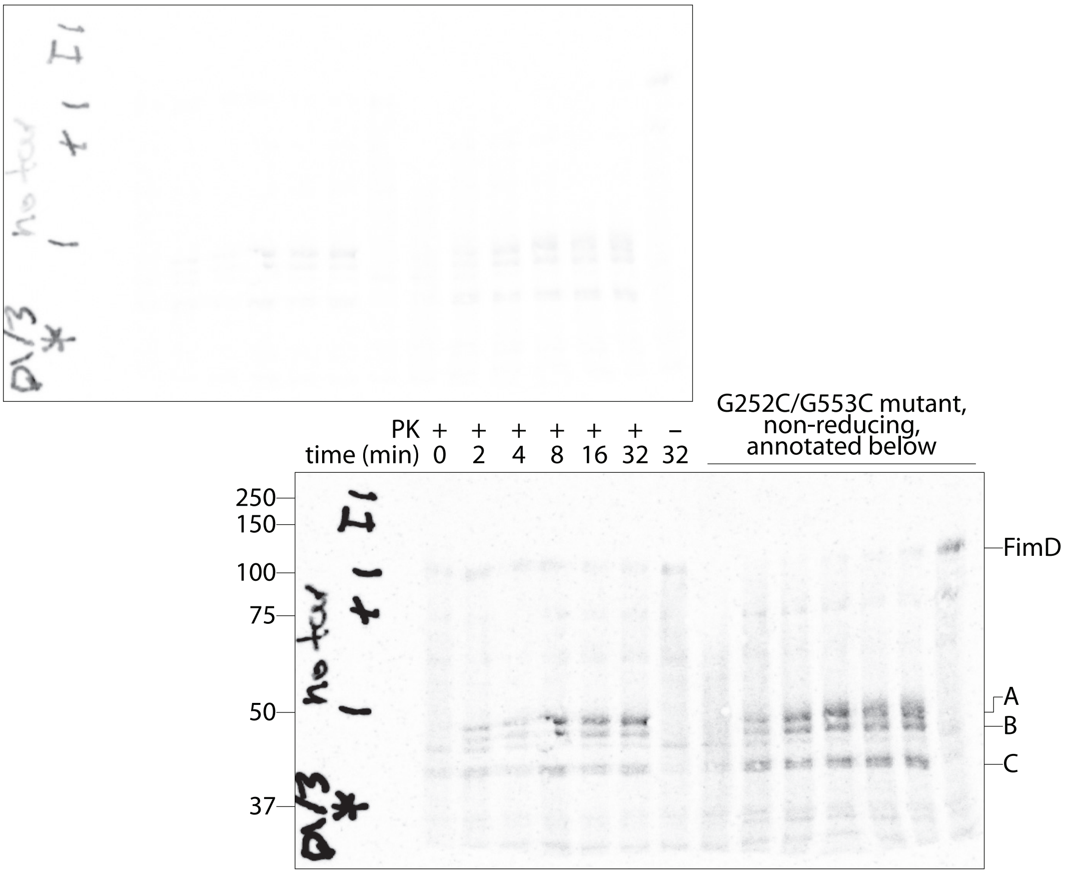

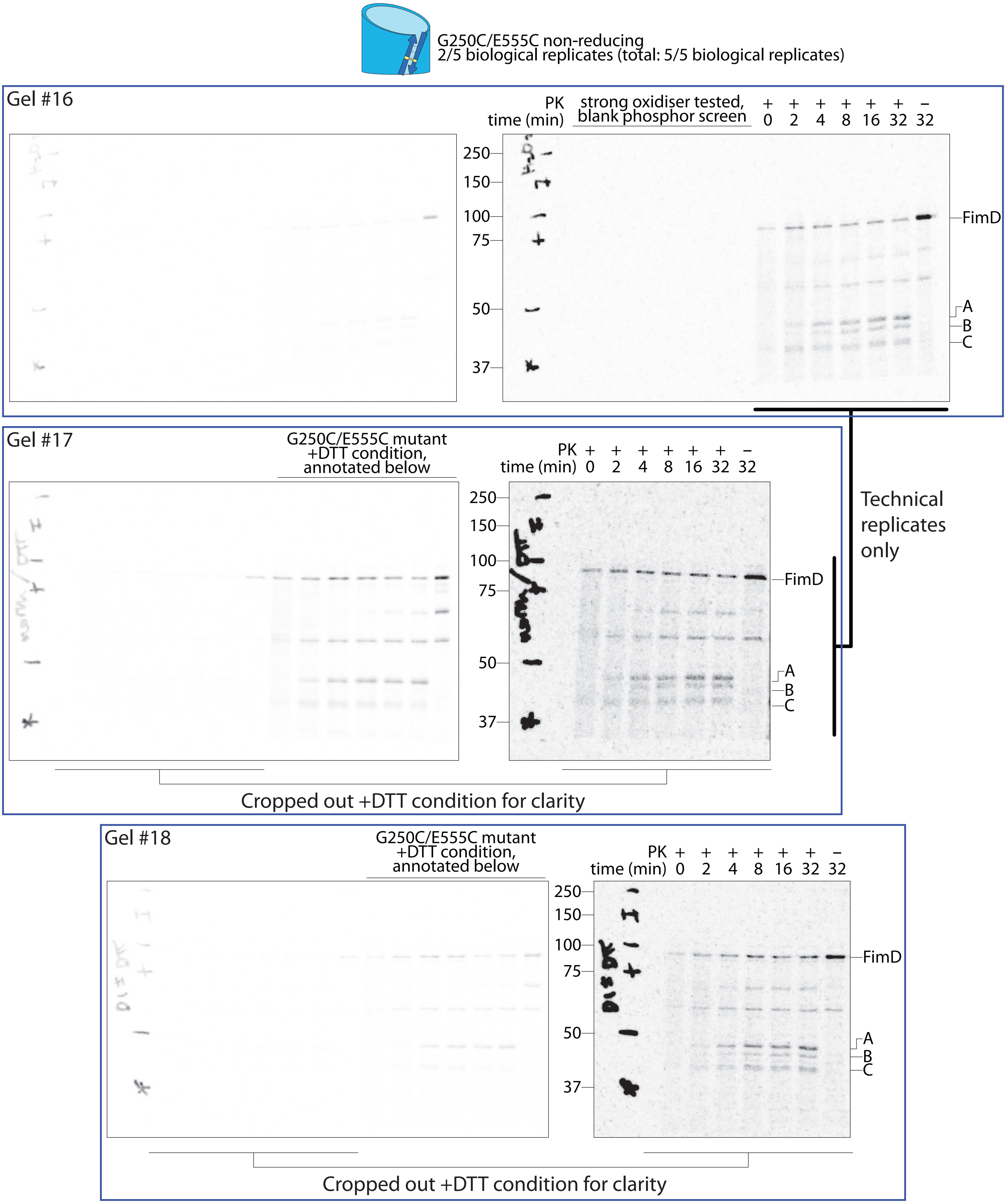
G250C/E555C non-reducing
2/5 biological replicates (total: 5/5 biological replicates)
Gel #16
PK + + + + + + −
time (min) 0 2 4 8 16 32 32
strong oxidiser tested, blank phosphor screen
250
150
100
75
50
37
FimD
A
B
C
Technical replicates only
Gel #17
G250C/E555C mutant +DTT condition, annotated below
PK + + + + + + −
time (min) 0 2 4 8 16 32 32
250
150
100
75
50
37
FimD
A
B
C
Cropped out +DTT condition for clarity
Gel #18
G250C/E555C mutant +DTT condition, annotated below
PK + + + + + + −
time (min) 0 2 4 8 16 32 32
250
150
100
75
50
37
FimD
A
B
C
Cropped out +DTT condition for clarity

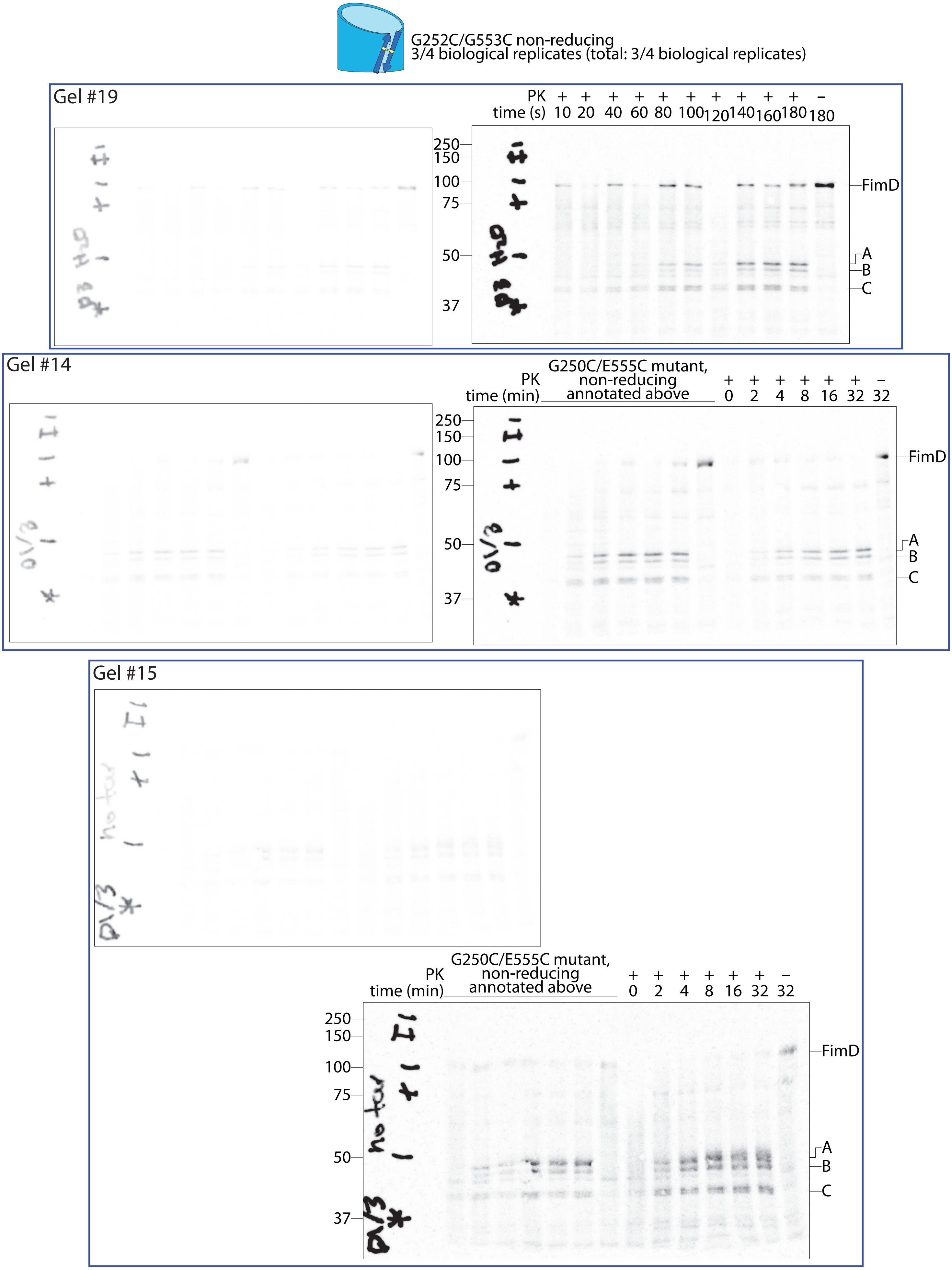

G252C/G553C non-reducing
3/4 biological replicates (total: 3/4 biological replicates)
Gel #19
PK + + + + + + + + + + –
time (s) 10 20 40 60 80 100 120 140 160 180 180
250
150
100
75
50
37
FimD
A
B
C
Gel #14
PK
time (min)
G250C/E555C mutant, non-reducing annotated above
+ + + + + + –
0 2 4 8 16 32 32
250
150
100
75
50
37
FimD
A
B
C
Gel #15
PK
time (min)
G250C/E555C mutant, non-reducing annotated above
+ + + + + + –
0 2 4 8 16 32 32
250
150
100
75
50
37
FimD
A
B
C

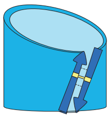

G252C/G553C non-reducing
1/4 biological replicates (total: 4/4 biological replicates)

Gel #20 - (raw image file for Fig. 2e, G252C/G553C non-reducing)

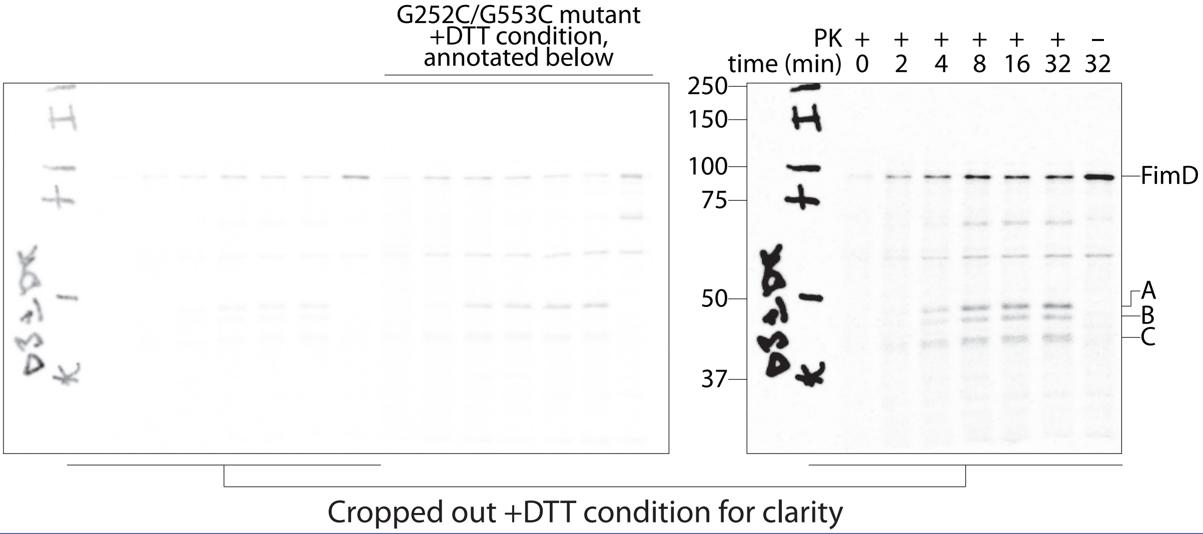

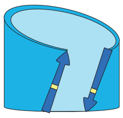

G250C/E555C with 5 mM DTT
4/4 biological replicates

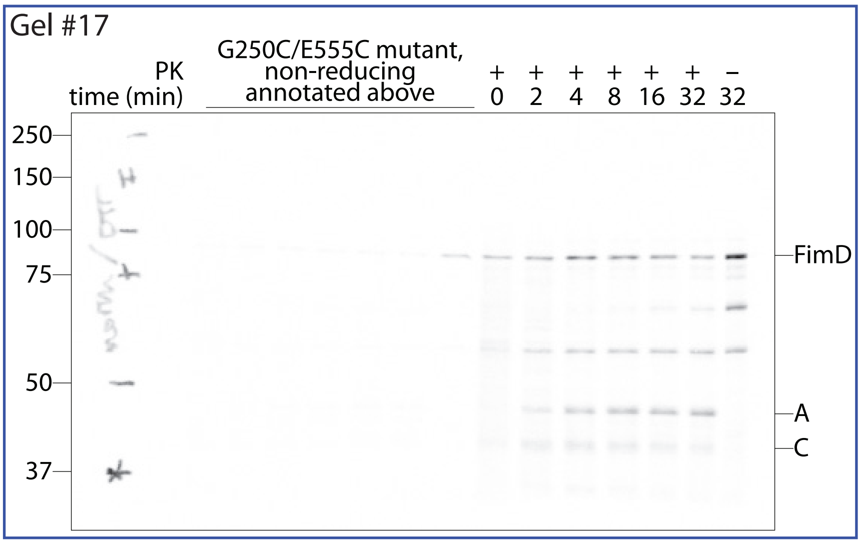

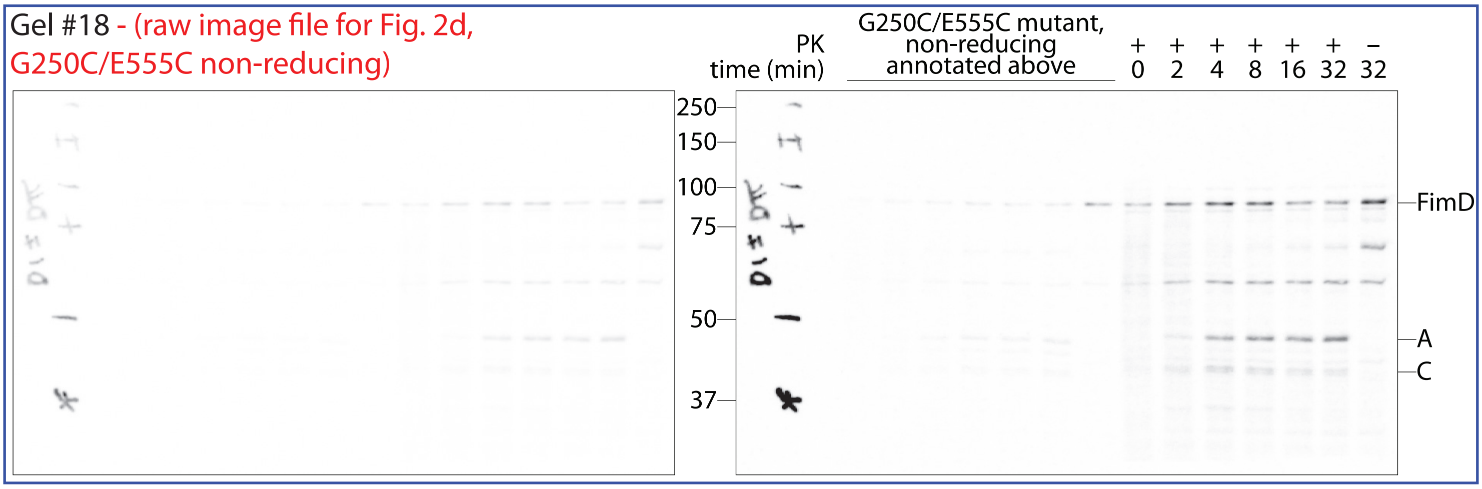

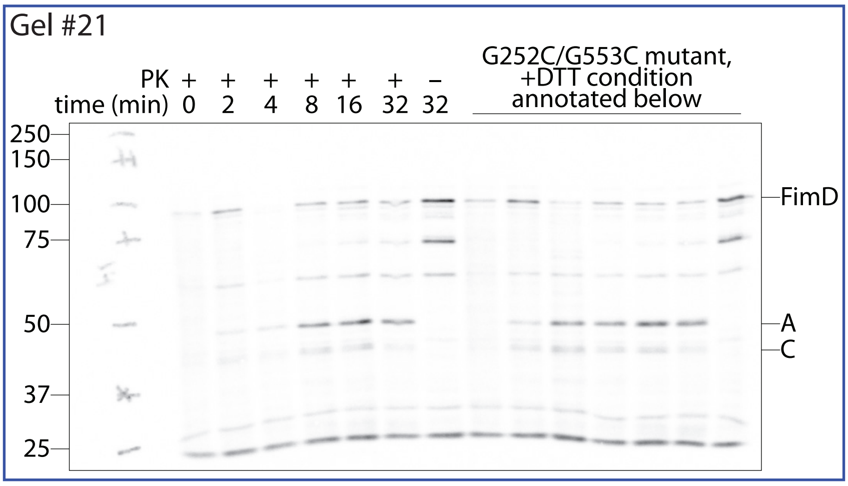

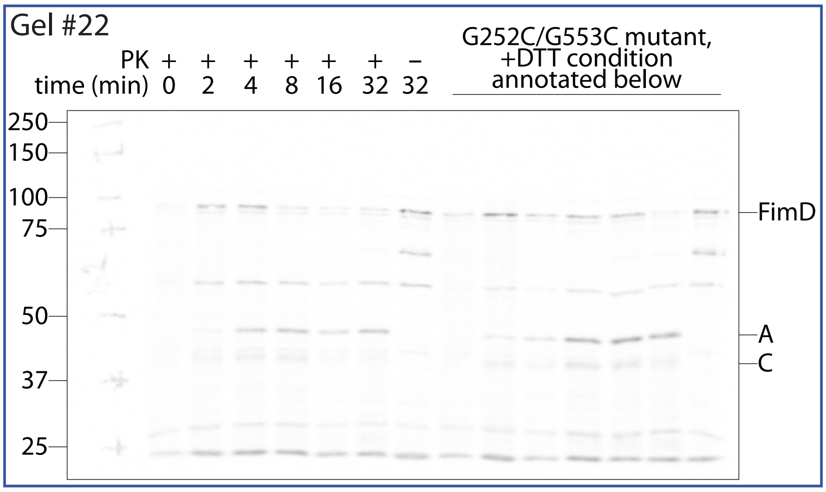

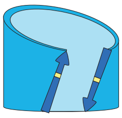

G252C/G553C with 5 mM DTT
3/3 biological replicates

Gel #20 - (raw image file for Fig. 2e, G252C/G553C non-reducing)

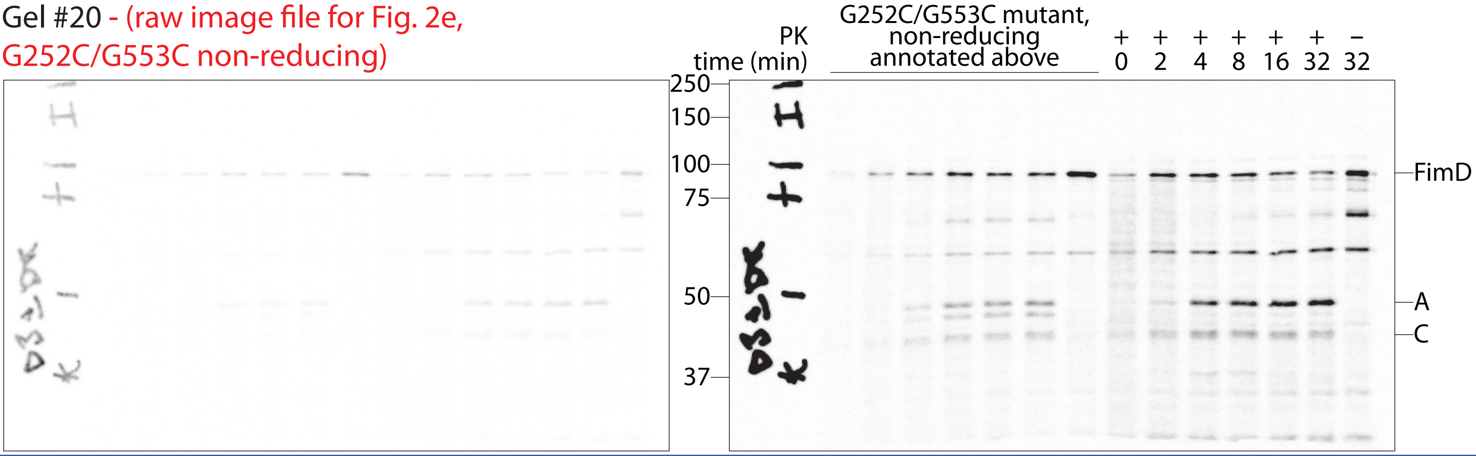

Gel #21

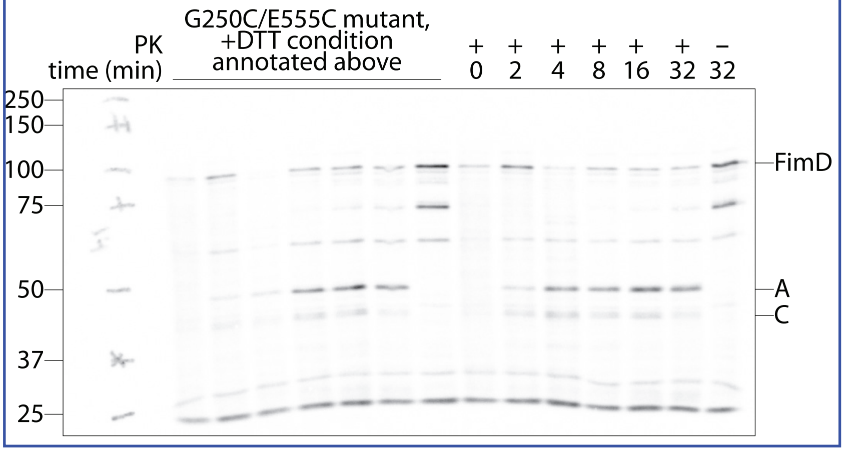

Gel #22

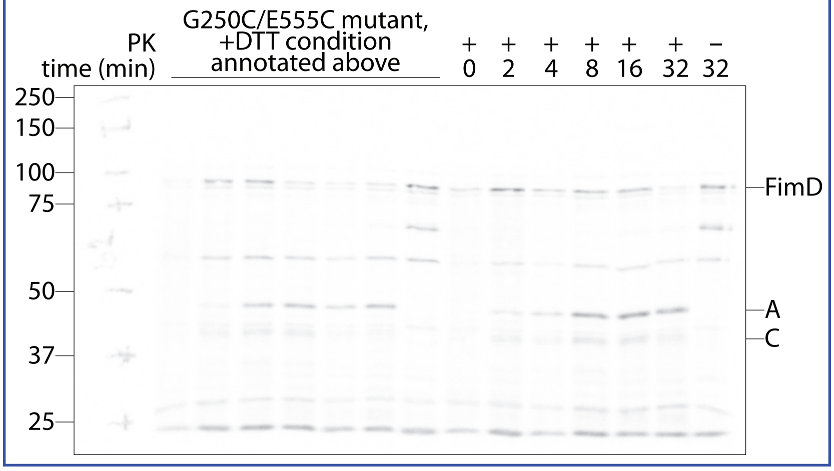

Single timepoints (+PK samples and -PK samples)
1/3 biological replicates (total: 1/3 biological replicates)

Gel #23 - (raw image file for Fig. S2C)

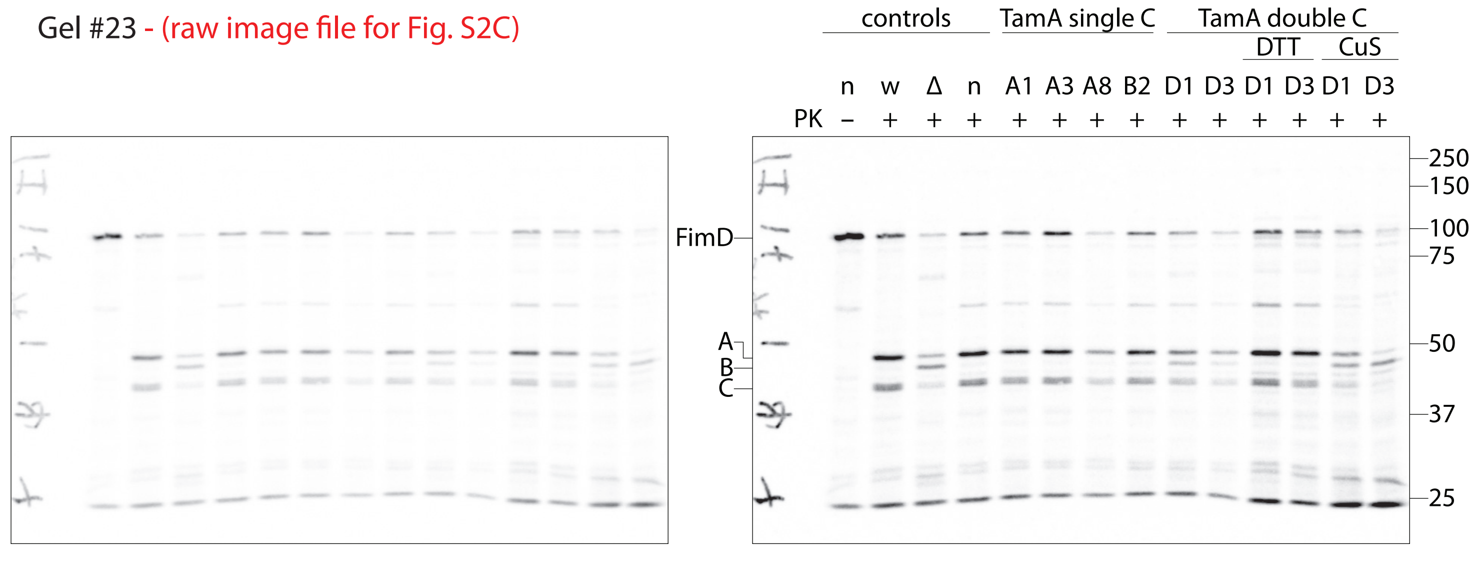

Gel #24 - (raw image file for Fig. S2C)

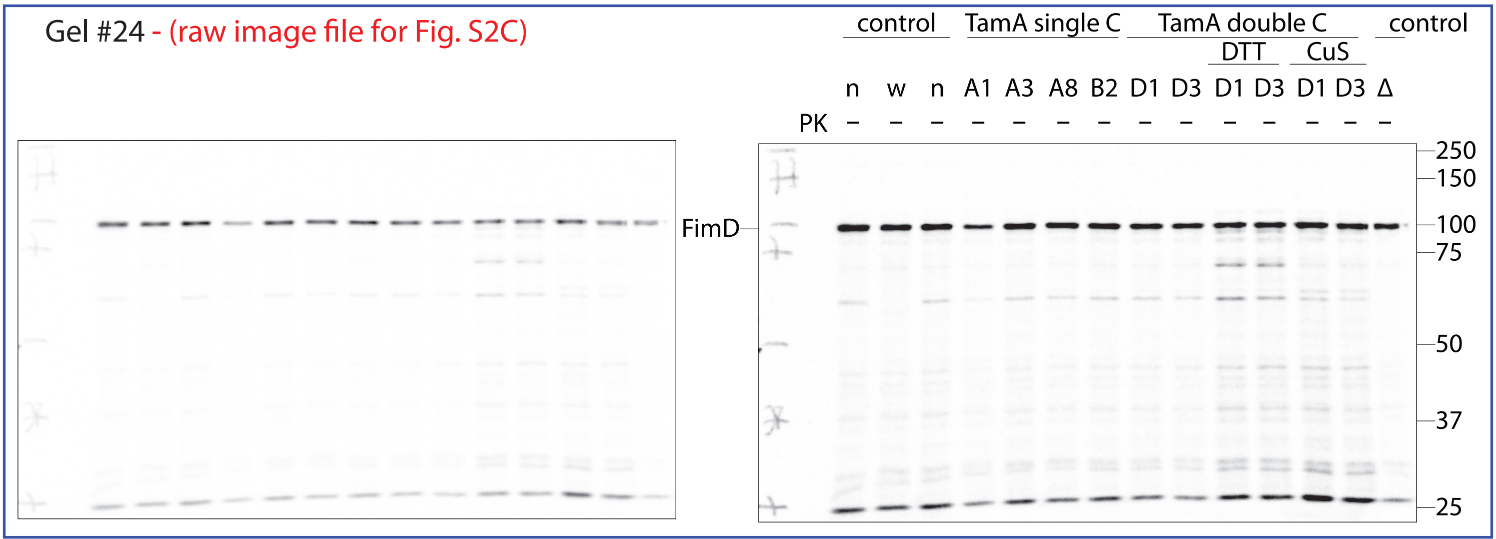

All strains contain pKS02 (fimD expression vector)

w = wildtype with pACYCDuet-1 (base expression vector)
Δ = Δ*tamA* with pACYCDuet-1 (base expression vector)
n = Δ*tamA* with pCJS69 (*tamA* expression vector)
A1 = Δ*tamA* with pCJS69 (*tamA*-G250C expression vector)
A3 = Δ*tamA* with pCJS69 (*tamA*-G252C expression vector)
A8 = Δ*tamA* with pCJS69 (*tamA*-G553C expression vector)
B2 = Δ*tamA* with pCJS69 (*tamA*-E555C expression vector)
D1 = Δ*tamA* with pCJS69 (*tamA*-G250C-E555C expression vector)
D3 = Δ*tamA* with pCJS69 (*tamA*-G252C-G553C expression vector)

Unless otherwise indicated (by "DTT" or "CuS"), samples were subjected to normal pulse chase
DTT = samples were supplemented with 5 mM DTT
CuS = samples were supplemented with 100 µM $CuSO_4$

Single timepoints (+PK samples and -PK samples)
1/3 biological replicates (total: 2/3 biological replicates)

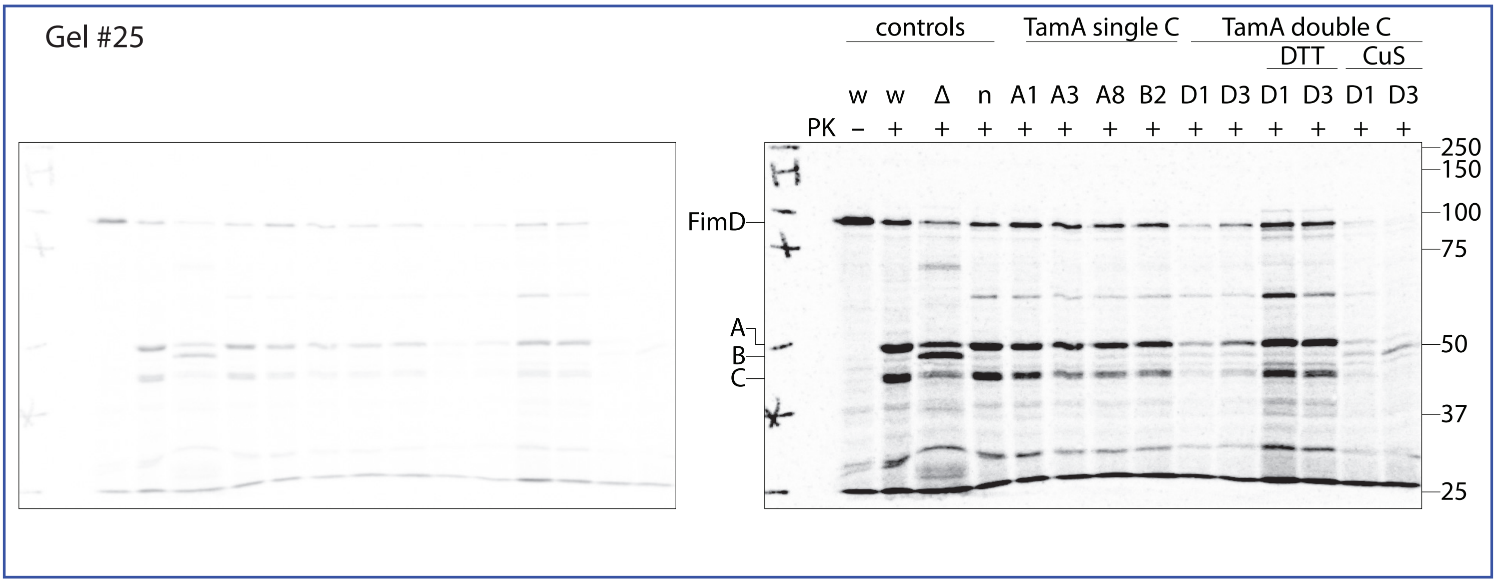

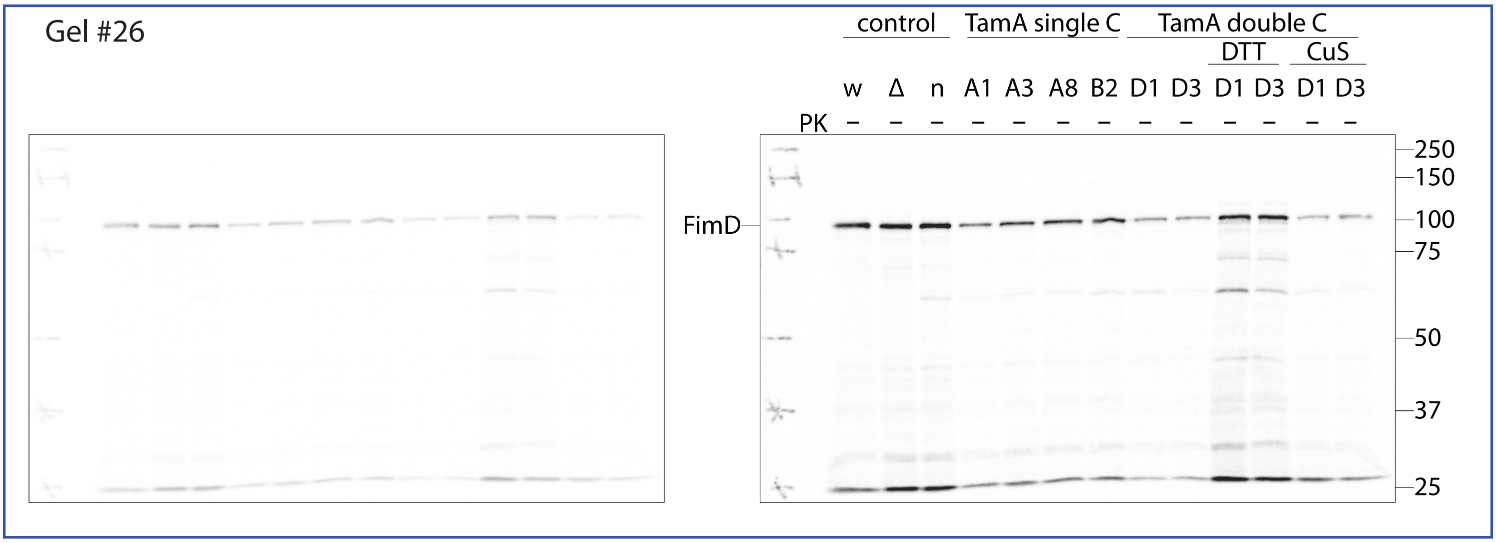

All strains contain pKS02 (fimD expression vector)

w = wildtype with pACYCDuet-1 (base expression vector)
Δ = Δ*tamA* with pACYCDuet-1 (base expression vector)
n = Δ*tamA* with pCJS69 (*tamA* expression vector)
A1 = Δ*tamA* with pCJS69 (*tamA*-G250C expression vector)
A3 = Δ*tamA* with pCJS69 (*tamA*-G252C expression vector)
A8 = Δ*tamA* with pCJS69 (*tamA*-G553C expression vector)
B2 = Δ*tamA* with pCJS69 (*tamA*-E555C expression vector)
D1 = Δ*tamA* with pCJS69 (*tamA*-G250C-E555C expression vector)
D3 = Δ*tamA* with pCJS69 (*tamA*-G252C-G553C expression vector)

Unless otherwise indicated (by "DTT" or "CuS"), samples were subjected to normal pulse chase
DTT = samples were supplemented with 5 mM DTT
CuS = samples were supplemented with 100 µM $CuSO_4$

Single timepoints (+PK samples and -PK samples)
1/3 biological replicates (total: 3/3 biological replicates)

Gel #27

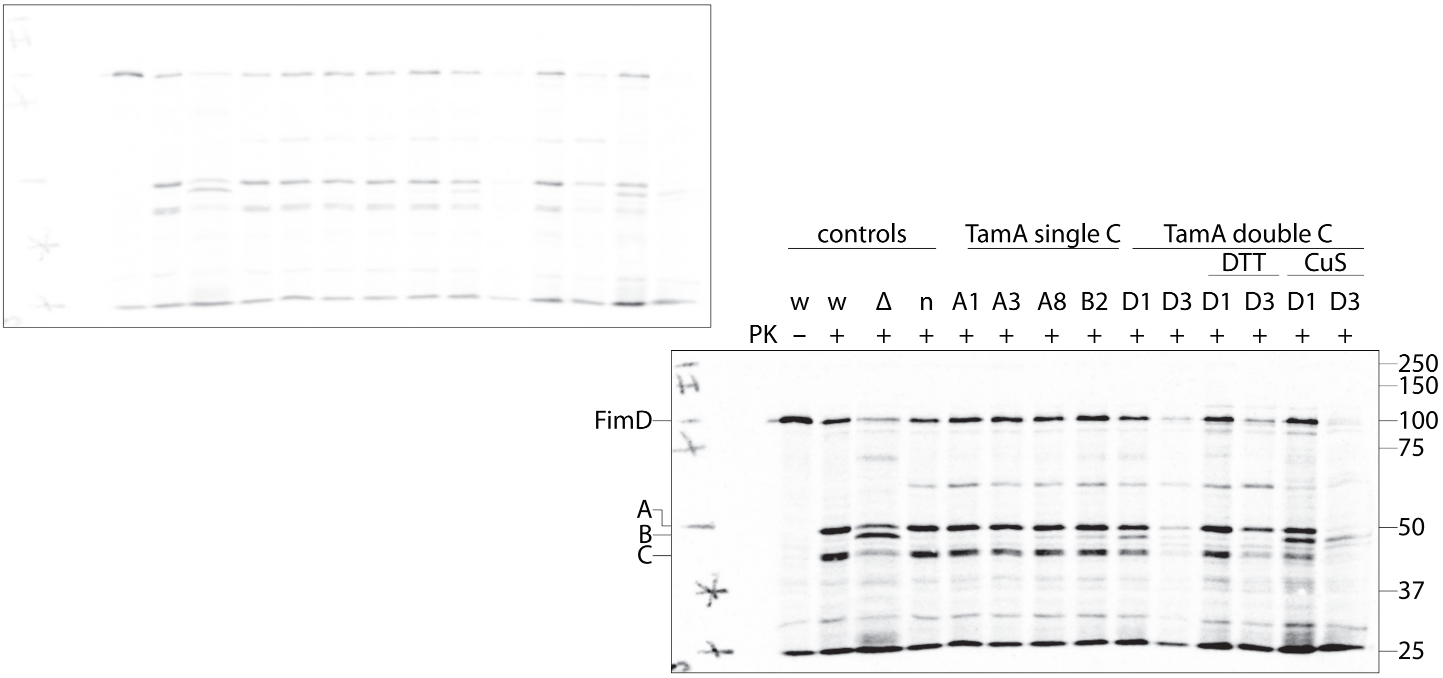

Gel #28

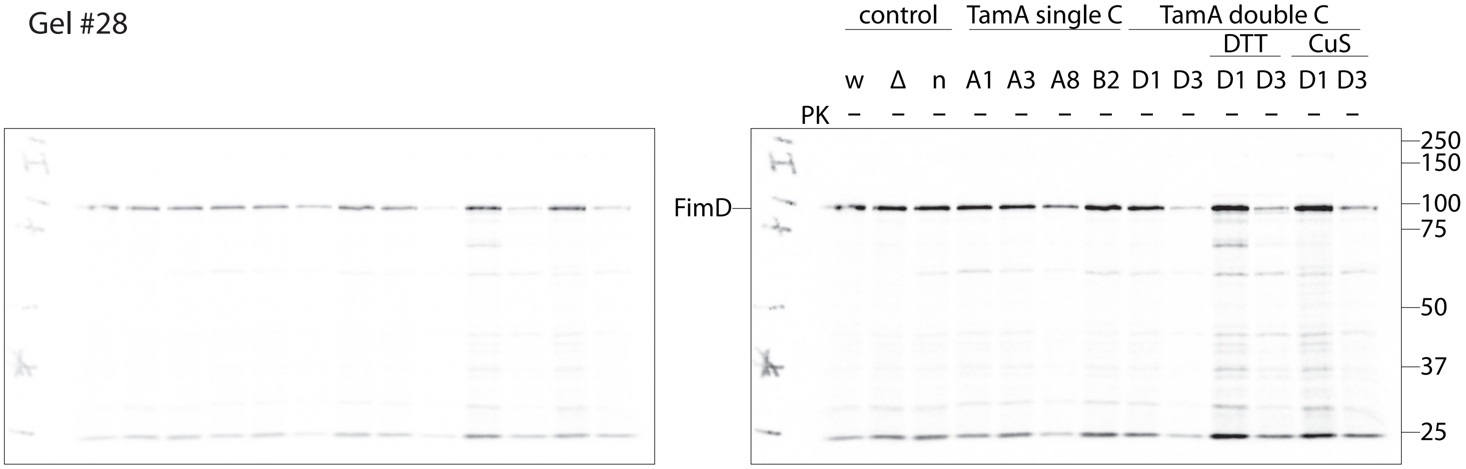

All strains contain pKS02 (fimD expression vector)

w = wildtype with pACYCDuet-1 (base expression vector)
Δ = Δ*tamA* with pACYCDuet-1 (base expression vector)
n = Δ*tamA* with pCJS69 (*tamA* expression vector)
A1 = Δ*tamA* with pCJS69 (*tamA*-G250C expression vector)
A3 = Δ*tamA* with pCJS69 (*tamA*-G252C expression vector)
A8 = Δ*tamA* with pCJS69 (*tamA*-G553C expression vector)
B2 = Δ*tamA* with pCJS69 (*tamA*-E555C expression vector)
D1 = Δ*tamA* with pCJS69 (*tamA*-G250C-E555C expression vector)
D3 = Δ*tamA* with pCJS69 (*tamA*-G252C-G553C expression vector)

Unless otherwise indicated (by "DTT" or "CuS"), samples were subjected to normal pulse chase
DTT = samples were supplemented with 5 mM DTT
CuS = samples were supplemented with 100 μM $CuSO_4$
