# Supplementary material for: A noncanonical chaperone interacts with drug efflux pumps during their assembly into bacterial outer membranes
Source: PLoS Biol. 2022 Jan 21;20(1):e3001523. doi: 10.1371/journal.pbio.3001523 (PMC8809574; doi:10.1371/journal.pbio.3001523)

TamA oxidised v reduced: Fig 2B, 2C, S1A Fig (2/2 immunoblots)

Immunoblot #1 - (raw image file for Fig. 2B, 2C, S1A Fig panel "1" and S1A Fig panel "2")

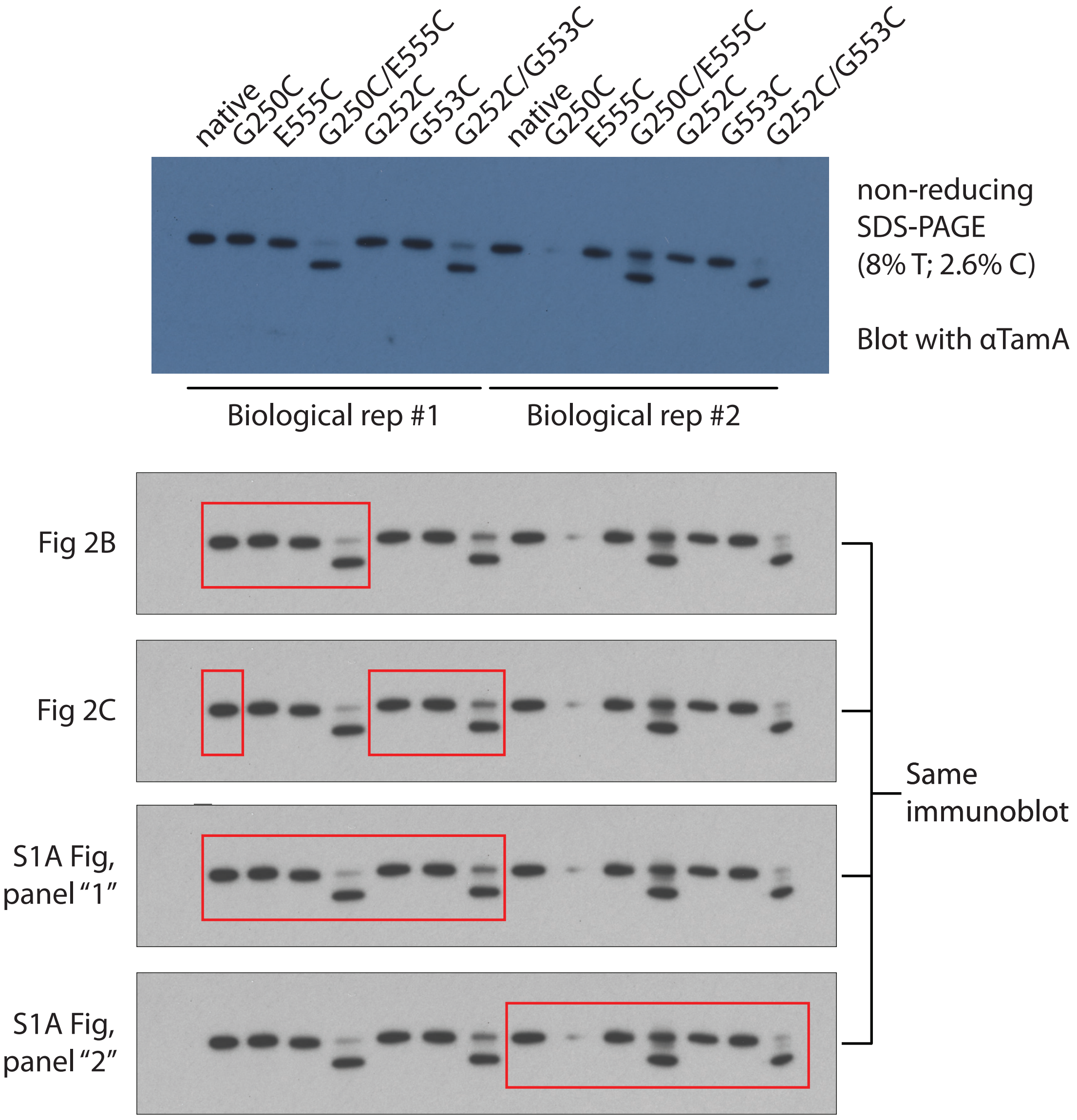

Immunoblot #2 - (raw image file for S1A Fig, panel "3")

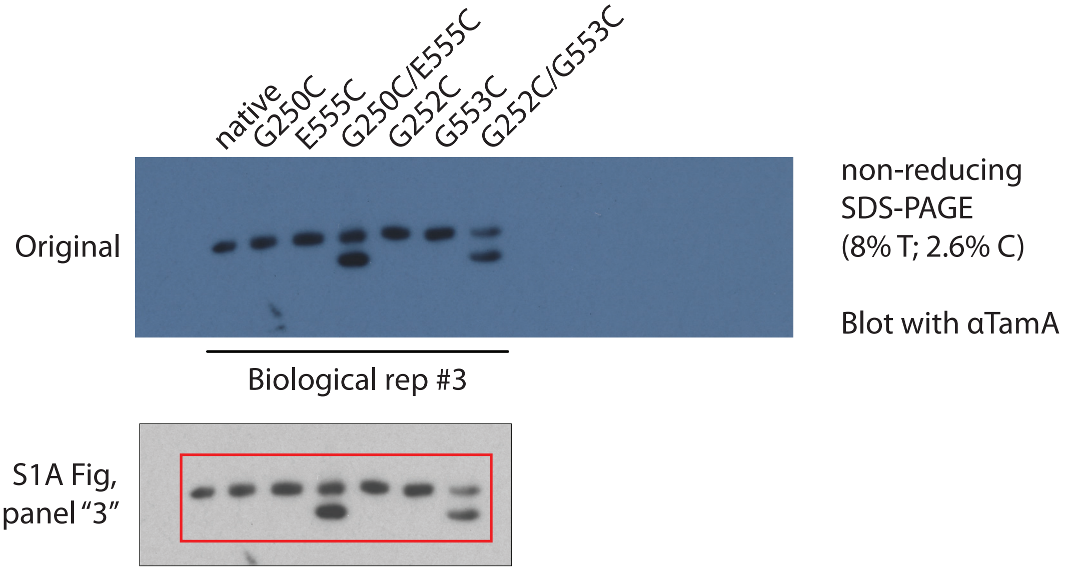

TamA-TolC crosslinks Fig 3C, 3D (3/18 immunoblots - total of 3/18 immunoblots)

Immunoblots #3-5 - (biological replicate 1 of Fig 3C, not included in figure itself)

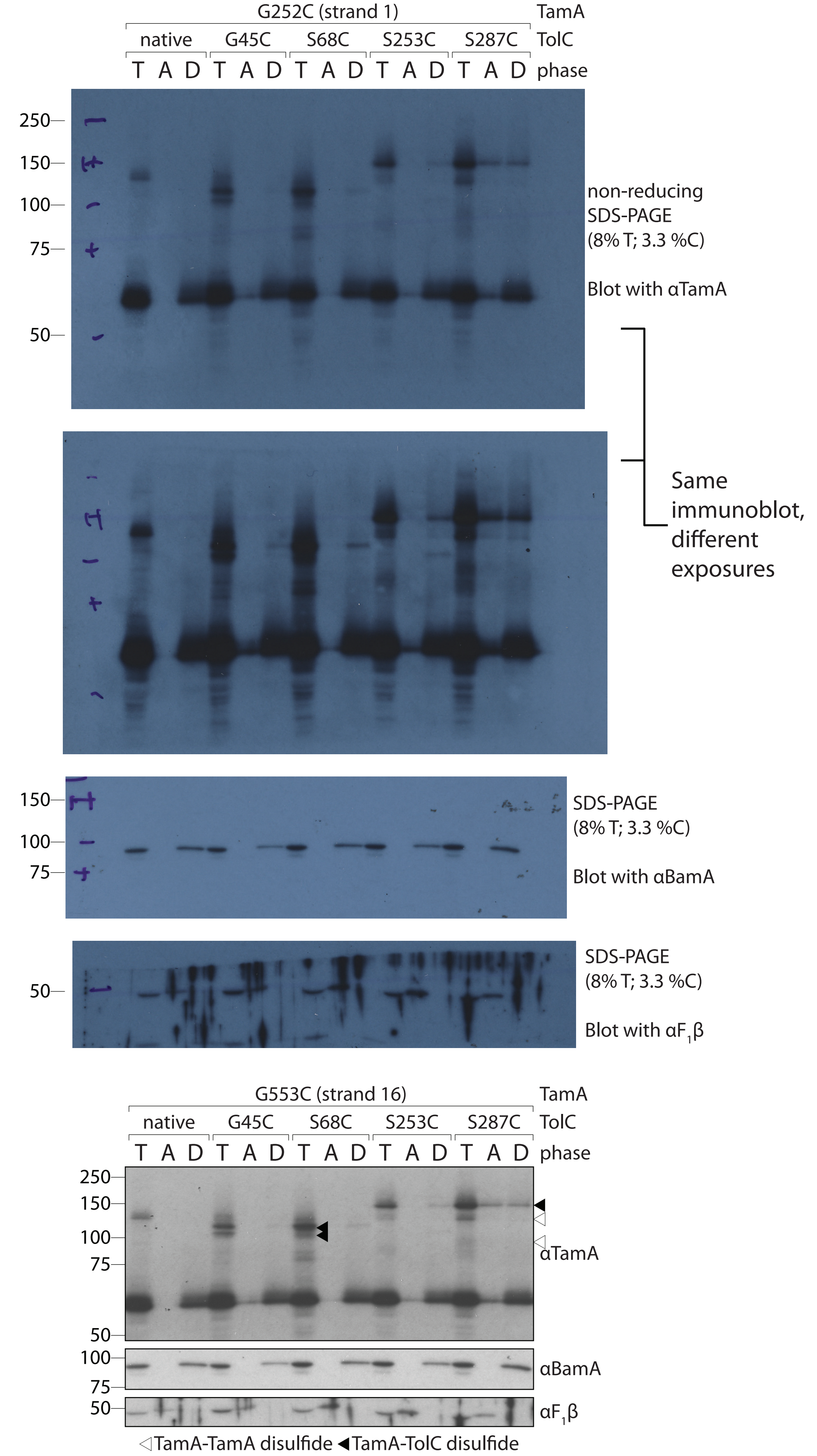

TamA-TolC crosslinks Fig 3C, 3D (3/18 immunoblots - total of 6/18 immunoblots)

Immunoblots #6-8 - (biological replicate 2 of Fig 3C)

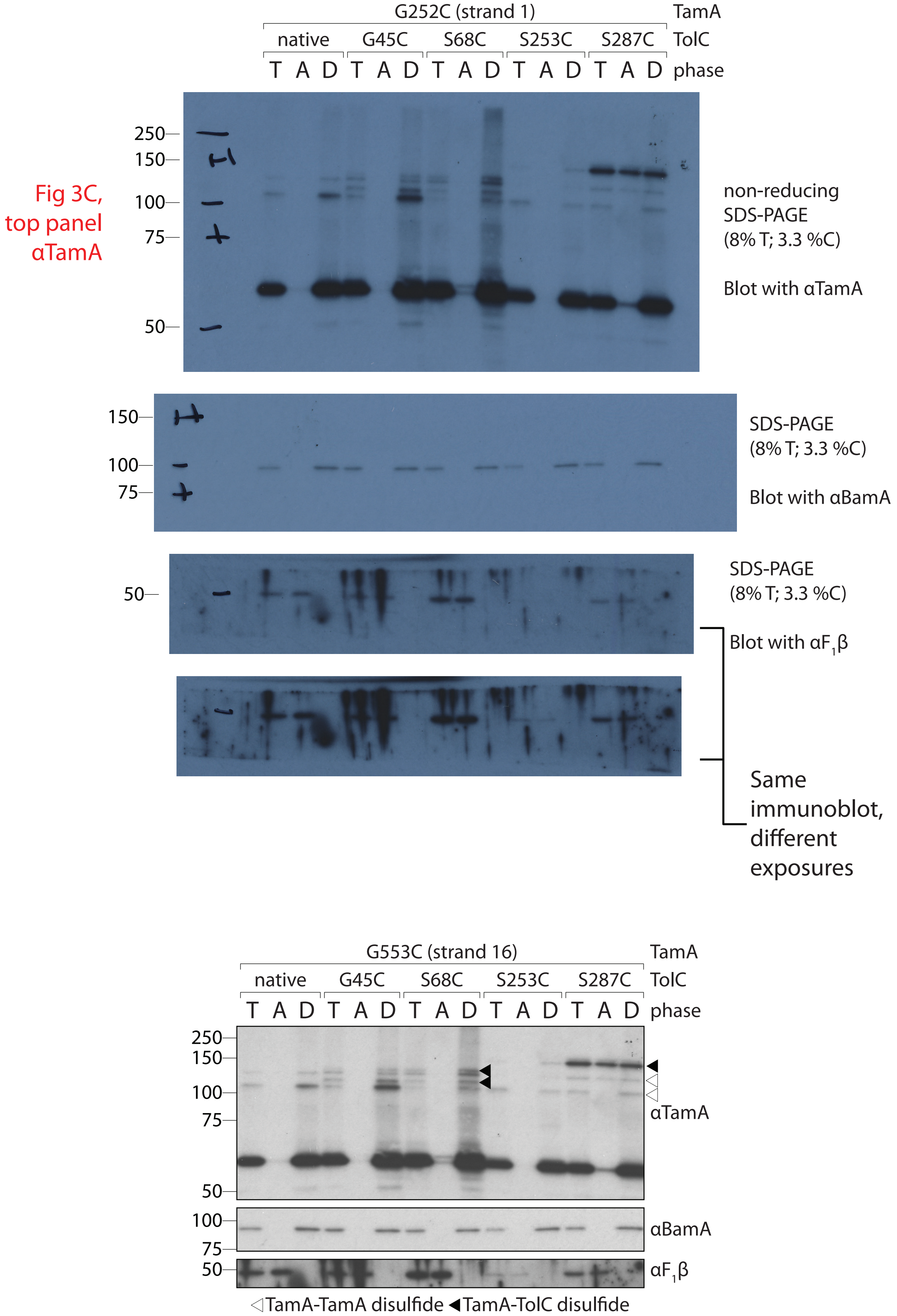

TamA-TolC crosslinks Fig 3C, 3D (3/18 immunoblots - total of 9/18 immunoblots)

Immunoblots #6-8 - (biological replicate 3 of Fig 3C)

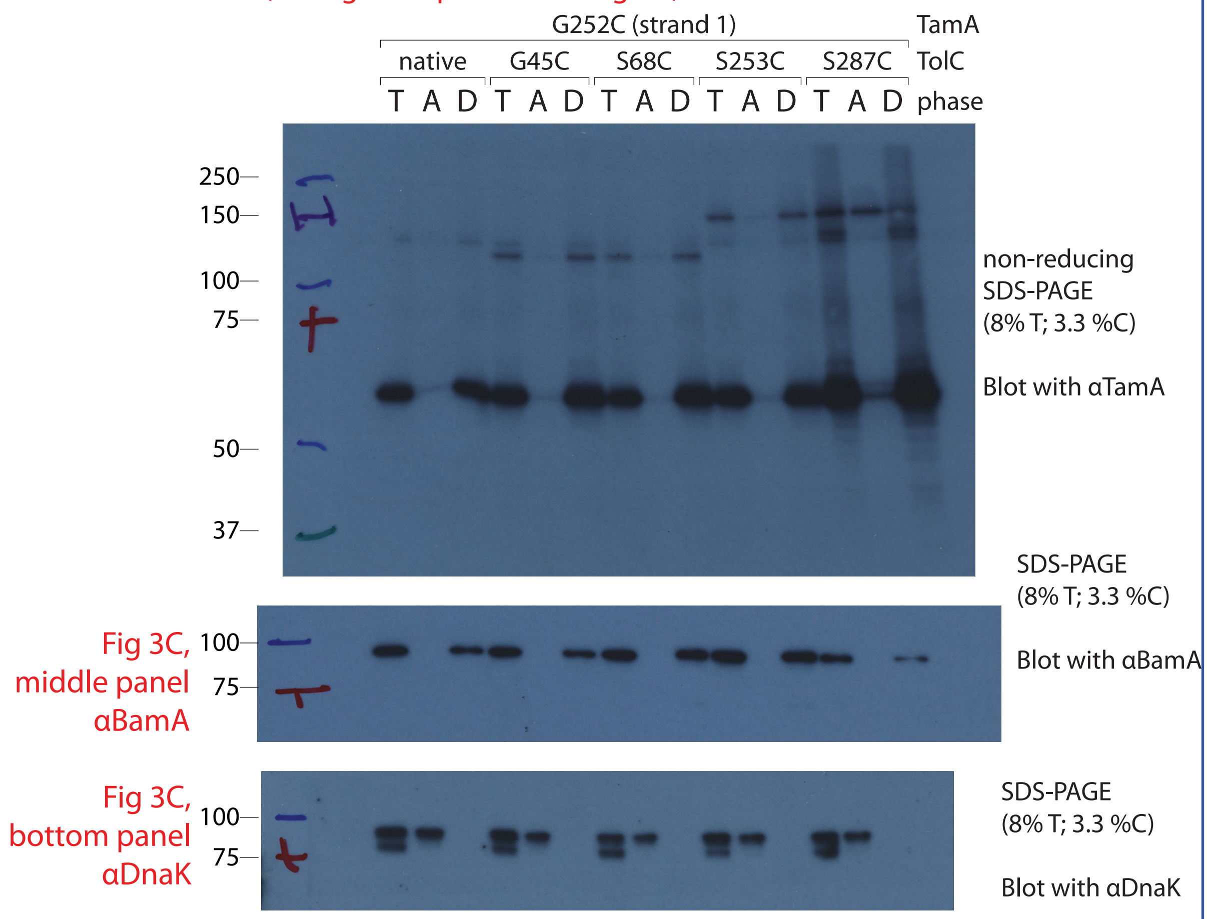

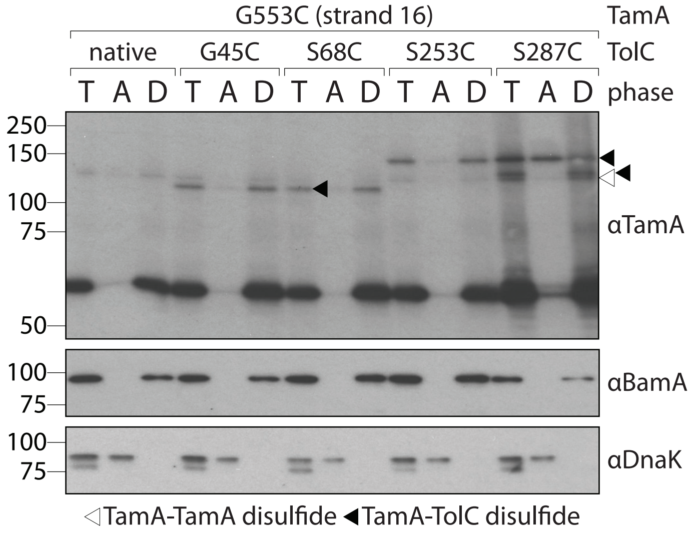

TamA-TolC crosslinks Fig 3C, 3D (3/18 immunoblots - total of 12/18 immunoblots)

Immunoblots #12-14 - (biological replicate 1 of Fig 3D, not included in figure itself)

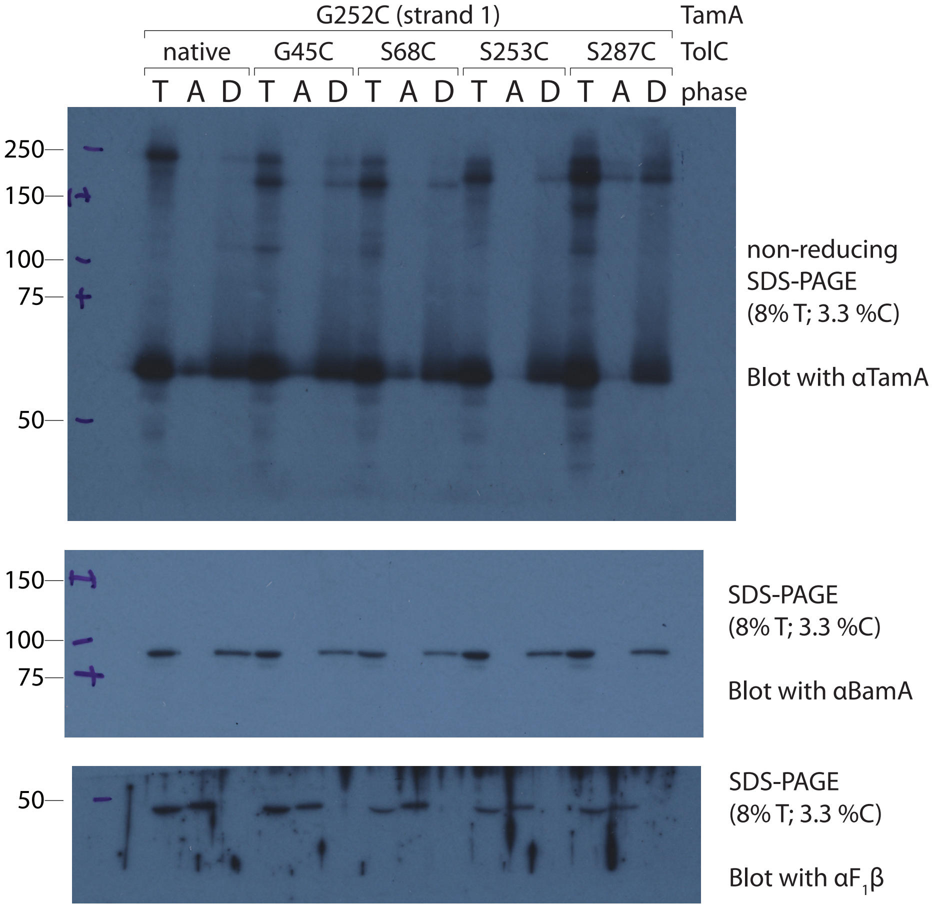

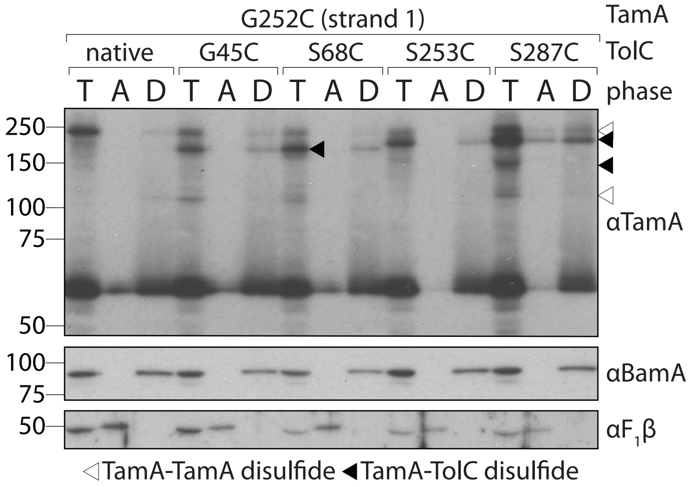

TamA-TolC crosslinks Fig 3C, 3D (3/18 immunoblots - total of 15/18 immunoblots)

Immunoblots #15-17 - (biological replicate 2 of Fig 3D)

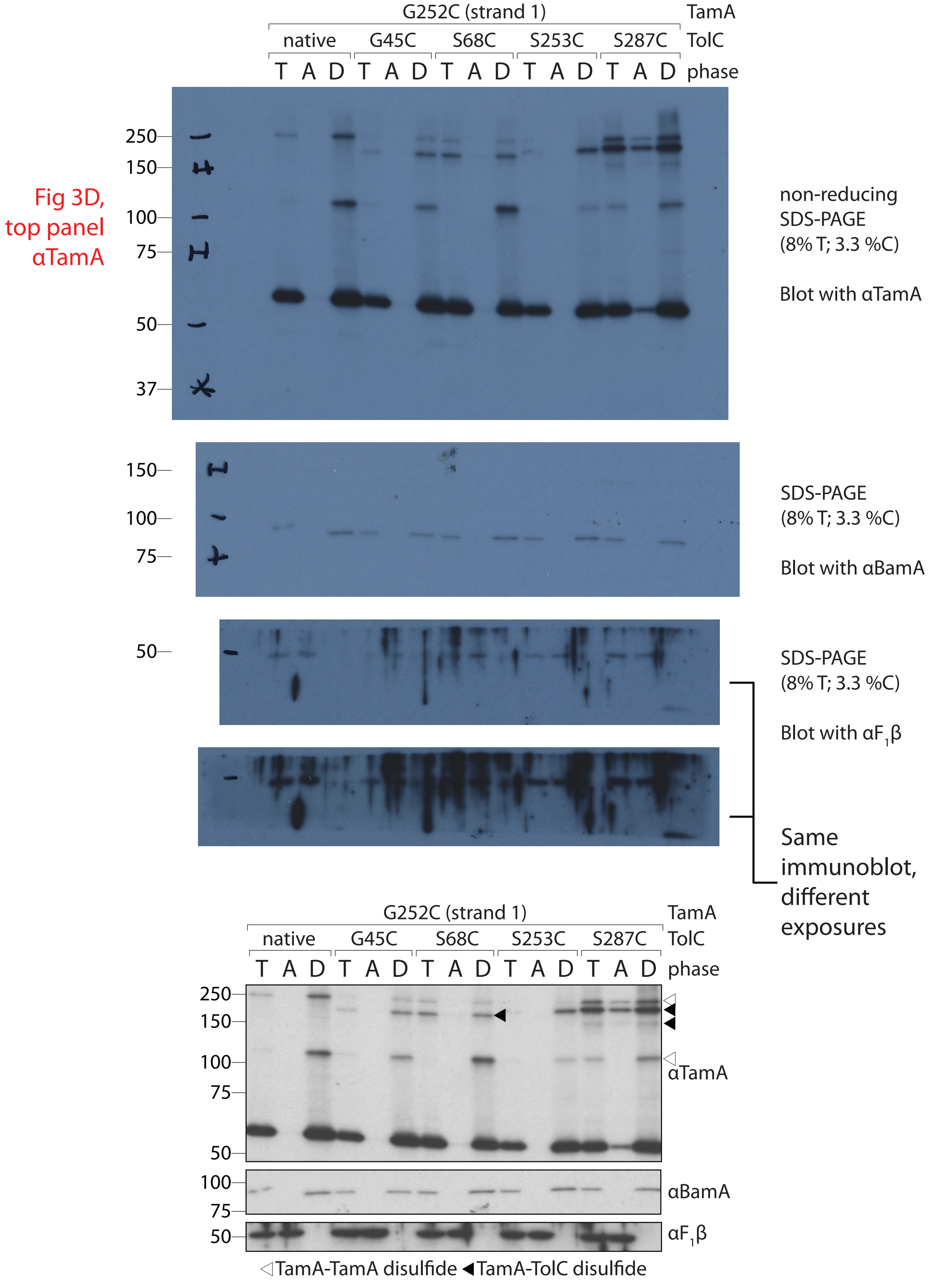

TamA-TolC crosslinks Fig 3C, 3D (3/18 immunoblots - total of 18/18 immunoblots)

Immunoblots #18-20 - (biological replicate 3 of Fig 3D)

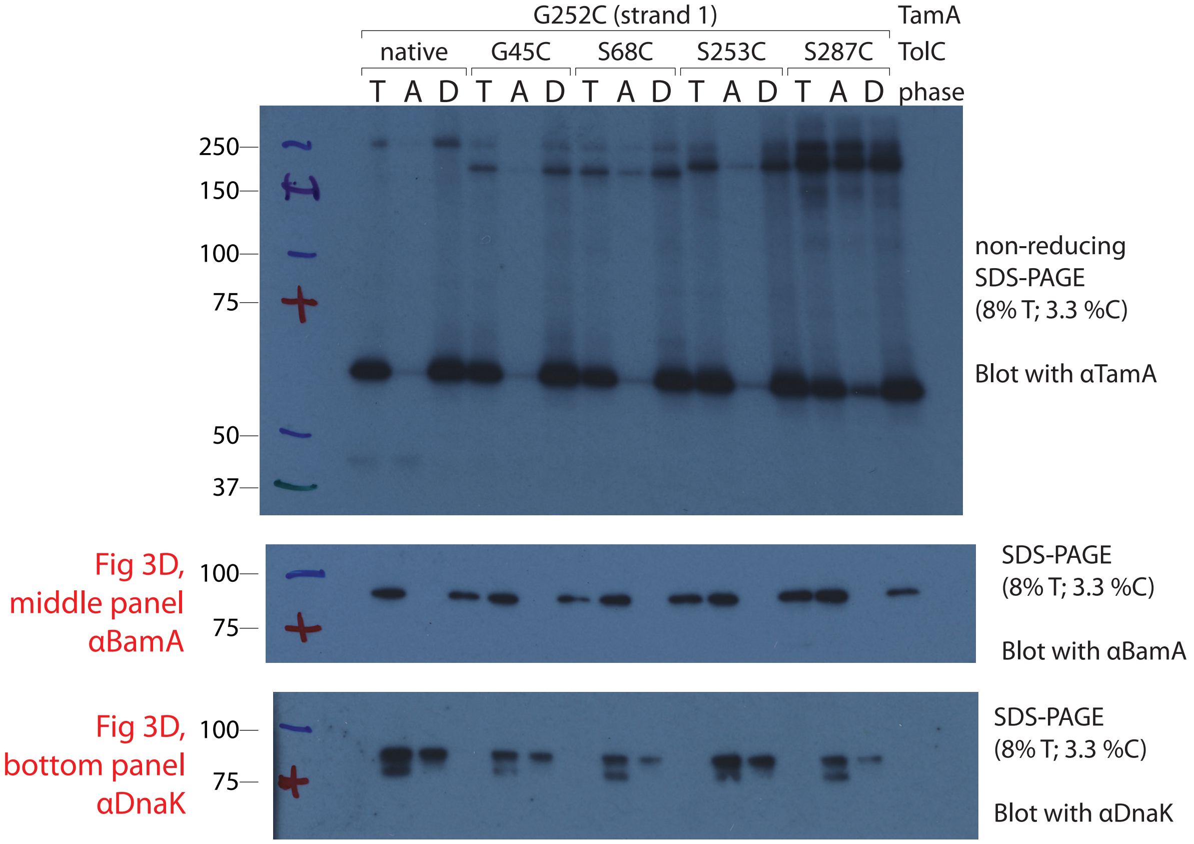

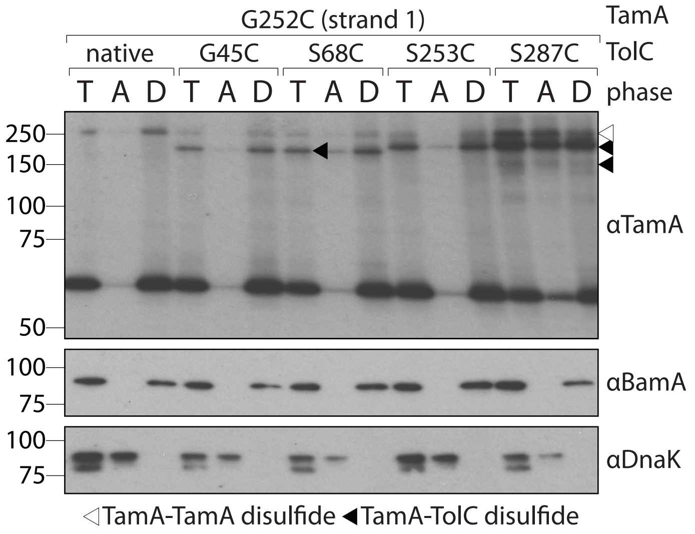

Immunoblot #21 - (S3 Fig; batch 1; αBamA; left panel)

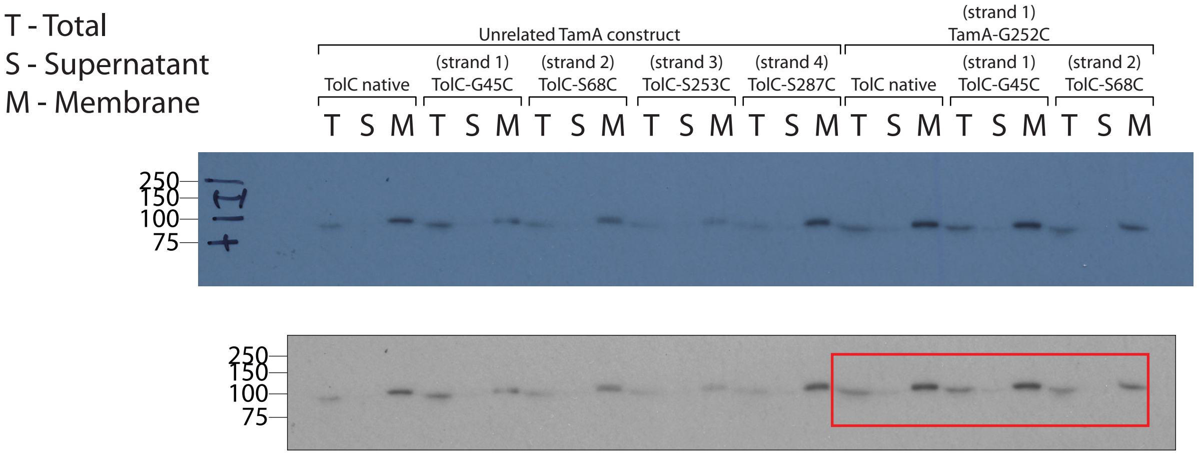

Immunoblot #22 - (S3 Fig; batch 1; αBamA; middle panel)

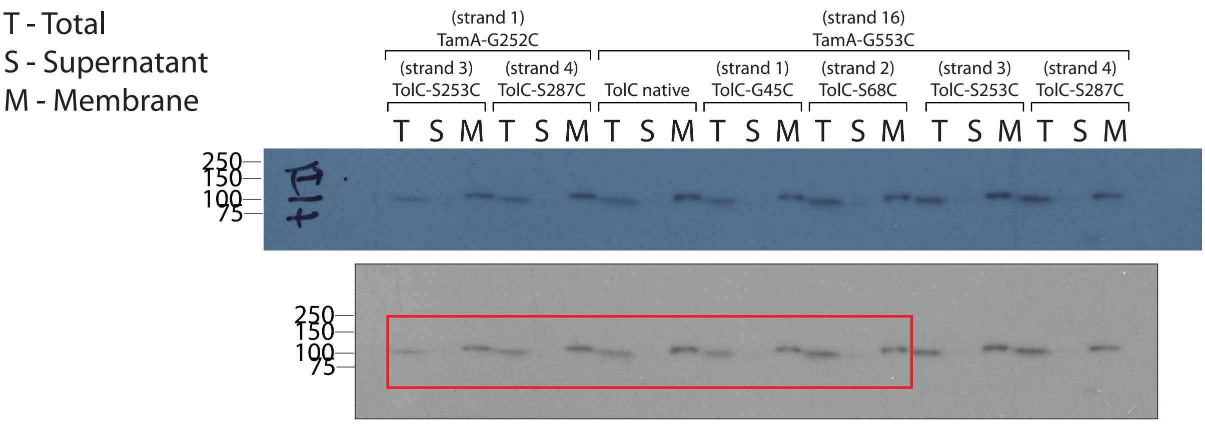

Immunoblot #23 - (S3 Fig; batches 1-3; αBamA; right panel)

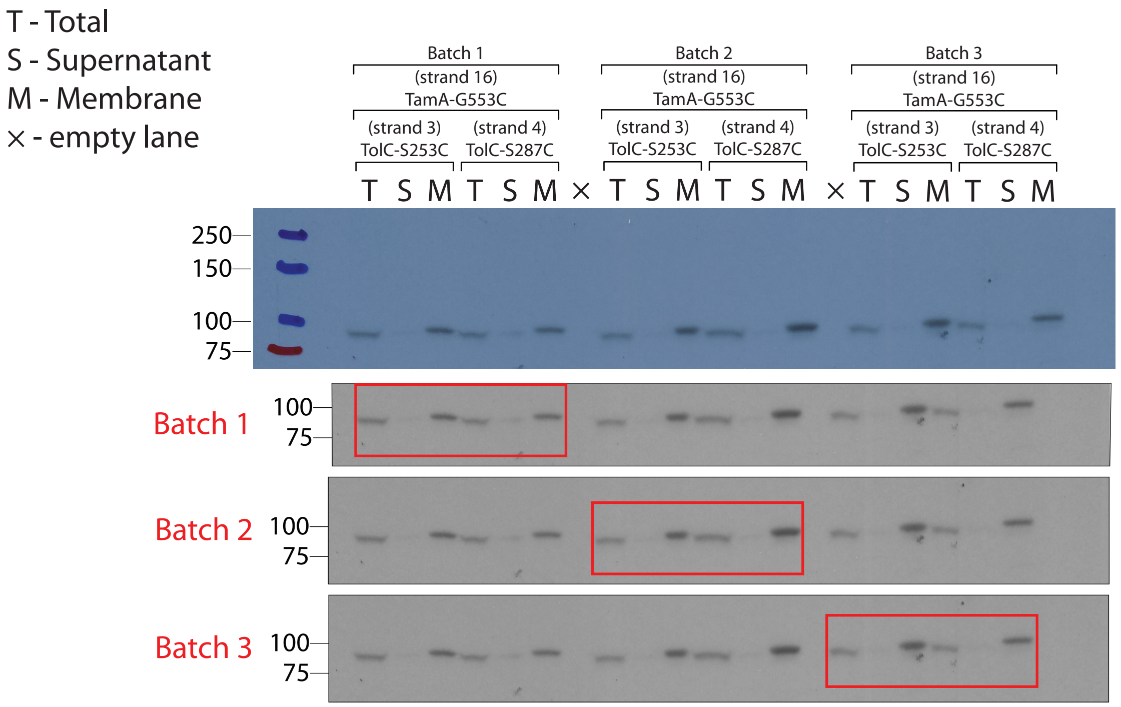

Immunoblot #24 - (S3 Fig; batch 2; αBamA; left panel)

T - Total
S - Supernatant
M - Membrane

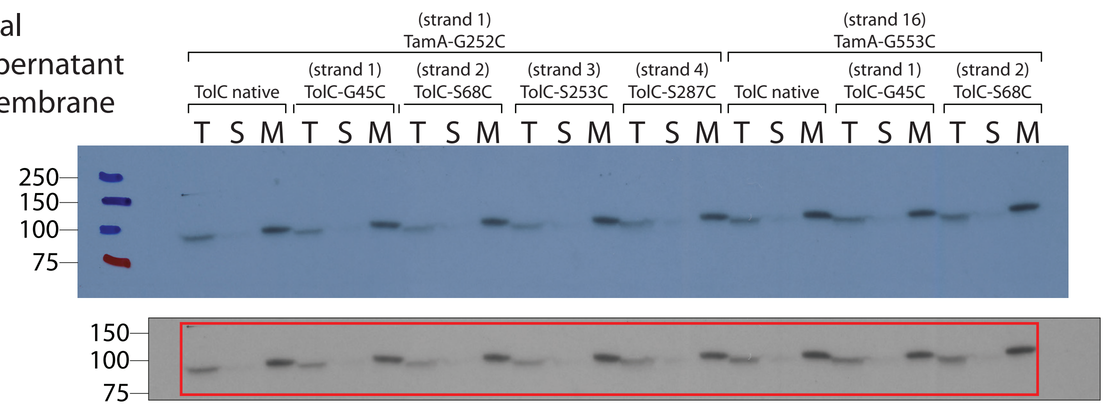

Immunoblot #25 - (S3 Fig; batch 3; αBamA; left panel)

T - Total
S - Supernatant
M - Membrane

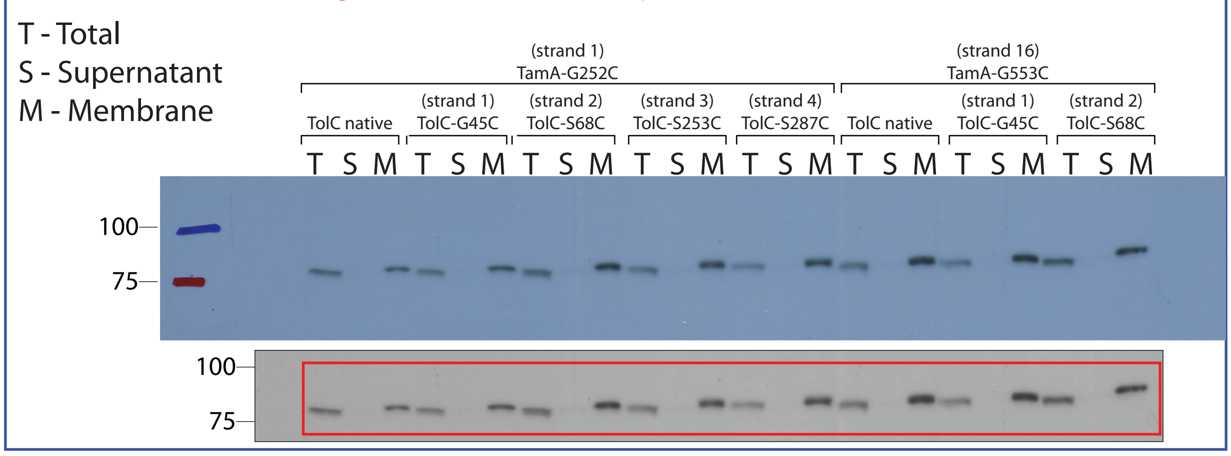

Immunoblot #26 - (S3 Fig; batch 1; αSurA; left panel)

T - Total
S - Supernatant
M - Membrane

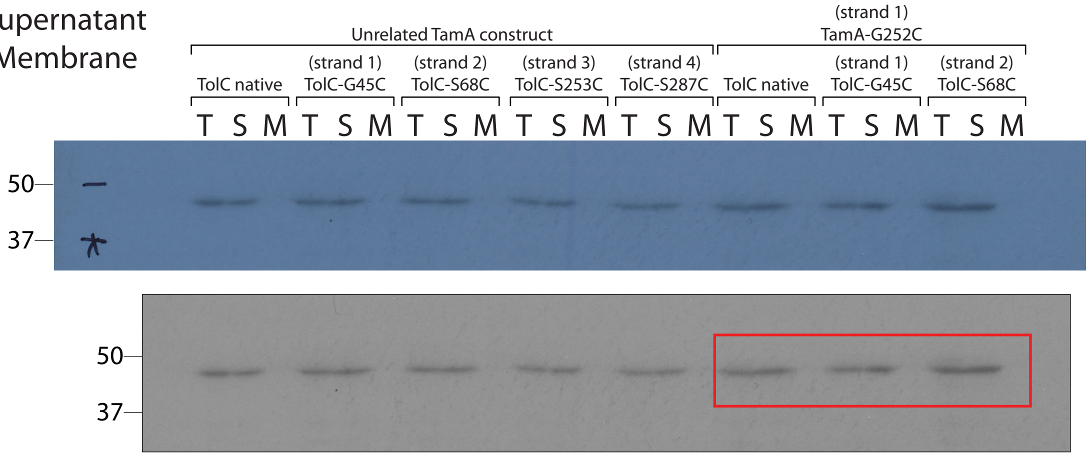

Immunoblot #27 - (S3 Fig; batch 1; αSurA; left panel)

T - Total
S - Supernatant
M - Membrane

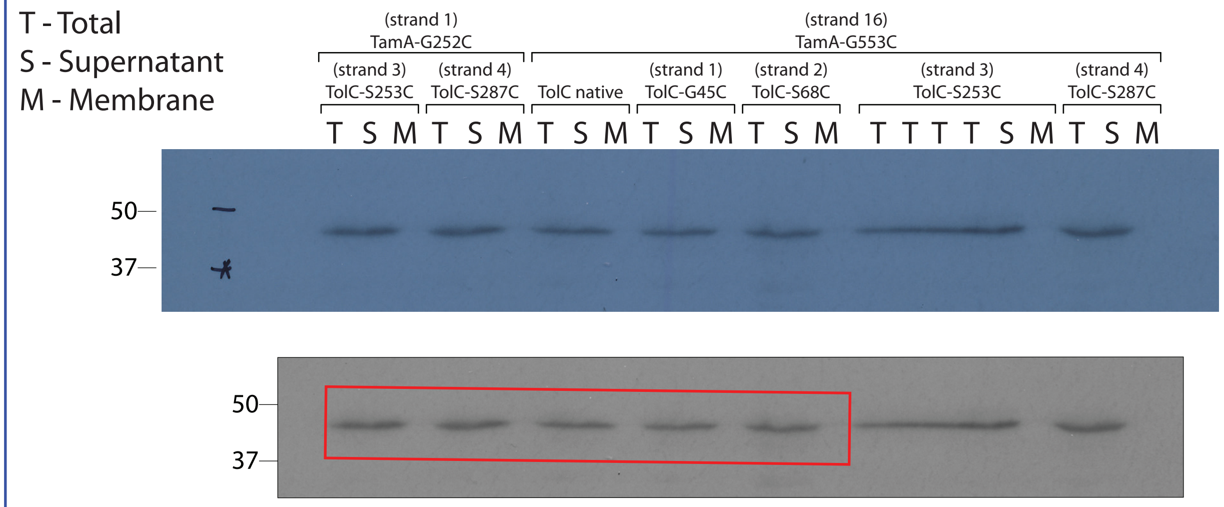

NB: Quadruplicate T - there were potential issues with the lanes/wells hence multiple loading

Immunoblot #28 - (S3 Fig; batches 1-3; αSurA; right panel)

T - Total
S - Supernatant
M - Membrane
× - empty lane

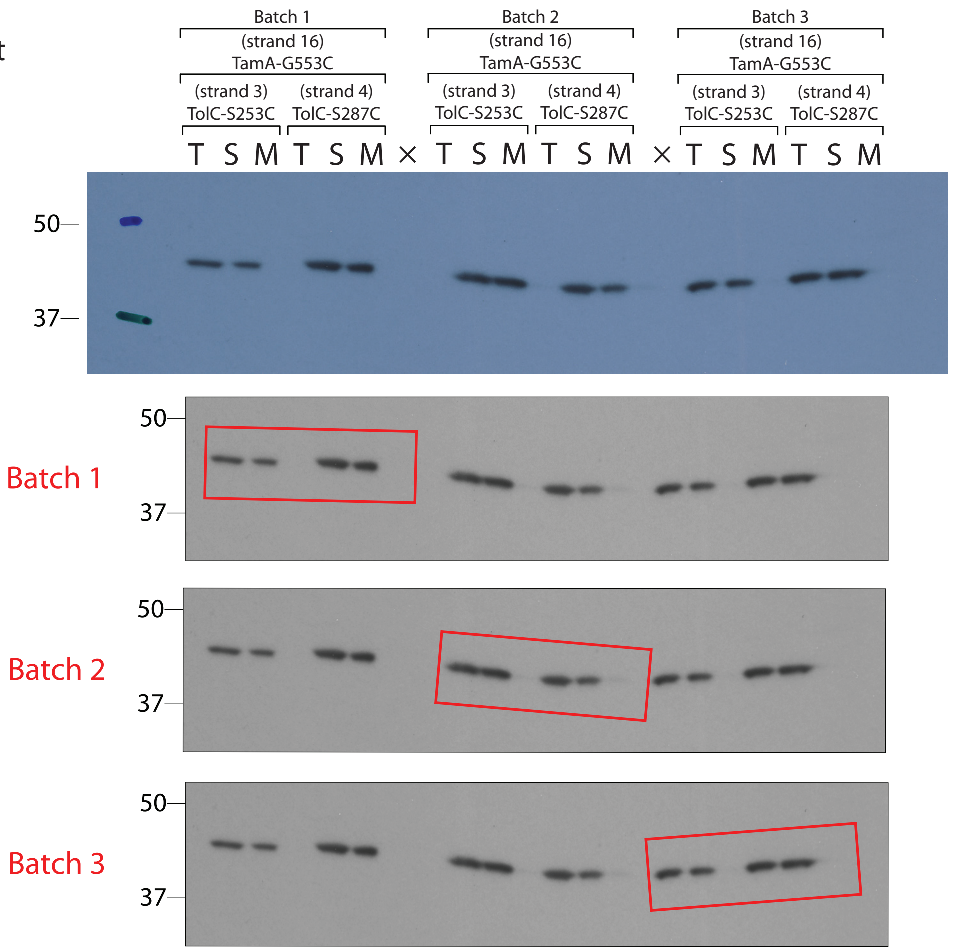

NB: Ran lanes close to the end of the gel where it started to bend

Immunoblot #29 - (S3 Fig; batch 2; αSurA; left panel)

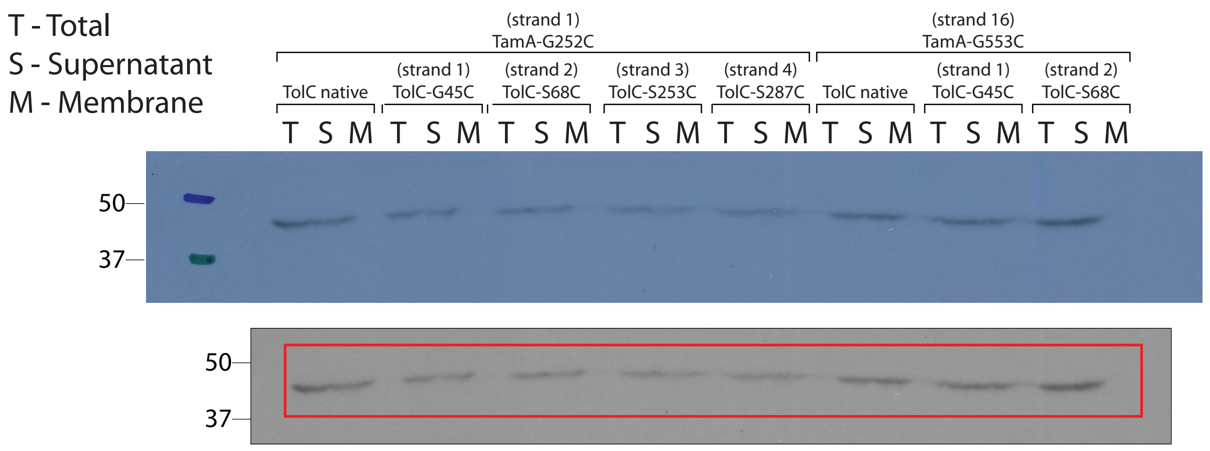

Immunoblot #30 - (S3 Fig; batch 3; αSurA; left panel)

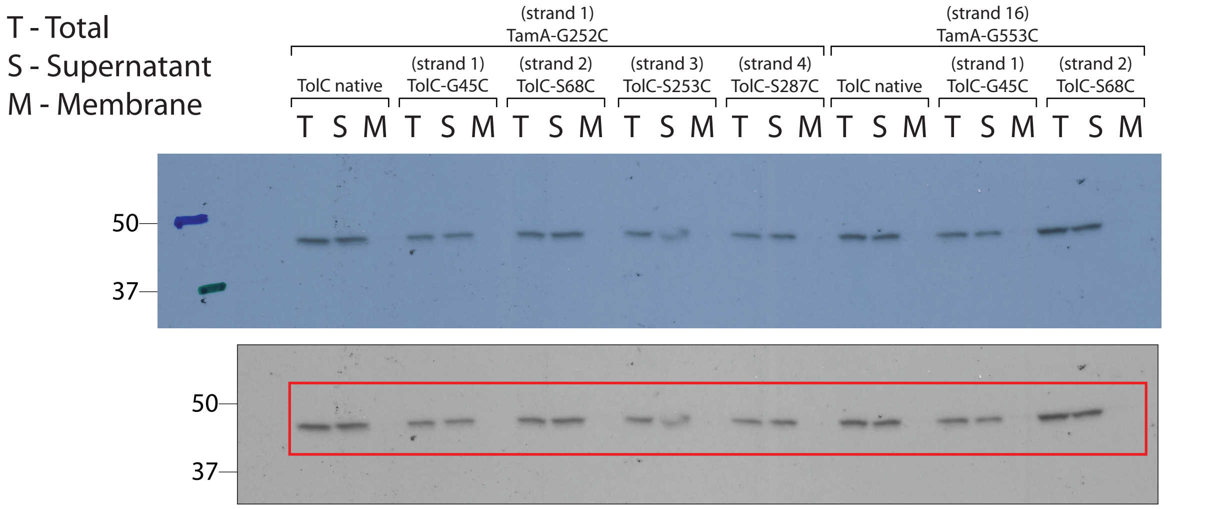

Immunoblot #31 - (S3 Fig; batch 1; αGroES; left and middle panels)

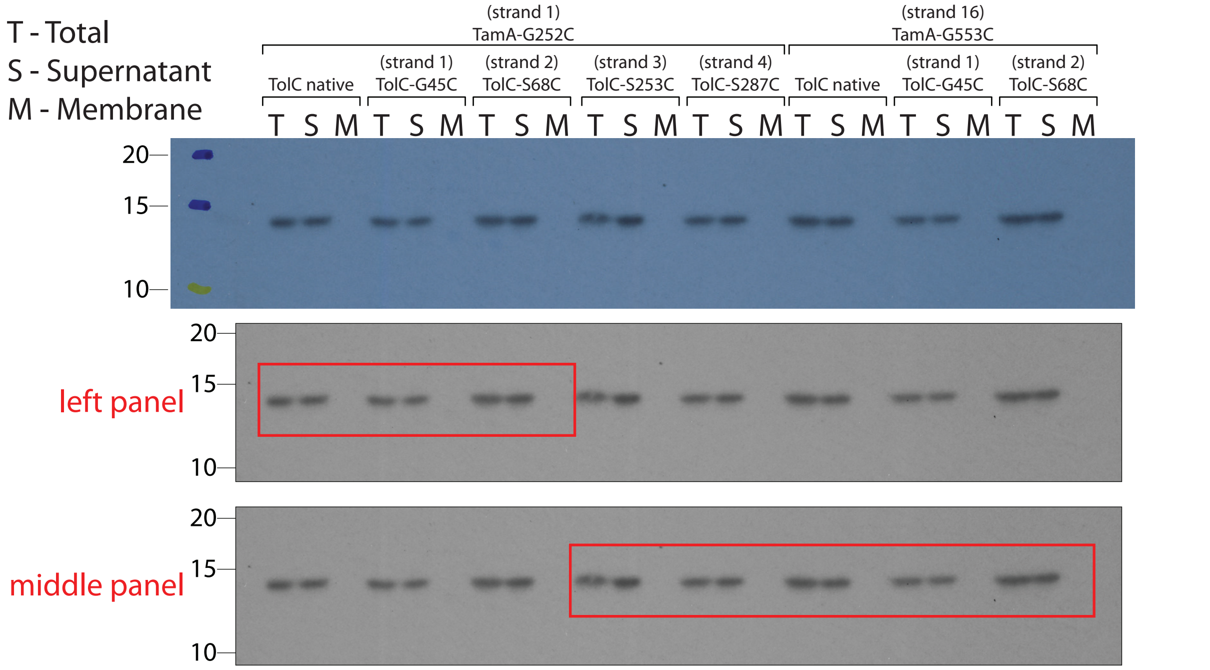

Immunoblot #32 - (S3 Fig; batch 1; αGroES; right panel)

T - Total
S - Supernatant
M - Membrane
× - empty lane

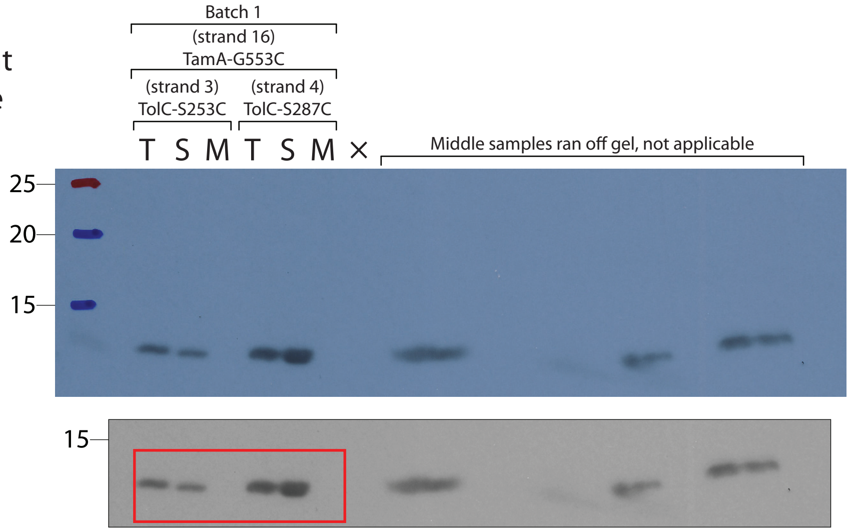

Immunoblot #33 - (S3 Fig; batch 2; αGroES; left panel)

T - Total
S - Supernatant
M - Membrane

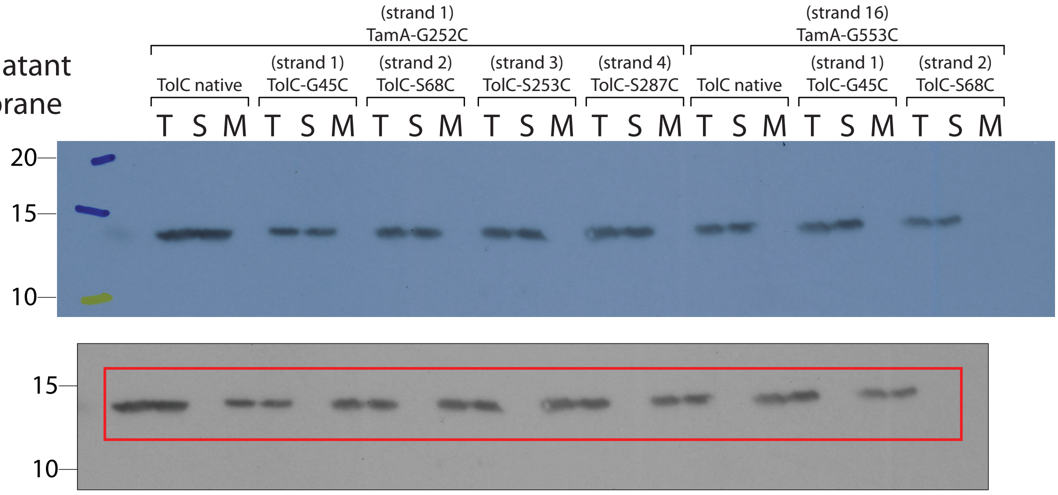

Immunoblot #34 - (S3 Fig; batch 3; αGroES; left panel)

T - Total
S - Supernatant
M - Membrane

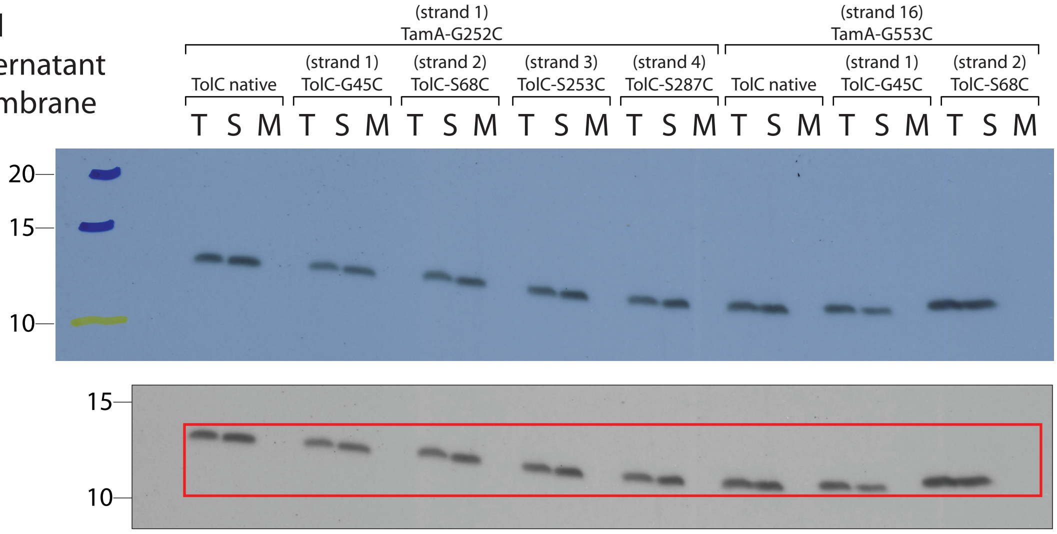

Immunoblot #35 - (S3 Fig; batches 2-3; αGroES; right panels)

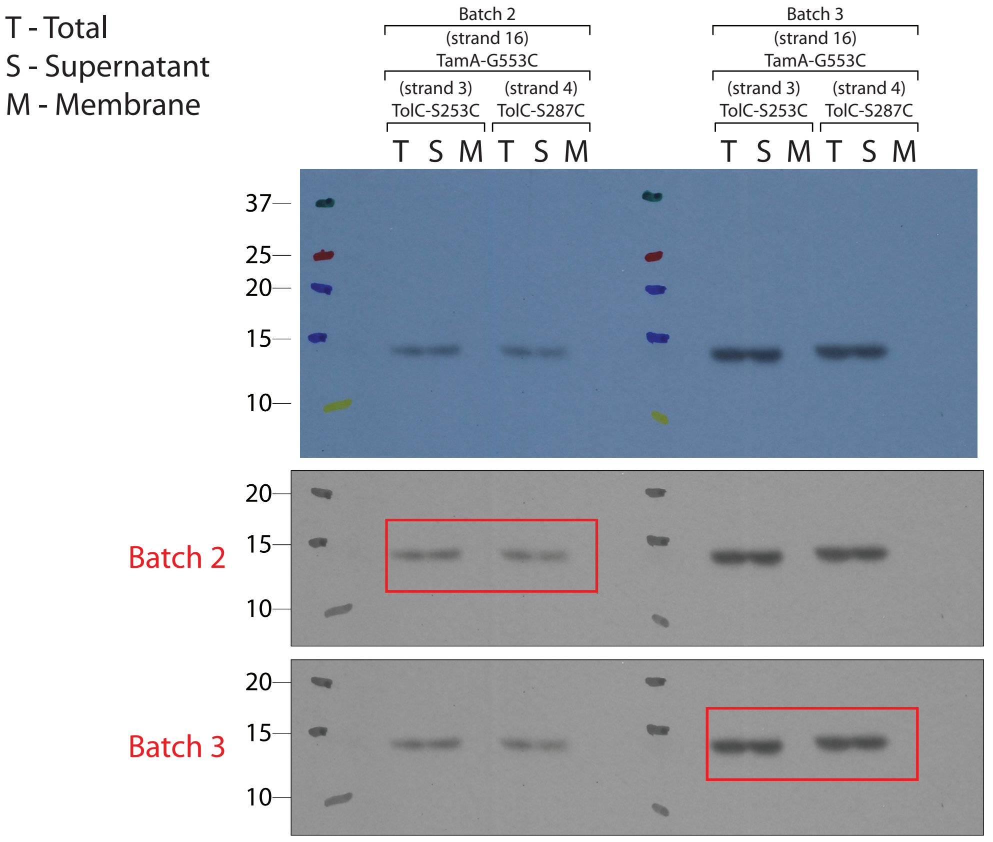

Immunoblot #36 - (S4 Fig; αTamA; left and right panels)

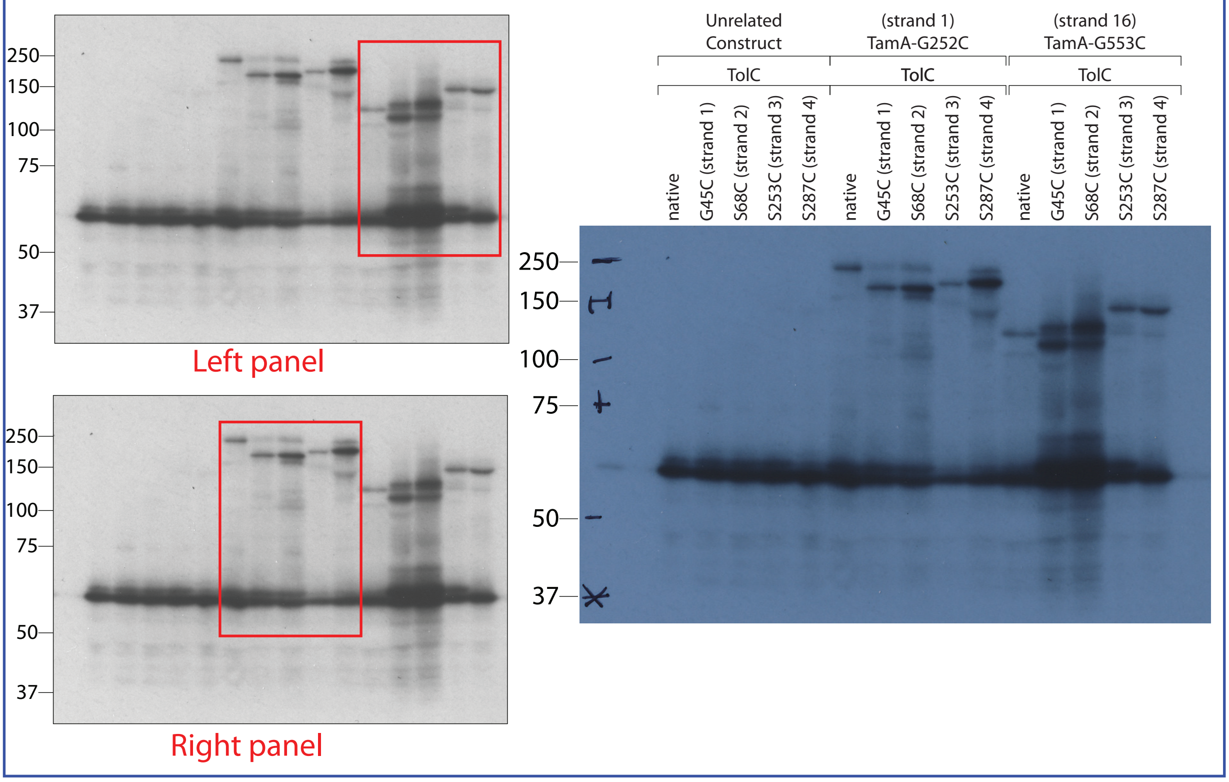

Immunoblot #37 - (S4 Fig; αTolC; left and right panels)

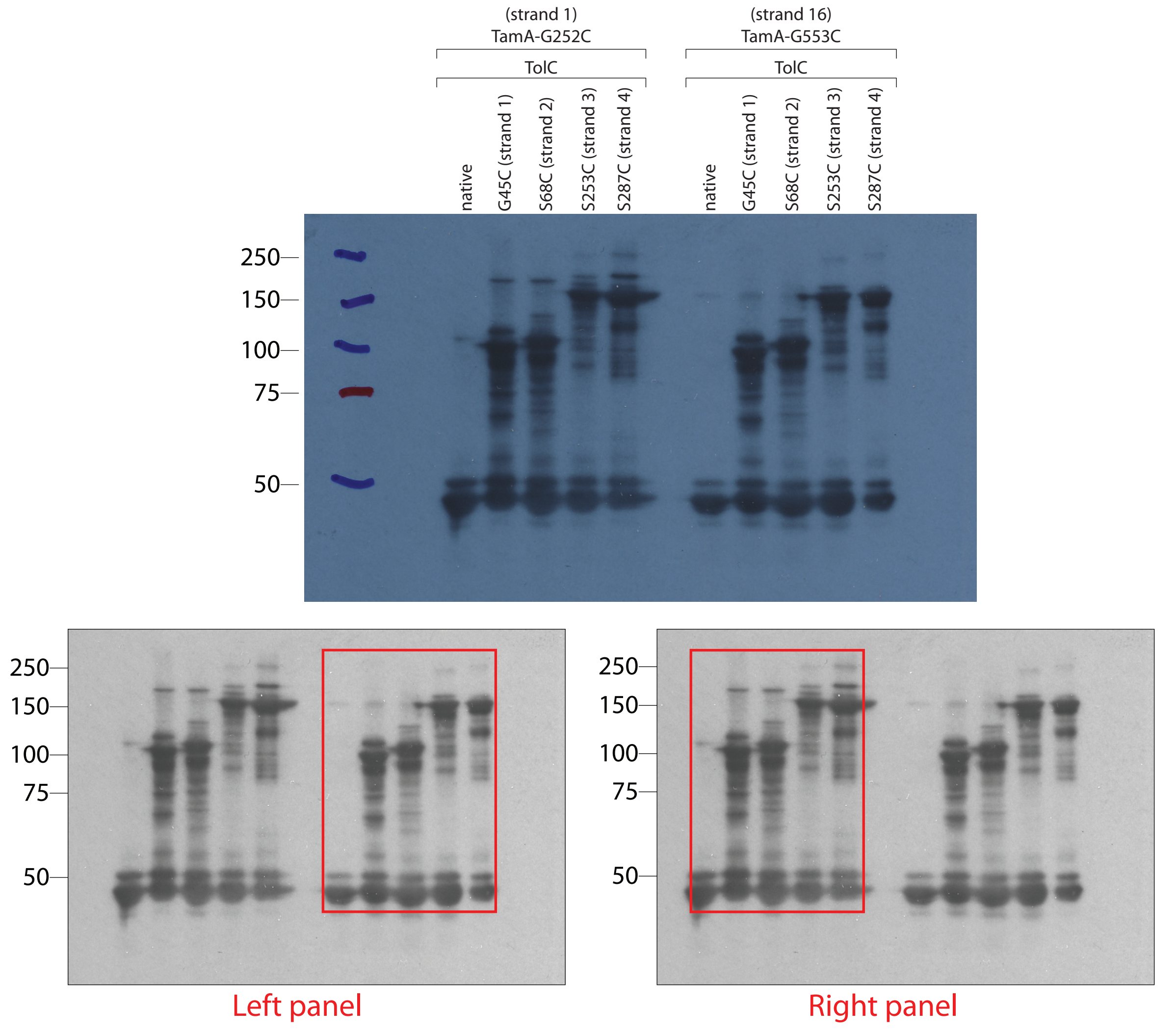

Immunoblot #38 - (S5B Fig; batch 1; αPpiD; left and right panels)

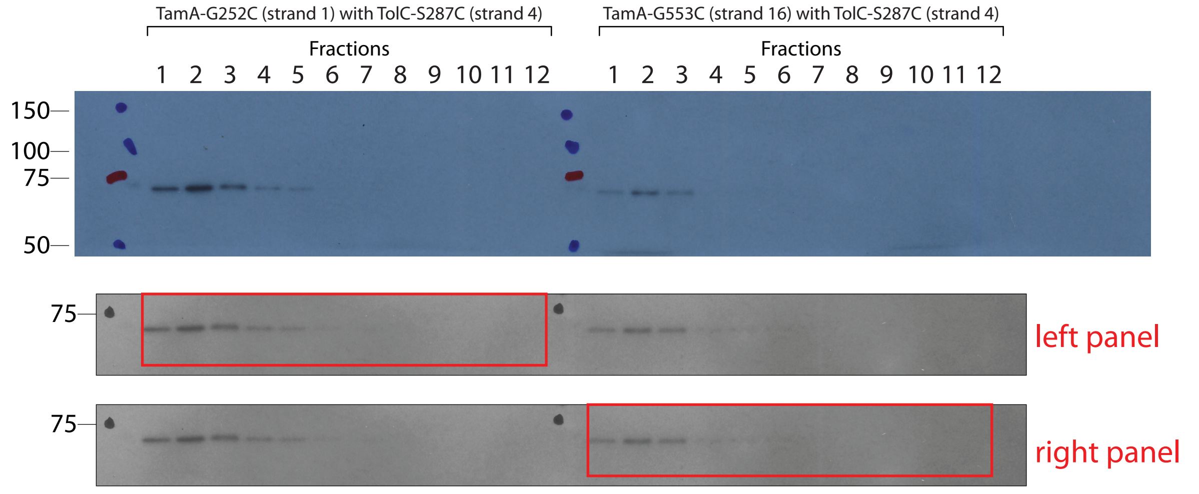

Immunoblot #39 - (S5B Fig; batch 1; αOmpF; left and right panels)

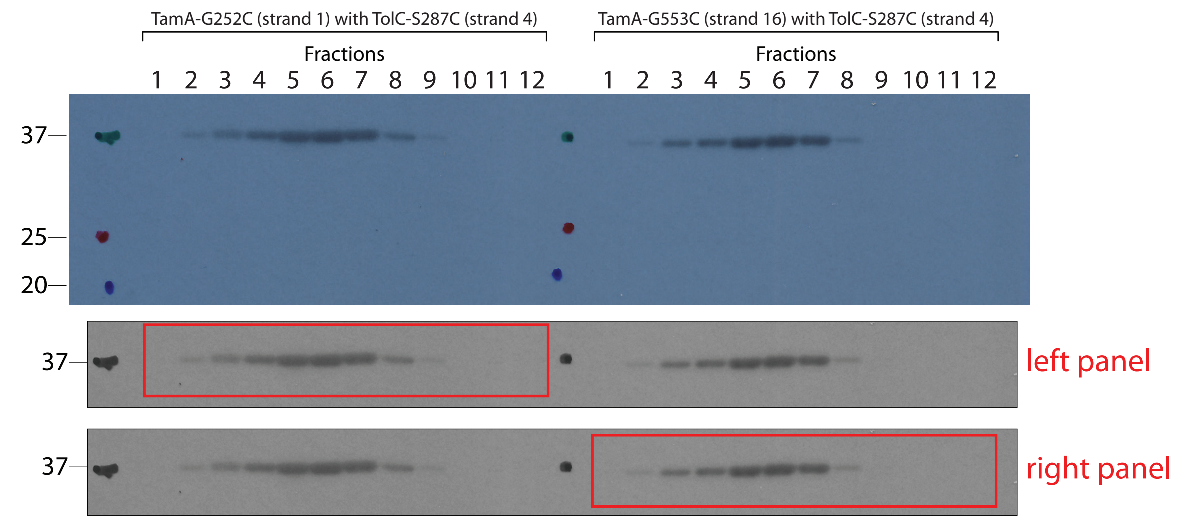

Immunoblot #40 - (S5B Fig; batch 2; αPpiD; left and right panels)

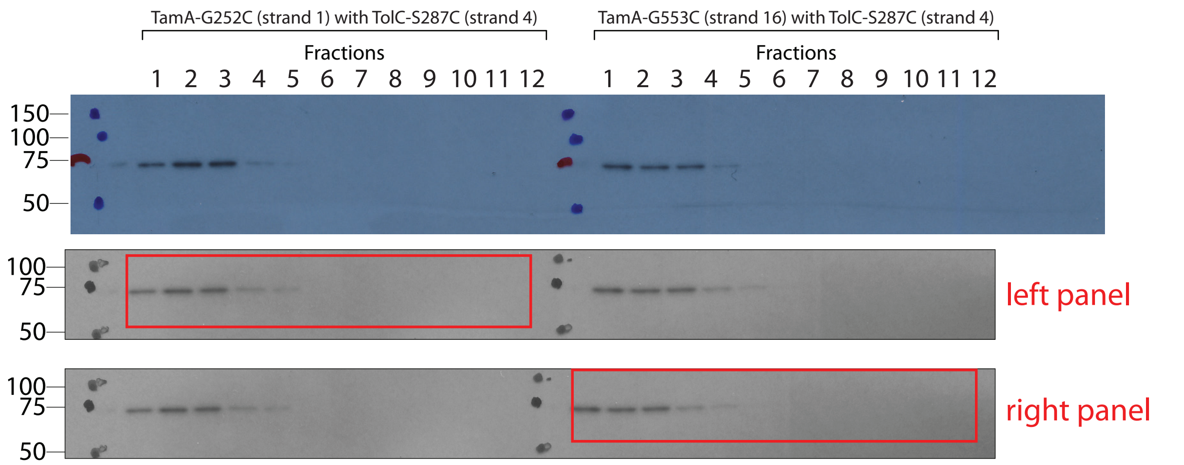

Immunoblot #41 - (S5B Fig; batch 2; αOmpF; left and right panels)

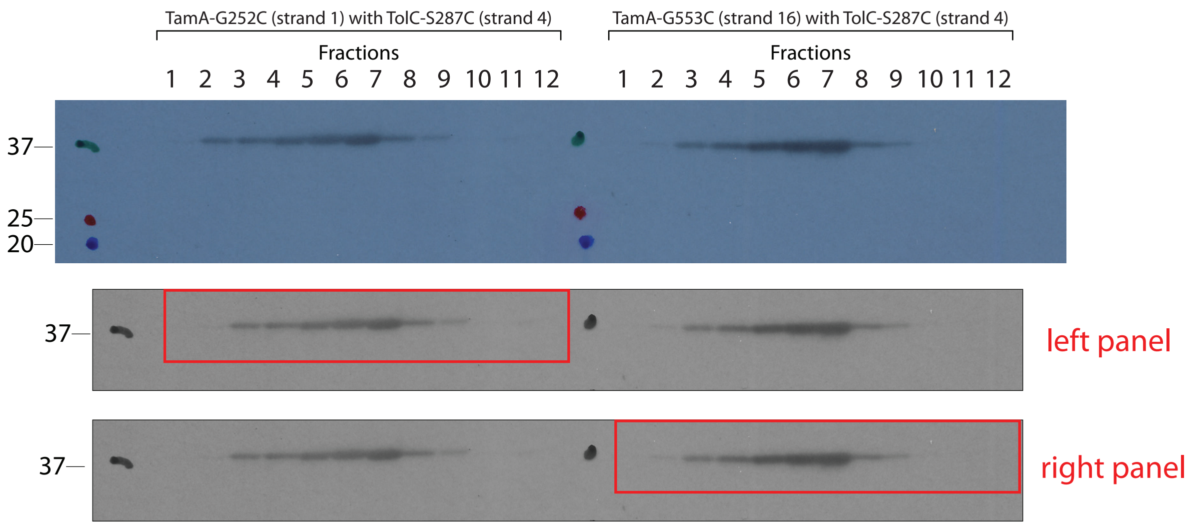

Immunoblot #42 - (S5C Fig; batches 1-2; αTamA)

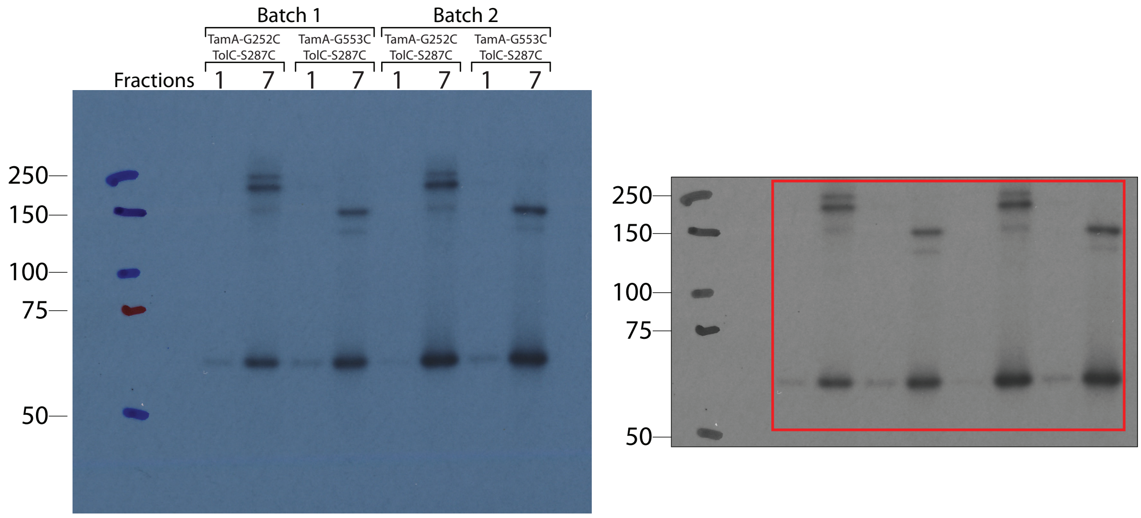

Supplement: S3 Raw Images — Raw image files for all immunoblots. Protein standards markers (where shown) are indicated in kDa to the left of the immunoblots. In some cases, the cropped portion of the figure is shown using a red box and whether the image represents one of the main or supporting information figures is indicated in red text. Otherwise, the immunoblots represent biological replicates. (PDF) [file pbio.3001523.s017.pdf]
