# Supplementary material for: A noncanonical chaperone interacts with drug efflux pumps during their assembly into bacterial outer membranes
Source: PLoS Biol. 2022 Jan 21;20(1):e3001523. doi: 10.1371/journal.pbio.3001523 (PMC8809574; doi:10.1371/journal.pbio.3001523)
Supplement: Attachment — Submitted filename: Response to Reviewers.docx [file pbio.3001523.s018.docx]

**Editor: Paula Jauregui, PhD**

The reviews of your manuscript are appended below. You will see that the reviewers find the work potentially interesting. However, based on their specific comments and following discussion with the academic editor, I regret that we cannot accept the current version of the manuscript for publication. We remain interested in your study and we would be willing to consider resubmission of a comprehensively revised version that thoroughly addresses all the reviewers' comments. We cannot make any decision about publication until we have seen the revised manuscript and your response to the reviewers' comments. Your revised manuscript would be sent for further evaluation by the reviewers.

We consider that you need to conclusively show that TolC insertion into OM is catalysed by TamA in order for us to consider the manuscript forward. Specifically, performing complex immunoprecipitation (Co-IP) would be essential for the current narrative of the manuscript, for the direct demonstration of TolC – TamA interaction as well as of the physiological relevance of this interaction. Please, also address the other concerns from the reviewer, such as the issues from reviewers #1 and #2 with the localization of the proteins and how you distinguish inner membrane, periplasmic or outer membrane components. Also, reviewer #2 and #3 have some concerns and questions about the gels, and reviewer #3 has other concerns such as lack of controls. Please address all the reviewers concerns. 

We appreciate that these requests represent a great deal of extra work, and we are willing to relax our standard revision time to allow you six months to revise your manuscript. We expect to receive your revised manuscript within 6 months.

We appreciate the timeline and have done all of the requested experiments and revised the text according to the reviewers’ expert comments. We agree with the reviewers’ that these comments were directed at improving the manuscript.

**Reviewer #1: Structural Biology, Macromolecular Assemblies and Drug Efflux.

Reviewer #2: Membrane protein folding.

Reviewer #3: Structural biology and host-pathogen interactions.**

**Reviewer #1**: TolC is key component require for efflux pumps, the relocation and success assemble of TolC the required for tripartite functioning in the bacteria. The author provide evidence for possible interactions between TolC and TAM complex, suggest TolC is one of the substrate for the complex and mutations effect on the TolC present on the outer membrane. Overall this paper is well written, provide an indirect evidence of TolC-TAM in vivo interactions. Certain experiments is required to further confirm author's conclusion in the paper.

1) membrane separation result for overall, inner membrane and outer membrane all fractions in the bacteria with quantitive measurement for the target proteins.

We thank the reviewer for this suggestion. We have now included these controls. We show that are membranes are free from cytoplasmic (GroES) and periplasmic (SurA) contaminants in S3 Fig, and perform sucrose density fractionation to demonstrate that the HMW bands are located within the outer membrane enriched fractions (S4 Fig). The previous S3 Fig is now S6 Fig.

2) direct evidence between TAM and TolC use purified protein is required, in vitro CO-IP experiment will be strong evidence support for the conclusion in the research.   

We appreciate the point but unfortunately, and as is the case with more classic chaperones such as Hsp70, the chaperone-client protein interaction is transient and not captured by co-IP. The addition of crosslinking before analysis is to stabilize these transient interactions for analysis. In addition to including a TolC immunoblot (S4 Fig), we have now added a more detailed explanation in lines 201-259 to better explain our cross-linking procedure and better explained the controls used and the meaning behind the crosslinks, as well as

**Reviewer #2**: This manuscript presents evidence for TolC insertion and folding by TamA. Evidence is provided for the pervasiveness of TolC homologs, for the use of a crosslinking assay to determine TamA insertion, for the interaction between TamA and TolC, and finally for the existence of BamA, TamA and TolC homologs in various phyla and classes.

The manuscript is extremely thought provoking and clearly written. I would very much like to see it published here. While the paper is generally clear the discussion of TolC homologs could be further clarified.  Moreover, the gels contain a lot of data some of which is not described in the manuscript.

The authors identify 170k homologs to TolC in a very wide variety of organisms from the pfam OEP fold. Previous work has identified homology between the outer membrane component and the periplasmic component of RND efflux pumps (PMID: 30057025) such that there are proteins that are close homologs of both OEPs and PEPs simultaneously. It is unclear to me if and how the pfam OEP fold distinguishes between the periplasmic and outer membrane components and if there is the possibility that PEPs are being inadvertently being considered OEPs.

We appreciate this suggestion and have incorporated it into the revised text. This reference (PMID: 30057025) posits that TolC-like proteins are evolutionarily related to their membrane fusion protein partners, especially considering that they both form a contiguous alpha-barrel channel. This is important as it adds a challenge to the bioinformatics. Considering the two proteins are evolutionarily related, it is indeed hard to disentangle the two protein families (ie. PEPs and OEPs). To address this, we have added an additional filter to remove proteins that are not secreted: TolC-like proteins (OEPs) should have a signal peptidase I or II dependent signal peptide whereas membrane fusion proteins (PEPs) should have an inner membrane anchor (i.e. no signal peptide) or have a signal peptidase II with an inner membrane retention signal. The additional filter reduced the phylogenetic tree from 518 sequences (OEPs + at least some PEPs) to 416 sequences (OEPs). The more sophisticated analysis did not change the subsequent results or overall conclusions that were based on the tree.

In figure 2 D and E could the authors explain the two bands that periodically disappear and reappear between A and FimD.

The question is best answered when considering S2B Fig. Both bands are present in all blots, but for the sole exception of the top two autoradiographs in S2B Fig (where there is no complementing TamA). This is because the ~60 kDa band corresponds to TamA (which we have now annotated). It is present in the complemented strains (expressing TamA) but not in the non-complemented mutant strains (not expressing TamA). The ~75 kDa band is a protein that has not been characterised. This band appears irrespective of whether there is a plasmid or not, likely corresponding to very residual amounts of protein translated from a particularly stable native mRNA transcript (i.e. long half-life) that is still present even after the 1 h incubation with rifampicin.

In figure 2D and E the authors describe band B as a folding intermediate of FimD. If this were the case, I would have expected B to appear first before the A and C bands, but frequently B appears after A and C. Can the authors give me some intuition for why this would be the case?

We have previously explored this in detail (PMID: 27572967) and have now included this important information in the text at lines 147-159.

Briefly, the intermediate appears when the BAM complex assembles FimD, but not when TAM assembles FimD. Previous timecourse analysis (PMID: 27572967) showed it to be an intermediate that stalls on the BAM complex for up to an hour. Conversely, when the TAM is present to assemble FimD, the reaction is complete in 2-5 minutes and the “band B” intermediate is never accumulated. In Figure 2D/E, the “band B” intermediate is observed when the lateral gate of TamA is not 100% locked under native conditions, as explored in S1 Fig. In the complete absence of TamA (as shown in S2 Fig), it takes a comparatively longer time to generate native FimD (the kinetics of which are detailed in PMID: 27572967).

In Figure 3C, why are there two forms of TolC-TamA for the cys mutants in the first 2 strands, but one form of TolC-TamA for the cys mutants in the latter two strands?

The migration of a protein species on SDS-PAGE depends on protein shape. In the usual samples, all proteins are the same shape (i.e. linear) so migrate as a single species in accordance with their molecular weight. In samples containing cross-linked proteins, different shapes are possible. We use this slide to explain why the HMW bands have different mobilities, and the diagram can be used to explain why there are multiple species. We have also included a better explanation for this in our text at lines 217-227.

Figure A: Theoretical shapes on the gel. The relative position of the cysteine residue in TamA (grey) and/or TolC (white) is shown as a black line that represents the pivot point. This particular structure should be denatured (boiled in SDS) but relatively flexible around the disulfide bond (which does not break without a reducing agent), depending on how the SDS coats the protein. The most prominent structures are likely to be the “T”, “X” and “L” shapes that appear at about 90° intervals or so. The highest HMW band would likely be the bulkier product “X”, “T”, or “L” shape, whereas the lower HMW band would correspond to the “I” shape found when the rotation about the disulfide bond is 0° or 180°.

Also, the authors describe the soluble TolC-TamA bond to the fourth strand of TolC as being attached to a nascent TamA. How can they exclude the possibility that it isn't a nascent TolC to a mature TamA or a nascent TamA to a nascent TolC?

We have now clarified this statement. The first and third possibilities mentioned by the reviewer are actually the same. We were trying to convey that nascent TolC interacts with mature TamA (fully folded and localised to the outer membrane) but because some of the HMW were extracted from the membrane and found in the aqueous phase, this likely meant that the TamA was not yet incorporated into the membrane itself. This has now been clarified at lines 236-238.

In Figure 3D, in contrast to 3C there are two forms of TolC-TamA for the second two strands of TolC binding to TamA and only one form of TolC-TamA when the first two strands of TolC bind TamA. What would cause the switching between two and one form?

See Figure A above. We believe this is about the relative abundance of the theoretical shapes (i.e. some HMW species are more prominent/stable than others).

MIC data should be provided in the supplement if nowhere else.

We have now added this as a S1 Table (former S1-S4 tables are now S2-S5 Tables, respectively)

*Minor concerns:*

The acronym OEP should be defined at its first use.

We have now corrected this.

TolC channel in figure 1A appears somewhat yonic. This is, of course, an artistic decision, but the authors might consider making the inside of the channel the lighter pink and the outside of the channel the darker pink.

We have inverted the colours as suggested.

**Reviewer #3:** Studenrauch, et al. report an investigation of the role of the E. coli TamA, a homolog of BamA, in inserting the major outer membrane component of many bacterial efflux pumps, TolC, into the outer membrane. The study reveals the widespread distribution of TolC in bacteria with an LPS outer membrane, and reveals a combination of both vertical and horizontal evolution. The authors show that the evolution of TolC is somewhat coupled with the Tam and/or Bam systems. Using biochemical experiments, the authors reveal that TamA's lateral gate can be locked into a non-functional state, attenuating insertion of substrate FimD into the outer membrane. The authors then go on to show that TamA can interact with TolC at multiple different residues.

The title of this manuscript and abstract suggests that this work will show that TolC folding/insertion in the outer membrane is performed by TamA, which would be an important advance in the field. However, no evidence is presented indicating that TolC is at all dependent on TamA, so this claim is not supported by the data provided. They do, however, show that TamA modulates the insertion/conformation of FimD in the OM, and that TamA can interact with TolC when they are both over-expressed. While it is conceivable that TamA may indeed insert TolC, these conclusions are not currently well supported by the experimental data presented. Consequently, we think that the claims and framing of the manuscript require considerable reconfiguration (e.g., is this more a story about the lateral gate of TamA than about TolC insertion?), and is not ready for publication in its current form. Additional controls and alternative design of some experiments is required. The specific comments
are as follows:

*MAJOR COMMENTS:*

ln 18-21: authors don't directly show that TolC insertion into OM is catalysed by TamA. As this is the primary claim made in the title and abstract, and it is unsupported, this is a fundamental problem that must be resolved (either substantially changing the claims and narrative, or by providing data to support those claims)

We appreciate the point, and have now modified our title and abstract to reflect the data as presented. The title now reads: *“A non-canonical chaperone interacts with drug efflux pumps during their assembly into bacterial outer membranes.”*

*MINOR COMMENTS:*

ln 67: mention that TolC is topologically different to other substrates of BAM complex - should expand on how they are different. Is this referring to the mix of porin and helical periplasmic domain? To the porin split into three parts?

We have now clarified this statement by adding “because their β-barrel fold is not evolutionarily related to other outer membrane proteins, but instead evolved from the periplasmic component of the efflux pump, with convergent evolution bringing it to resemble the β-barrel fold [30].” The cited reference examines the details and provides evidence to the convergent evolution of the two types of β-barrels.

ln 86: Could include more background on TamA - for example could expand and say there is already data that suggest it inserts proteins into the outer membrane? I don't think they actually ever say that TamA is a homolog of BamA.

We thank the reviewer for this assessment and have now added more background information to provide better introductions to the TAM and the BAM complex (lines 68-77; 88-98).

Fig. 1C: what is an OEP domain? Should specify in figure legend and main text (not just in methods)

We have corrected this. In that figure legend and in the main text at line 263.

Fig. 1D: Label orange arrow something like "E. coli TolC" on figure?

We have now corrected this. Fig 1D has also now been moved to Fig 4A as part of an expanded bioinformatics analysis.

ln 118-122: This is very interesting. If involved in translocating molecules across the S-layer, one might expect the "Omp" portion of TolC to be quite different since it would no longer be membrane spanning. Does anything jump out in this analysis? Alternatively, might many/all of the "monoderm" species encoding TolC form spores, and perhaps these TolC proteins insert into the spore OM? I think C. difficile at least forms double-membraned spores.

We agree with the reviewer, this is very interesting, and have expanded on this aspect of the manuscript in response to the comments. We have moved the bioinformatics analysis to be the third results section and included extra panels alongside our phylogenetic tree (now Fig 4A, formerly Fig 1D) that focus in on the TolC-like proteins encoded within monodermic Firmicutes (Fig 4B-C, S5 Fig). In terms of bacterial spores, the reviewer refers to the exosporium layer, which is a proteinaceous layer. While bacterial spores have a double membrane, the “outer” membrane is derived from the mother cell cytoplasmic membrane during asymmetrical division and the coat layer and exosporium are incorporated on top of the outer membrane by the mother cell, before the matured spore is released. The exosporium is not found in B. subtilis, but is found in B. cereus, B. anthracis, and of course, C. difficile (see: Stewart 2015 doi:10.1128/MMBR.00050-15.; and for an excellent depiction of the structural layers of the endospore: Paredes-Sabja et al. 2014, doi: 10.1016/j.tim.2014.04.003).

To address this we have expanded the section with three lines of evidence to support the hypothesis that these TolC-like proteins likely reside within the S-layer. Two publications on *C. difficile* noticed that when the S-layer is shed, so is the protein (which we now identify as the TolC-like protein) (refs 33, 35 in text). As part of Fig 4B, we focused more closely on 5 different families of monodermic Firmicutes and noticed that the presence of the TolC-like protein was independent of the ability to form endospores, for example TolC-like proteins are encoded within 4/12 non-endospore forming bacteria, including *Peptoclostridium acidaminophilum* (see: Galperin et al. 2016, doi: 10.1099/ijsem.0.001548). Finally, the homology model of the *C. difficile* TolC homologue revealed that the surface of the predicted β-barrel domain was not hydrophobic like it is for *E. coli* making it unnecessary for it to sit in a membrane environment (S5 Fig).

ln 124-177: I would find it helpful to have the overall experimental design for the pulse-chase laid out a bit more clearly (and/or a schematic in Fig. 2). The Cys locking aspect was clear, and the differential protease sensitivity was clear. But what is being pulsed and why, and the purpose of the chase aren't stated, and required some detective work to figure out. The chase doesn't really seem to be doing much?

We have now added a schematic of this (S2A Fig). The chase step adds excess [^32^S]-methionine and -cysteine so that [^35^S]-methionine and -cysteine can be tracked. Using this radiolabelled system for pulse chase a clear label is incorporated with the need for only very little amount of protein, avoiding potential issues that would come if the protein had been over-expressed. This has been presented in detail elsewhere (PMID: 27572967) and we cite the paper and now offer a more thorough explanation in S2A Fig legend. We thank the reviewer for raising this issue.

Also, text refers to bands of ~40, 45, and 50 kDa, but the markers don't really match this. Perhaps the uppermost band should be called ~47 kDa instead of 50? It seems like the pulse-chase is being down in BL21 while over-expressing? I think that should be made more clear in the main text, I think I had to look up past papers to figure out this in a more native context.

We have now expanded this aspect of the manuscript at lines 147-159. The molecular mass of each of the bands were previously determined by mass spectrometry which better reflects the sizes of the fragments than its mobility by SDS-PAGE. This has been presented in detail elsewhere (PMID: 27572967) and we explain and cite the paper.

ln 138: should this be an "unlocked" conformation rather than "unlockable"? If it's truly unlockable, then why is that? Using "unlockable" for both the unlocked form of double cysteine mutants but also to refer to single cysteine mutants is confusing. Perhaps just use "locked" and "unlocked" instead.

We agree and have modified the text as suggested.

Alternatively, could force the protein to be fully locked by promoting disulfide bond formation. For example, see this reference for similar experiments done with BamA using CuSO4 and copper-o-phenanthroline (see Noinaj et al, Structure, 2014).

We appreciate this point and have tried to titrate the amount of CuSO_4_ to better stimulate the oxidisation reaction, but this just returned blank phosphor screens. We optimized the amount of CuSO_4_ to show slightly more fragment B appearing (especially in TamA-G252C/G553C) (see S3C Fig). It is unclear to us why the TamA gate is more difficult to close completely with CuSO_4_, while the BamA gate was more readily closed in the Noinaj et al paper.

ln 138: should be "S1 fig" rather than "S1 data"?

We have now corrected this mistake

ln 166: I don't understand how FimD can still be cleaved into a 45 kDa sized fragment in the absence of TamA.

We have now explained this better (Lines 147-159). Briefly, the intermediate appears when the BAM complex assembles FimD, but not when TAM assembles FimD. Previous timecourse analysis (PMID: 27572967) showed it to be an intermediate that stalls on the BAM complex for up to an hour. Conversely, when the TAM is present to assemble FimD, the reaction is complete in 2-5 minutes and the “band B” intermediate is never accumulated. In Figure 2D/E, the “band B” intermediate is observed when the lateral gate of TamA is not 100% locked under native conditions, as explored in S1 Fig. In the complete absence of TamA (as shown in S2 Fig), it takes a comparatively longer time to generate native FimD (the kinetics of which are detailed in PMID: 27572967).

Fig. 2: Would be nice to have the WT control as a reference here in the main fig.

We thank the reviewer for their suggestion, but to also incorporate the comment re: line 124-177 (of the original manuscript), we have used a Supplementary figure, by expanding S2 Fig, to include side-by-side the pulse chase schematic (S2A Fig) and also the single timepoint panels with WT controls (S3C Fig).

Fig 2C and D: It looks like the different gels have different exposures. It would be better to have samples be compared on the same gel/phosphor screen. This is probably more important than having all the time points, as most of the time points look very similar. Band B is indeed enriched in the "locked" mutants, but I do not believe it is completely absent in some of the single mutants. This is ok because TamA could have a backlog of FimD that hasn't been inserted yet - but this should be acknowledged?

We have now included single timepoint panels in S3C Fig. As mentioned above, we have better explained the process Lines 147-159. The backlog hypothesis would be relevant in other systems using protein “over-expression”, but this [^35^S]-labelling pulse-chase system does not overexpress the proteins (PMID: 27572967). The simplest explanation with this system is that the BAM complex is always present and capable of assembling FimD (i.e. going through the intermediate that generates the 45 kDa fragment B), although FimD is a better client/substrate for the TAM.

Seeing all 3 biological replicates somewhere for these experiments would also help.

We have now included this in S2 Raw Figure.

ln 171-172: authors state that in the double mutants that FimD is predominantly the 45 kDa band and there are small amounts of the 40/50. The 45kDa one is enriched, but there is still plenty of 40/50.

We have made this clearer by more explicitly stating that these 40/50 kDa bands are due to the presence of the ‘unlocked’ and functional TamA molecules still being present (see lines 163-166).

lns 210-212 - not sure I agree that all mutants can interact - the gels are a bit messy and I only really find S287C to be the very clear one.

We have better explained this section now (Lines 201-227). We have also included black arrows pointing out the higher molecular weight bands.

Figure 3C and 3D experiments: The gels do not have controls to confirm that band shifts are due to TolC interaction.

We thank the reviewer for this. The gels do have controls and we have now expanded this section to better indicate the use of these controls. See lines 201-227.

If authors can obtain antibodies against TolC (or against its tag), this would make the result more believable. This would allow us to compare TolC levels in the input, which is currently missing. Furthermore, it is surprising to me that all of these mutants would form disulfides to some degree.

We agree and have since prepared antibodies raised against TolC. These antibodies are very useful reagents and we have repeated the necessary experiments using this new antibody reagent. This allowed us to now document the comparison of TolC levels in the input. Unfortunately, there are TolC-TolC bands that obfuscate TolC-TamA bands. While some relevant HMW bands can be identified using our TolC antibody (S4 Fig), others had to be identified by taking a different approach that required the use of native TolC to emphasise the HMW bands that only appeared when TolC-cysteine mutants were introduced.

Perhaps some controls are needed to make sure this reflects protein-protein interactions and not just co-over-expression in periplasm? Ln 225-228 might be consistent with this sort of problem.

We have taken the advice and now provide detailed assessment of the purity of the isolated membranes. The membrane fractions are pure and free from cytoplasmic and periplasmic contaminants (S3 Fig) and that the HMW bands are found exclusively in the outer membrane fraction (S4 Fig). This complements the finding that the HMW bands themselves are integrally associated with the membrane (i.e. because they are found within the detergent phase, Fig 3C-D). The controls reveal that other, control integral membrane proteins like BamA are found exclusively within the detergent phase.

Fig. 4: More "data" than discussion? Maybe this should be presented in the context of other bioinformatic analyses?

We appreciate the advice and have now revised the text so that there is a more substantial bioinformatics section (as part of our third results section) to better contextualize the findings.
